# Immunotherapy in hematologic malignancies: achievements, challenges and future prospects

**DOI:** 10.1038/s41392-023-01521-5

**Published:** 2023-08-18

**Authors:** Lu Tang, Zhongpei Huang, Heng Mei, Yu Hu

**Affiliations:** 1 Institute of Hematology, Union Hospital, Tongji Medical College, Huazhong University of Science and Technology, 430022 Wuhan, China; 2 Hubei Clinical Medical Center of Cell Therapy for Neoplastic Disease, 430022 Wuhan, China; 3 Key Laboratory of Biological Targeted Therapy, the Ministry of Education, 430022 Wuhan, China; 4 Hubei Key Laboratory of Biological Targeted Therapy, Union Hospital, Tongji Medical College, Huazhong University of Science and Technology, 430022 Wuhan, China

**Keywords:** Cancer therapy, Immunotherapy

## Abstract

The immune-cell origin of hematologic malignancies provides a unique avenue for the understanding of both the mechanisms of immune responsiveness and immune escape, which has accelerated the progress of immunotherapy. Several categories of immunotherapies have been developed and are being further evaluated in clinical trials for the treatment of blood cancers, including stem cell transplantation, immune checkpoint inhibitors, antigen-targeted antibodies, antibody-drug conjugates, tumor vaccines, and adoptive cell therapies. These immunotherapies have shown the potential to induce long-term remission in refractory or relapsed patients and have led to a paradigm shift in cancer treatment with great clinical success. Different immunotherapeutic approaches have their advantages but also shortcomings that need to be addressed. To provide clinicians with timely information on these revolutionary therapeutic approaches, the comprehensive review provides historical perspectives on the applications and clinical considerations of the immunotherapy. Here, we first outline the recent advances that have been made in the understanding of the various categories of immunotherapies in the treatment of hematologic malignancies. We further discuss the specific mechanisms of action, summarize the clinical trials and outcomes of immunotherapies in hematologic malignancies, as well as the adverse effects and toxicity management and then provide novel insights into challenges and future directions.

## Introduction

Cancer immunosurveillance is a process in which multiple innate and adaptive immune effector cells and molecules are involved in the recognition and killing of cancer cells.^1^ Extrinsic immune stress can either prevent tumor growth, development and survival or promote tumor growth by both sculpting the immunogenicity of the tumor or inhibiting the anti-tumor immune response.^1,2^ Immune editing is considered one of the key parts of why tumors could evade the surveillance and lie dormant in the host body for years before re-emerging through the “equilibrium” and “senescence”.^3^ With the growth of poorly-immunogenic variants and the destruction of the host immune system, cancer cells ultimately evade immunosurveillance.^4^ Cancer cells employ many strategies to suppress the immune system of the human body, so that they can survive in every stage of the anti-tumor immune responses.^5^ The generation of anti-tumor immune response is a complicated and multi-step process and Chen et al. refer to these steps as the “Cancer-Immunity Cycle”.^6^ As for cancer patients, the “Cancer-Immunity Cycle” does not perform optimally. Any abnormality in these steps can lead to the failure of the “Cancer-Immunity Cycle” and consequent cancer immune evasion.^7^ Immunotherapies could fight against cancer by harnessing the immune system and restoring anti-tumor immunity.^8^ Constructed over decades, immunotherapies have begun to demonstrate such promising results in treating cancer patients and have been selected as the “Breakthrough of the Year for 2013”.^8–10^

Hematologic malignancies refer to malignant diseases originating from the lymphohematopoietic system and may involve all systems and organs throughout the body. Hematologic malignancies mainly include acute leukemia, chronic leukemia, lymphoma, multiple myeloma (MM), myelodysplastic syndrome (MDS), and myeloproliferative neoplasm (MPN). Acute lymphoblastic leukemia (ALL) is characterized by the abnormal proliferation of a huge number of immature lymphocytes.^11^ Acute myeloid leukemia (AML) is the most commonly occurring acute leukemia in adults and its incidence increases with age. As a result of genetical mutations in hematopoietic stem/progenitor cells, AML is a highly heterogeneous disease.^12,13^ Lymphomas are typically divided into two categories, Hodgkin lymphoma (HL, which accounts for about 10% of all lymphomas) and non-Hodgkin lymphoma (NHL).^14^ NHL is the most prevalent kind of lymphoma arising from lymphocytes that are at various stages of development and the characteristics of the specific lymphoma subtype reflect those of the cell from which they originated.^14^ Diffuse large B-cell lymphoma (DLBCL), mantle cell lymphoma (MCL), and follicular lymphoma (FL) represent the most common types of NHL. HL, also known as Hodgkin’s disease, is a rare type of lymphoma with unique histologic, immunophenotypic and clinical features.^15,16^ HL consists of two discrete disease entities: classical HL (cHL), which accounts for the majority of HL cases and nodular lymphocyte predominant HL.^16^ MM, MDS and MPN are most common in elderly patients. MM accounts for about 10% of hematologic malignancies and cannot currently be cured. It typically begins as an asymptomatic precursor, either a monoclonal gammopathy of undetermined significance or smoldering multiple myeloma.^17^ MDS is a clonal disorder characterized by ineffective hematopoiesis and a tendency to evolve into AML.^18^ With increasing advances in chemotherapy, radiotherapy and targeted therapy, the overall response rate (ORR) of cancer patients has improved significantly. Historically, multi-drug chemotherapy has been the cornerstone of the treatment of both pediatric and adult patients with hematologic malignancies. However, over the past decade, many patients still face treatment failure due to relapse and resistance. The molecular characteristics of hematologic malignancies are highly heterogeneous, leading to considerable challenges in precision medicine and individualized treatment.

With the potential to induce long-term remission in patients with refractory or relapsed (R/R) hematologic malignancies, immunotherapy has already led to a paradigm shift in cancer therapy and tremendous success in the clinic. Furthermore, hematologic malignancies in this setting have some unique characteristics that make these cancers well-suited as targets for immunotherapy.^19^ Immune cells and cancer cells are in constant interconnection with each other within the hematopoietic system, enabling an environment that is conducive to immune surveillance. Since the cellular origins of malignancies are the same as that of the immune system, the nature of these cancer cells is immunostimulatory. However, this may meanwhile lead to deficit and hindered immune responses. There has been accelerating advancement of cancer immunotherapies based on various strategies to harness the host immune system. Different immunotherapeutic approaches have their advantages but also shortcomings that need to be addressed. This review will provide perspectives on the applications and clinical considerations of immunotherapies so that clinicians can acquire timely information about such revolutionary therapeutic options. Here, we first outline the recent advances made toward understanding multiple categories of immunotherapies in the treatment of hematologic malignancies. We further discuss the specific mechanisms of action, summarize the clinical trials and outcomes of immunotherapies in hematologic malignancies, as well as the adverse effects (AEs) and toxicity management and then provide insights into future directions.

## The history of immunotherapy in hematologic malignancies

**Fig. 1:**
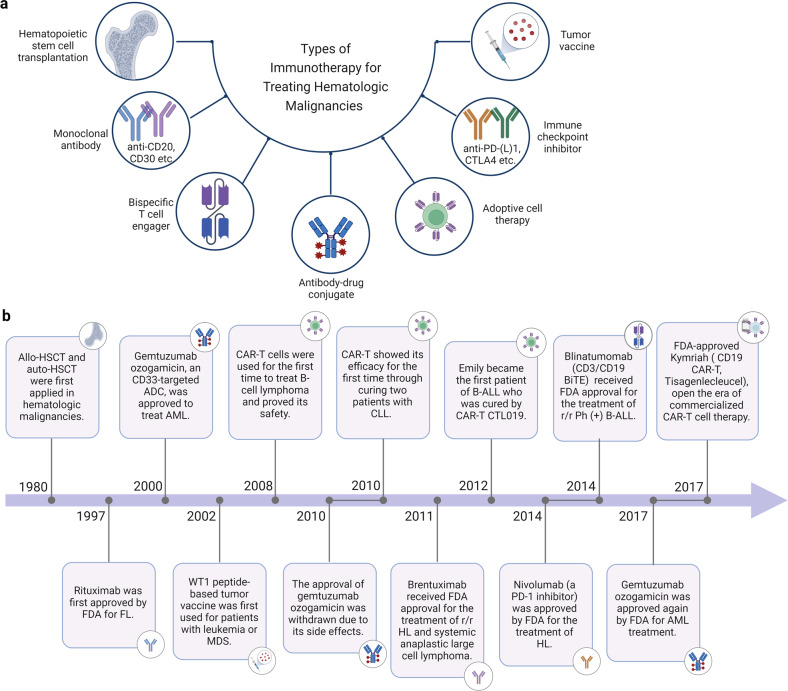
The development of immunotherapy for hematologic malignancies. **a** Types of immunotherapies for treating hematologic malignancies. **b** The journey of the history of immunotherapy for hematologic malignancies

As for the field of treating hematologic malignancies, immunotherapy mainly involves targeted antibodies, immune checkpoint inhibitors (ICIs), tumor vaccines, adoptive cell therapy (ACT), and stem cell transplantation (Fig. 1a). The journey of the history of immunotherapy for hematologic malignancies is summarized in (Fig. 1b). The allogeneic hematopoietic stem cell transplantation (allo-HSCT) is one of the oldest forms of cancer immunotherapy.^20^ The allo-HSCT was first applied to disease treatment in 1968 by E. Donnall Thomas, who would later win the Nobel Prize for being a pioneer in this technology and is praised as “the father of stem cell transplantation”.^20^ The allo-HSCT was primarily performed for treating leukemia in 1975 and lymphoma in 1978. Since then, HSCT has been used worldwide to treat serious blood disorders. Although it has been referred to as “the bluntest weapon of chemotherapists”, as it indeed aims to eradicate and restore the hematopoietic and immune systems, it still occupies a pivotal position and gives patients the possibility of a cure. It wasn’t until the end of the 20th century that new immunotherapy approaches emerged. Rituximab, a kind of anti-CD20 monoclonal antibody (mAb), was the first to be approved by the United States Food and Drug Administration (FDA) for the treatment of cancer in 1997 and since then has become the prototype for anti-CD20 mAbs and the backbone treatment regimen for B-cell malignancies, such as DLBCL, CLL (chronic lymphoblastic leukemia) and FL.^21^ As well, the rituximab, combined with CHOP (cyclophosphamide, doxorubicin, vincristine, and prednisone) regimen, has become the first-line therapy for patients with NHL.^22^ Meanwhile, more types of mAbs have been developed, such as tafasitamab (anti-CD19 mAb) for DLBCL,^23^ daratumumab (anti-CD38 mAb) for MM,^24^ and lintuzumab (anti-CD33 mAb) for AML.^25^ However, for R/R patients, mAbs often lose their clinical effectiveness and the development of bispecific antibodies (bsAbs) may allow for the continuation of treatment. Blinatumomab, an anti-CD3/CD19 BiTE (bispecific T cell engager), was the first FDA-approved BiTE for the treatment of R/R precursor B-cell ALL (pre-B-ALL) and has also achieved remarkable curative effects.^26^ Over the past several decades, antibody-drug conjugates (ADCs) have been evaluated in a variety of clinical trials of hematologic malignancies. The brentuximab vedotin was approved by the FDA in 2011 for treating relapsed HL and systemic anaplastic large cell lymphoma (SALCL).^27,28^ WT1 (Wilms’ tumor gene 1) peptide-based tumor vaccine was first used in patients with overt leukemia from MDS or MDS with myelofibrosis in the year 2002.^29,30^ As another rising star in immunotherapy, ICIs have entered the field of treatment for hematologic malignancies due to their great success in solid tumors. PD-1/PD-L1 (programmed death receptor 1, programmed death receptor ligand 1) inhibitors play a notable clinical role in B-cell lymphoma, especially in HL.^31^ CTLA-4 (cytotoxic T-lymphocyte antigen number 4) inhibitor also demonstrates certain curative effects in patients with HL and AML.^32^ There’re lots of clinical trials of these drugs applied to different kinds of hematologic malignancies to overcome resistance and relapse. ACT is the most popular immunotherapy for patients with R/R hematologic malignancies, such as TCR-T (T cell receptor-engineered T) cell, γ/δ-T (gamma/delta T) cell, NK (nature killer) cell and CAR-NK (chimeric antigen receptor nature killer) cell and especially CAR-T (chimeric antigen receptor T) cell therapy.^33–35^ Fred Hutchison Cancer Institute used CAR-T cells for the first time to treat B-cell lymphoma and proved its safety in the year 2008. And in the year 2010, two patients with CLL first received CAR-T transfusion and achieved CR (complete remission) and the CAR-T cells were still detected in vivo after 10 years of follow-up.^36^ In 2012, Emily, an American patient with B-ALL, received CAR-T therapy and was cured. She has been disease-free for almost 11 years up to now. The development of CAR-T therapy has been greatly boosted due to the launch of large clinical trials, such as axicabtagene ciloleucel and tisagenlecleucel, as well as the FDA’s approval of the first commercialized CAR-T cell product in 2017. At present, CAR-T therapy has achieved remarkable results in R/R ALL, CLL, NHL, and MM.^37^ There are many CAR targets for each malignant disease and the number of treatment lines is gradually advancing. In summary, immunotherapy has achieved rapid development in recent years, which provides more possibilities and hopes for the cure of hematologic malignancies.

## Overview of immunotherapies in hematologic malignancies

### HSCT

HSCT is an effective means of curing a range of hematologic diseases. It is done by harvesting functional hematopoietic stem cells from the patients or a healthy donor and transplanting them to the patients to replace their dysfunctional blood system. Initially, bone marrow was considered as a source of stem cells for transplantation. However, within the last two decades, peripheral blood stem cells have replaced bone marrow stem cells and become the main stream.^38^ The replacement indicates no impact on overall survival (OS) except a greater risk of graft-versus-host disease (GVHD).^38^ Fortunately, the management of GVHD is strict and upgraded continuously.^39^ The allo-HSCT is usually considered as a preferred choice for hematologic malignancies.^40,41^ But due to the greater risk of GVHD, allo-HSCT is still restrictive to the patient’s own status. This led to the emergence of reduced-intensity stem cell transplantation (RIST), which is associated with less morbidity and mortality and can be performed in a wider range of patients.^42,43^ Meanwhile, cord blood transplantation with a low relapse rate and chronic GVHD was also promoted but was later hampered by a high incidence of infection and transplant-related mortality. However, the safety and feasibility of HSCT using single UM171-expanded cord blood were validated in patients with malignant hematologic diseases who did not have a suitable HLA (human leukocyte antigen)-matched donor, indicating the potential to overcome the disadvantages of other cord blood transplantation while maintaining the benefits of low risk of chronic GVHD and relapse.^44^ Haploidentical family donors, such as parents, children, or haploidentical siblings, offer the advantage of rapid donor availability. Currently, two methods are most commonly used for haploidentical hematopoietic stem cell transplantation (haplo-HSCT): (i) granulocyte colony-stimulating factor (G-CSF) plus anti-thymocyte globulin-based regimen with non-manipulated T-cell enriched grafts, which was originated by the Peking group in China; (ii) post-transplantation cyclophosphamide-based regimens with non-manipulated T-cell enriched grafts, which was initiated by the Baltimore group in the United States.^35,45–47^ With the development of haplo-HSCT, strategies to address the associated side effects have become a research trend. A substantial improvement in non-relapse mortality and supportive care (e.g., treatment and prevention of infections or GVHD) has contributed to improved OS of allogeneic transplantation over the past decades.^48,49^ In addition, to overcome barriers such as donor availability, novel transplantation strategies have been refined. For example, post-transplant cyclophosphamide for GVHD prevention after haploidentical donor transplants has shown similar outcomes with a reduced risk of GVHD.^50,51^ The recurrence of the malignancy remains the most prevalent cause of post-transplant failure or even death, emphasizing the importance of enhancing the immune system in the treatment of hematologic malignancies and how far we have yet to go to achieve a cure. Although much is still being discovered, we have learned a great deal about how the host immune system affects the treatment of hematologic malignancies from the growing and evolving field of allogeneic transplantation, which is helping to advance the field of novel immunotherapies.^20^

### mAbs

The mAbs are highly homogeneous IgG antibodies produced from a single B cell clone and directed against only specific antigenic epitopes. The first-generation mAbs are derived from mice and typically prepared using the hybridoma technique, which is based on cell fusion technology that fuses sensitized murine B cells with the capacity to secrete specific antibodies and myeloma cells with the capacity to multiply indefinitely into B cell hybrids.^52^ Through culturing individual hybridoma cells with such properties into cell populations, it is possible to generate antibodies against corresponding antigenic epitopes. However, murine mAbs can be recognized by the immune system and result in human anti-mouse antibody reactions, particularly human anti-mouse antibody (HAMA),^53–55^ resulting in limited efficacy of mAbs and potentially serious AEs. Since then, mAbs have gradually evolved toward the trend of humanization. The second generation is human/mouse chimeric mAbs (with the suffix -ximab, e.g., rituximab),^21^ using chimeric antibody or humanized modified monoclonal antibody technology.^56,57^ Both approaches greatly reduce the human anti-mouse immune response, but a certain degree of immunogenicity still exists because they contain mouse-derived sequence fragments. The subsequent mAbs are fully humanized (with the suffix -zumab and -mumab), with the amino acid sequences that make up the antibodies all derived from humans. These mAbs are mainly manufactured by phage display screening,^58,59^ yeast surface display,^60,61^ human hybridoma technology and single B-cell antibody preparation technology,^62^ or even metabolic strategy like glycoengineering.^63^ Meanwhile, these mAbs have a 100 percent human component and reduced immunogenicity, although they may still have immunogenicity due to anti-idiotype antibodies.

**Fig. 2:**
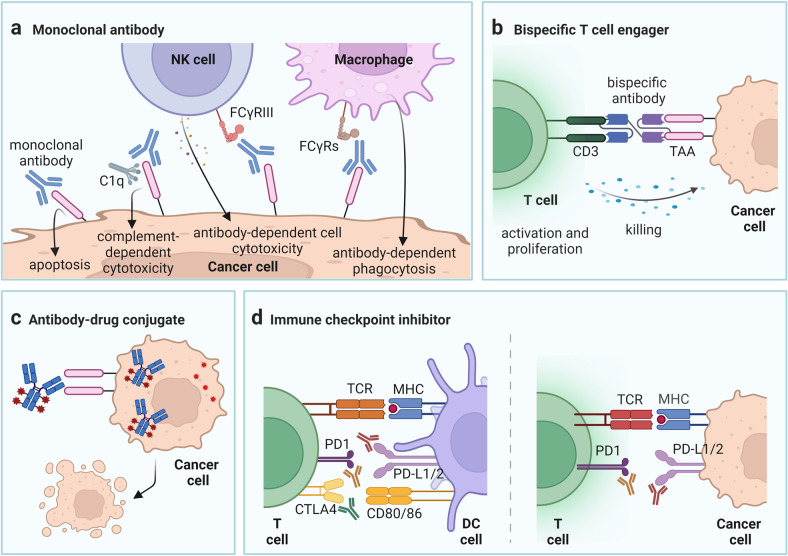
Mechanisms of action of four kinds of immunotherapy drugs. **a** The monoclonal antibodies (mAbs), when combined with their targets, can kill cancer cells by direct induction of apoptosis through programmed cell death, antibody-dependent cell cytotoxicity, complement-dependent cytotoxicity, and antibody-dependent macrophage-mediated phagocytosis. **b** The BiTE ((bispecific T cell engager) molecule usually targets one CD3 molecule and one tumor antigen simultaneously. Thus, in addition to the anti-cancer role of the tumor antigen-targeted antibody, it can promote the activation and recruitment of CD3 + T cells. **c** After bound to the tumor surface antigen, the antigen undergoes endocytosis and the antibody-drug conjugates (ADCs) will be internalized into the tumor cell and subsequently transported to the lysosome to release the cytotoxic payload, which can induce apoptosis and kill surrounding cancer cells through bystander effects. **d** The blockade of PD-1 or its ligands PD-L1 and PD-L2 can help to restore the anti-tumor immunity of the body and simultaneously enhance the lysis effect of cytotoxic T cells to achieve the effect of tumor eradication. CTLA-4 inhibitors can block the binding between CTLA-4 molecule and B7 during T cell activation, increase the level of the recognition of T cells to tumor-associated antigens (TAAs) and enhance the anti-tumor responses of the body’s immune effector cells

The mAbs are the major component of cancer immunotherapy.^64^ mAbs have various mechanisms of action and each type of antibody has multiple mechanisms of action in parallel, mobilizing multiple aspects and components of immunity to ultimately kill tumor cells. The mAbs, when combined with their targets, can kill cancer cells in two ways (Fig. 2a): (i) direct induction of apoptosis through programmed cell death (PCD);^65^ (ii) immune-mediated mechanisms, mainly including antibody-dependent cellular cytotoxicity (ADCC), complement-dependent cytotoxicity (CDC) and antibody-dependent macrophage-mediated phagocytosis due to the binding of Fc and FcγR (Fc gamma receptor).^65–70^

Rituximab is a first-generation anti-CD20 mAb. Ofatumumab is one kind of second-generation, fully-humanized anti-CD20 mAb that binds to a different site from rituximab and was approved by the FDA for the treatment of CLL in 2009, as well as in combination with chlorambucil for the treatment of CLL in 2014.^71,72^ Obinutuzumab is another second-generation anti-CD20 mAb and was approved by the FDA in combination with chlorambucil for the treatment of CLL in 2013 and in combination with bendamustine for the treatment of R/R FL in 2016.^73,74^ Daratumumab is an anti-CD38 mAb that was FDA-approved for the treatment for patients with MM.^24^ Elotuzumab is an anti-CS1 mAb that was approved by FDA in combination with lenalidomide and dexamethasone for the treatment of R/R MM in November 2015.^20^ Furthermore, the FDA-approved mAbs, such as daratumumab,^75–78^ elotuzumab,^79–81^ and isatuximab,^82–84^ have already revolutionized the standard of care for treatment of MM, or even in the front-line therapeutic setting. Up to now, as presented in Table 1, many kinds of mAbs have been developed for the treatment of hematologic malignancies with their targets involving CD20, CD19, CD22, CD38, CS1 (SLAMF7), CD52, CD40, CD80, CD74, and CD33.^25,74–114^

**Table 1:** Representative antibody-based drugs used for treating hematologic malignancies

Type	Drug	Target	Indication	If FDA approved?	Refs.
mAbs	Rituximab	CD20	B-NHL	Yes	^85–88^
	Ofatumumab	CD20	CLL	Yes	^90^
	Obinutuzumab	CD20	DLBCL, MCL, FL, CLL	Yes	^74,92–95^
	Ibritumomab tiuxetan	CD20	B-NHL	Yes	^89^
	Veltuzumab	CD20	B-NHL, CLL	No	^91^
	Ocrelizumab	CD20	FL	No	^96^
	Ocaratuzumab	CD20	B-NHL, CLL	No	^97–99^
	Ublituximab	CD20	CLL	No	^354^
	Epratuzumab	CD22	B-NHL	No	^114^
	Tafasitamab	CD19	DLBCL, FL	Yes	^23,352^
	Inelituzumab	CD19	B-NHL	No	^112^
	Galiximab	CD80	FL	No	^113^
	Alemtuzumab	CD52	PTCL, CLL	Yes	^100,101^
	MDX-060	CD30	HL, ALCL, T-NHL	No	^102,103^
	Daratumumab	CD38	MM	Yes	^75–78^
	Isatuximab	CD38	MM	Yes	^82–84^
	Dacetuzumab	CD40	MM, NHL, DLBCL	No	^104–106^
	Elotuzumab	CS1 (SLAMF7)	MM	Yes	^79–81^
	Milatuzumab	CD74	MM, MCL, FL, CLL	Yes	^107–110^
	Lintuzumab	CD33	AML	No	^25^
	BI 836858	CD33	AML	No	^111^
bsAbs	Blinatumomab	CD19/CD3	B-ALL, B-NHL, DLBCL	Yes	^124–126^
	AFM11	CD19/CD3	B-ALL	No	^127^
	Mosunetuzumab	CD20/CD3	FL	Yes	^131^
	Glofitamab	CD20/CD3	B-NHL, DLBCL	No	^132,133^
	Epcoritamab	CD20/CD3	B-NHL, DLBCL/LBCL, FL	No	^134,135^
	Odronextamab	CD20/CD3	B-NHL, DLBCL	No	^136^
	Plamotamab	CD20/CD3	B-NHL, DLBCL	No	^137,138^
	Teclistamab	BCMA/CD3	MM	Yes	^150–154^
	Linvoseltamab	BCMA/CD3	MM	No	^155,156^
	Elranatamab	BCMA/CD3	MM	No	^157,158^
	Alnuctamab	BCMA/CD3	MM	No	^159^
	AMG420	BCMA/CD3	MM	No	^162^
	TNB-383B	BCMA/CD3	MM	No	^163^
	AMG701	BCMA/CD3	MM	No	^164^
	PF-06863135	BCMA/CD3	MM	No	^165^
	Bi38-3	CD38/CD3	MM	No	^160^
	AMG424	CD38/CD3	MM	No	^161^
	ISB-1342	CD38/CD3	MM	No	^166^
	GBR-1342	CD38/CD3	MM	No	^167^
	Talquetamab	GPRC5D/CD3	MM	No	^168^
	Cevostamab	FcRH5/CD3	MM	No	^169^
	Flotetuzumab	CD123/CD3	AML/MDS	No	^142,146^
	XmAb14045	CD123/CD3	AML	No	^147^
	AMG330	CD33/CD3	AML	No	^143^
	AMV564	CD33/CD3	AML/MDS	No	^144^
	JNJ-63709178	CD33/CD3	AML	No	^145^
	MCLA117	CLEC12A/CD3	AML	No	^148^
	ESK1-BiTE	WT1/CD3	AML	No	^149^
	AFM26	BCMA/CD16A	MM	No	^175^
	TandAb	CD30/CD16A	HL	No	^176^
	CS1-NKG2D biAb	CS1/NKG2D	MM	No	^178^
tsAbs	TsAb	CD19/CD22/CD3	B-ALL	No	^177^
	161533 TriKE	CD16/IL-15/CD33	AML	No	^179^
CiTE	CiTE	PD-L1/CD33/CD3	AML	No	^613^
SMITE	SMITE	CD19/CD3 & CD28/PD-L1	CD19-positive lymphoma or leukemia	No	^614^
ADC	Inotuzumab ozogamicin	CD22	B-NHL, B-ALL	Yes	^187,188^
	Moxetumomab pasudotox	CD22	HCL, B-ALL	Yes	^189,190^
	Pinatuzumab vedotin	CD22	DLBCL, FL	No	^191^
	BL22	CD22	B-ALL, HL	No	^192,193^
	Polatuzumab vedotin	CD79b	B-NHL, DLBCL, FL	Yes	^191,209,210^
	Loncastuximab tesirine	CD19	B-NHL, DLBCL	Yes	^203,204^
	Coltuximab ravtansine	CD19	B-ALL, B-NHL	No	^205,206^
	Denintuzumab mafodotin	CD19	B-ALL	No	^207^
	Combotox	CD19 and CD22	B-ALL	No	^208^
	Naratuximab emtansine	CD37	B-NHL	No	^213^
	AGS67E	CD37	B-NHL, T-NHL, CLL, AML	No	^214,215^
	Brentuximab vedotin	CD30	cHL, PTCL, ALCL, CTCL	Yes	^181,194–197^
	Camidanlumab tesirine	CD25	cHL	No	^221^
	Belantamab mafodotin	BCMA	MM	Yes	^211^
	HDP-101	BCMA	MM	No	^212^
	Indatuximab ravtansine	CD138	MM	No	^216^
	Lorvotuzumab mertansine	CD56	MM	No	^217^
	Milatuzumab doxorubicin	CD74	MM	No	^218^
	LM-305	GPRC5D	MM	No	^219^
	Gemtuzumab ozogamicin	CD33	AML	Yes	^198^
	Vadastuximab talirine	CD33	AML	No	^199,200^
	IMGN779	CD33	AML	No	^201,202^
	Pivekimab sunirine	CD123	AML	No	^220^

*mAbs* monoclonal antibodies, *bsAbs* bispecific antibodies, *tsAb* trispecific antibodies, *CiTE* bifunctional checkpoint inhibitory T cell–engager, *SMITE* Simultaneous multiple interaction bispecific T-cell engager, *ADC* antibody-drug conjugate, *FDA* Food and Drug Administration, *B-NHL* B-cell non-Hodgkin lymphoma, *DLBCL* diffused large B-cell lymphoma, *FL* follicular lymphoma, *MCL* mantle cell lymphoma, *CLL* chronic lymphocytic leukemia, *cHL* classical Hodgkin lymphoma, *ALCL* anaplastic large cell lymphoma, *PTCL* peripheral T-cell lymphoma, *MM* multiple myeloma, *AML* acute myelocytic leukemia, *B-ALL* B-cell acute lymphoblastic leukemia, *MDS* myelodysplastic syndromes, *HCL* hairy cell leukemia, *CTCL* cutaneous T-cell lymphoma, *BCMA* B cell maturation antigen, *GPRC5D* G protein-coupled receptor, *FcRH5* Fc receptor homolog 5, *CLEC12A* C-type lectin domain family 12 member A, *NKG2D* natural killer cell group 2 member D

### bsAbs

In complex disease pathogenesis, multiple mediators facilitate the stimulation of different signaling pathways or promote overlapping signaling cascades, which limits the therapeutic efficacy of the targeting of a single molecule.^115^ Therefore, the bsAbs, which combine the binding sites of two mAbs in the same molecule, were developed and transformed into immunotherapy.^116^ The emerging bsAbs, exemplified by BiTEs, which promote the activation and recruitment of CD3 + T cells, have facilitated the fast development of cancer immunotherapy in hematologic malignancies.^117–120^ Similar as mAbs, the targeted antigens of bsAbs must be selected from tumor-associated antigens (TAAs) with high specificity and high correlation with the malignant phenotype of the tumor.^120,121^ The bsAbs are mainly divided into three categories according to their targets: (i) antibodies that target two different tumor antigens; (ii) antibodies that target one tumor antigen and one immune-related molecule, such as CD3 for BiTE; and (iii) antibodies that target two immune-related molecules.^117^ Because the BiTE molecule usually targets one CD3 molecule and one tumor antigen simultaneously, it belongs to the second category of bsAbs (Fig. 2b).^117^ BiTEs are the main patterns by which bsAbs work in hematologic malignancies, such as blinatumomab (anti-CD19/CD3 bsAb) approved by the FDA for R/R ALL,^26,122–126^ AFM11 for B-ALL,^127^ anti-CD19/CD3 or anti-CD20/CD3 bsAbs for B-NHL,^128–138^ anti-CD33/CD3, CD123/CD3, WT1/CD3, or CLEC12A (C-type lectin domain family 12 member A)/CD3 bsAbs for AML or MDS,^139–149^ and anti-BCMA (B cell maturation antigen)/CD3 or CD38/CD3 bsAbs for MM.^150–167^ In addition, the anti-GPRC5D (G protein-coupled receptor, family C, group 5, member D)/CD3 and anti-FcRH5 (Fc receptor homolog 5)/CD3 bsAbs were also used for the treatment of MM.^168,169^ Table 1 presents the bsAbs currently developed for hematologic malignancies. Although BiTEs have been proven to be efficient in many R/R hematologic malignancies, several patients still show no responsiveness to BiTE therapy. It is not only due to defects in the structure itself but also the immune escape, involving the aspects of loss of target antigen expression, disrupted trafficking of the target antigens and extramedullary lesions.^170–174^ Based on this fact, bsAbs and trispecific antibodies (tsAbs) engaging NK cells have also been explored in pre-clinical and/or clinical studies.^175–179^ Ross et al. reported the NK-cell mediated lysis of BCMA-positive MM cell lines induced by AFM26 (anti-BCMA/CD16A bsAb).^175^ Moreover, the anti-CD19/CD22/CD3 tsAb that site-specifically fuses anti-CD19 scFv (single chain variable fragment) and anti-CD22 nanobody to CD3 antigen-binding fragment, was designed for treating patients with B-ALL.^177^ It demonstrated enhanced anti-tumor efficacy and the capacity to overcome immune evasion when compared with the corresponding bsAbs alone or multiple antibodies in combination.^177^ The therapeutic effects provide a new direction for the development of bispecific and even multi-specific antibodies.

### ADCs

The mAbs have the advantage of a longer plasma half-life, yet they are not inherently cytotoxic. In contrast, small molecule cytotoxic agents commonly utilized in chemotherapy have high cytotoxicity and relatively low costs of production, but they are poorly targeted to cancer cells and have a plasma half-life of only a few hours.^180–182^ The concept of utilizing the specific binding properties of mAbs as a mechanism to selectively deliver cytotoxic agents to tumor cells is an appealing approach to overcome the challenges of increasing the therapeutic potentials of cytotoxic agents. All three components of an ADC, the antibody, cytotoxic payload, and the linker chemistry that joins them together, are important for the design of an effective anticancer agent. Mechanistically, ADC differs from the previously mentioned mAb and bsAb in that after it binds to the tumor surface antigen, the antigen undergoes endocytosis and ADC will be internalized into the tumor cell and subsequently transported to the lysosome to release the cytotoxic payload (Fig. 2c). The released toxic payload can induce apoptosis and kill surrounding cancer cells through bystander effects (Fig. 2c).^183^ Perhaps the most essential aspect of developing an effective molecule is the selection of the targeted antigen to which the ADC will bind.^184–186^ Advances in related technology, improvements in the selection of cytotoxic agents and the use of smaller conjugates have all dramatically enhanced the potential clinical benefits of ADCs. Several ADCs have been designed and used for clinical use in hematologic malignancies and their targets include CD22,^187–193^ CD30,^181,194–197^ CD33,^198–202^ CD19,^203–208^ CD79,^191,209,210^ BCMA,^211,212^ CD37,^213–215^ CD138,^216^ CD56,^217^ CD74,^218^ GPRC5D,^219^ CD123,^220^ and CD25,^221^ (Table 1). The initial excitement for ADCs has risen and then fallen with the approval and subsequent withdrawal of gemtuzumab ozogamicin in the years 2000 and 2010, respectively.^20^ With effectiveness in the treatment of R/R HL and SALCL, brentuximab vedotin, an anti-CD30 antibody linked to a microtubule inhibitor monomethyl auristatin E (MMAE), received FDA approval for cancer treatment in 2011 and for post-autologous HSCT consolidation in 2015.^196,222^ Inotuzumab ozogamicin is comprised of a humanized anti-CD22 mAb conjugated to calicheamicin, a cytotoxic antibiotic agent and was as monotherapy for the treatment of CD22-positive B-ALL in 2017.^180,223,224^ Vadastuximab talirine (SGN-CD33A, 33A), a novel ADC consisting of pyrrolobenzodiazepine dimers linked to a mAb targeting CD33, has demonstrated activity and a tolerable safety profile as a single agent in patients with AML.^199^ Belantamab mafodotin^225^ targeting BCMA is currently the only ADC approved by the FDA for MM. Furthermore, other TAAs expressed highly on MM cells are also designed as targets of ADCs. Clinical trials of lorvotuzumab mertansine (anti-CD56 ADC),^217^ indatuximab ravtansine (anti-CD138 ADC),^216^ milatuzumab doxorubicin (anti-CD74 ADC),^218^ and the first anti-GPRC5D ADC, LM-305,^219^ are ongoing in present.

### ICIs

Although more ICIs have been developed already,^226,227^ anti-CTLA-4 (ipilimumab), PD-1 (pembrolizumab, nivolumab, pidilizumab) and PD-L1 antibodies (atezolizumab, avelumab and durvalumab) have been the focus of current clinical consideration of checkpoint inhibitors.^32^ PD-1 is a prominent immunosuppressive trans-membrane molecule that is expressed on the surface of T cells.^228^ In the tumor microenvironment (TME), T cells express high levels of PD-1 molecules, which can bind to PD-L1 on tumor cells or other immune cells and PD-L2 on macrophages and dendritic cells (DCs). This will inhibit the intracellular signaling transduction of T cells, reduce effector T cell activity, induce T cell apoptosis, negatively regulate the anti-tumor immune response and ultimately cause tumor cells to undergo immune escape.^229–233^ In addition to surface PD-L1 molecule, tumors can also secrete soluble PD-L1, which more readily binds to PD-1 on T cells.^234,235^ Furthermore, immune cells in TME sometimes are accomplices as well. Despite the direct suppression of T cells, Treg-expressed CTLA-4 can deplete CD80/CD86 by trogocytosis to release free PD-L1 on antigen-presenting cells.^236^ Presence of PD-L1-expressing DCs and macrophages in TME may play a dominant role in mediating T-cell immunosuppression.^234^ The use of mAbs or inhibitors targeting PD-1 or its ligands PD-L1 and PD-L2 can selectively block PD-1 and ligand binding between tumor cells and T cells, thereby helping to restore the anti-tumor immunity of the body and simultaneously enhance the lysis effect of cytotoxic T cells to achieve the effect of tumor eradication (Fig. 2d).^237^ Once the “Cancer-Immunity Cycle” is established, it can produce long-lasting anti-tumor effects. PD-1 inhibitors also enhance the efficacy through the activation of other immune cells within the TME.^238^ A robust anti-tumor T-cell response is induced in tumor-draining lymph nodes by blocking PD-L1-mediated inhibition of host antigen-presenting cells (APCs) at off-tumor sites.^239^ A further opinion has been recently expressed that the activity of ICI is not limited to TME. PD-1 blockade drives the expansion of a subset of PD-1^low^CD8+ progenitor cells with self-renewal properties, resulting in the mobilization of stem-like precursor CD8 + T cells that reside outside the tumor.^240^ CTLA-4 molecule is normally expressed on the surface of CD4+ and CD8 + T cells and can bind with high affinity to B7 ligands on APCs, producing signals that inhibit T cell activation, reduce cytokine production and decrease the anti-tumor immune response.^241,242^ CTLA-4 inhibitors block the co-stimulatory signal between CTLA-4 molecule and Fc on the surface of regulatory T cells, which can induce regulatory T cell death; in addition, this can also block the binding between CTLA-4 molecule and B7 during T cell activation, increase the level of T cell recognition to TAAs and enhance the anti-tumor responses of the immune cells (Fig. 2d).^243–245^ T cell dysfunction, the metabolic profile of CD8 + T cells and immunosuppressive factors lead to resistance of ICIs.^246–249^ The use of PD-1 blockade can also induce anti-PD-1 resistance by induction of dysfunctional PD-1+CD38^high^CD8+ cells.^250^ Therefore, combination therapy of ICIs is emerging in the treatment of hematologic malignancies.^251–253^

### ACTs

ACT is a kind of immunotherapy in which autologous or allogeneic immune effector cells, activated and expanded in vitro, are infused into the patient. Such therapies are divided into non-specific and specific cellular therapies. Non-specific cellular therapy includes the direct infusion of cytokine-induced killer (CIK) cells, tumor-infiltrating lymphocytes (TIL), γ/δ T cells and NK cells, some of which have been used for hematologic malignancies.^254–258^ The mechanism by which non-specific cellular therapy alleviates tumor symptoms is to boost the immunity of the entire body, leading to limited efficacy. Therefore, specific cellular therapies, particularly CAR-T cells, have become more popular in clinical studies.^259,260^ An incredible area of immunotherapy for hematologic malignancies is the development and refinement of CAR-T cell therapy. Such therapies involve not only targeting tumor antigens but also augmenting these targeted immune effectors. CAR-T cells are designed to express CAR that aims to target specific tumor surface antigens with antigen specificity and HLA independence and is therefore not dependent on MHC (major histocompatibility complex) expression. CAR-NK cells, not only recognize tumor antigens specifically via the CAR but also eliminate tumors by the NK cell receptor itself. NK cell activity depends on the balance of stimulatory and inhibitory signals and is antigen non-specific. Targeted lysis of CAR-NK cells is based on CAR-dependent and NK receptor-dependent mechanisms and this lysis effect is also indicated for antigen-negative cancer cells.^34,261,262^ The main sources of CAR-NK cells are usually peripheral blood, cord blood, induced pluripotent stem cells (iPSCs) and NK92 cell lines. Since CAR-T therapy has the largest number of clinical trials and the widest range of applications in the field of cell therapy in hematologic malignancies, especially for multi-line therapy-refractory patients, we will focus on CAR-T therapy in the following sections.

**Fig. 3:**
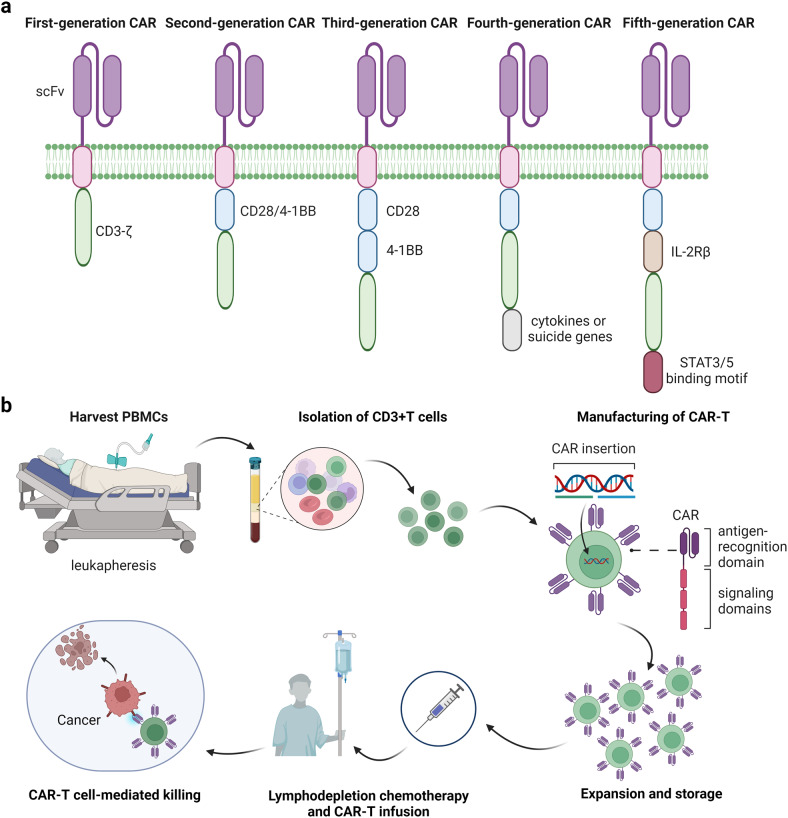
The evolution of CAR design and the process of CAR-T therapy in clinic. **a** The design of the CAR has undergone several updates throughout the evolution of CAR-T therapy. To date, there have been five generations of CAR structures. **b** CAR-T cell therapy is a multi-step process that involves selecting eligible patients, collecting cells, manufacturing CAR-T cells, lymphodepletion and infusion of CAR-T cells and subsequent longitudinal follow-up

The design of the CAR is the pivotal issue and has undergone several updates throughout the evolution of CAR-T therapy (Fig. 3a). Eshhar et al. were the first to construct the CAR-T cells for the expression of antigen receptors.^263^ The intracellular structural domain of first-generation CAR-T cells contains only the signal transduction structural domain CD3-ζ, so that CAR-T cells have poor proliferative abilities and a short survival time in vivo, due to the absence of co-stimulatory signaling and cytokine signaling, such as interleukin-2 (IL-2).^264,265^ The co-stimulatory structural domain CD28 or 4-1BB (also known as CD137) were integrated with the CD3-ζ molecule in the design of the second-generation CAR-T cells, which allowed CAR-T cells to continuously proliferate and induce enhanced anti-tumor activity.^266,267^ The second-generation CAR-T cells, which are the most widely used in clinical practice, were able to exert anti-tumor effects even in the absence of exogenous costimulatory molecules.^268^ Two different costimulatory domains (CD28/4-1BB or ICOS/4-1BB) are present in third-generation CAR-T cells.^269–271^ The fourth-generation CAR-T cells incorporate cytokines or co-stimulatory ligands to further enhance T-cell responses, or suicide genes to enable CAR-T cells to self-destruct when needed.^272,273^ The fifth-generation CAR-T cell is also derived from the second-generation and includes a shortened cytoplasmic IL-2 receptor β chain domain (IL-2Rβ) and a STAT3 binding moiety.^274^ This design enables the fifth-generation CAR-T cells to enhance the T cell receptor (TCR) and cytokine-driven JAK-STAT signaling pathways to promote the proliferation and activation of the bioengineered T cells.^274^ In addition to improvements through co-stimulatory domains and cytokines, more important is the design of the antigen-binding region scFv of CARs. The earliest scFv targeting CD19 was also of murine origin (FMC63) and it would generate murine-derived mAbs, namely anti-CAR immune responses. Moreover, this response has also been shown to affect CAR-T efficacy and even lead to late relapse.^275,276^ Therefore, researchers are continuously working on humanizing scFv fragments and directly design fully human CAR fragments to reduce the occurrence of this response and its impact on efficacy.^277–279^ Besides, more novel types of CAR-T cells are being developed to improve the flexibility of CAR target recognition. To address the problem of wait for a long time, the “off-the-shelf” CAR-T cells, in which all T cells are derived from healthy donors, have been developed.^280,281^ The universal CAR-T cells replace scFv extracellular structural domain used in previous generations of CAR T cells with an adapter-specific recognition structural domain which binds to an adaptor molecule specific to a tumor target. This design enables CAR-T cells to recognize multiple antigens by separating the antigen-targeting structural domain from the T-cell signaling unit.

CAR-T cell therapy is a multi-step process that involves selecting eligible patients, collecting cells, manufacturing CAR-T cells, lymphodepletion, infusion of CAR-T cells, and subsequent longitudinal follow-up (Fig. 3b). The eligibility of patients depends on their disease status, previous treatment regimens, risk factors, co-morbidities, performance status and social factors.^282^ The patient’s peripheral blood mononuclear cells (PBMCs) are collected by leukapheresis and CD3 + T cells are further purified and isolated. T cell subpopulations are genetically modified to express the CAR of interest, then expanded in vitro. The expanded CAR T cells are frozen and stored for future use and ultimately reinfused into the patients after lymphodepletion-directed chemotherapy. CAR T-cell therapy generally requires hospitalization and the patient’s physical reactions, especially the possibility of AEs, should be closely monitored for several weeks after infusion.

CAR-T cell immunotherapy has gradually become the main therapeutic option for malignant hematological diseases, with impressive results to date. From Kymriah and Yescarta, which were the first to be approved by the FDA for the treatment of leukemia and lymphoma in August and October 2017, respectively, to the latest advances such as CB-010 therapy, they all play a pivotal role in treating malignancies, especially in cases of R/R patients. CAR-T cell immunotherapy has already achieved notable successes in the treatment of B-cell malignancies such as ALL, CLL, and DLBCL. Meanwhile, the most commonly utilized CAR targets for B-cell malignancies are CD19, CD20, and CD22.^283^ Of these, CD19 is the most commonly used target and is highly expressed in the majority of B-cell malignancies. CD7 is an important target in T-cell ALL and T-cell lymphoma.^284–286^ CD30 is usually expressed on tumor cells of HL,^287^ and CD33 is a favorable target for AML.^288^ Two CAR T-cell products, idecabtagene vicleucel and ciltacabtagene autoleucel, are the currently FDA-approved BCMA-targeting therapies. In addition to BCMA, many other investigational CAR T-cell therapies for MM are being studied, including cell products targeting SLAMF7, CD19, CD38, TACI (transmembrane activator and CAML interactor), GPRC5D (G protein-coupled receptor, class C, group 5, member D), and CD138.^282,289–291^ However, the application of CAR-T therapy has been limited by relapse, resistance and toxicity.^292–298^ Researchers have used diverse approaches to improve CAR-T therapy. In terms of target selection, new targets have been diligently searched for,^299,300^ and even dual-target and even multi-antigen-targeted CAR-T have been introduced^291,301–306^ to prevent subpopulations of tumors from being ignored.^307^ For T-ALL, patients’ own T cells are difficult to make CAR-T, thus healthy donor T cells are used to prepare CAR-T.^281^ Recently CAR-NK and CAR-macrophage cells have also become new popular products and novel CARs are designed to overcome treatment failure.^308–310^ Despite these advancements in CAR-T cell therapy, there are still several unanswered questions. For example, the optimal CAR T cell design and engrafting technique, the ideal intracellular costimulatory domain or the generation of CARs, the appropriate CD4:CD8 T cell ratio in infusion products and even factors such as the dominance of effector versus central memory cells and the influence of Tregs are unknown. The best timing for the engraftment of CAR-T cells is also not yet clear and may vary depending on the type of malignancies. In addition, the impact of TME may be an additional critical factor in CAR T-cell therapy. Although these questions remain unanswered, CAR T-cell therapy will be an essential strategy for the treatment of hematologic malignancies. As more research is conducted on this breakthrough therapeutic approach, it will be improved in its efficacy and applicability.

### Tumor vaccines

Tumor vaccines, one of the hot topics in research in recent years, are immunotherapeutic modalities in which tumor antigens are infused into patients in various forms to generate tumor-specific lymphocytes in the patient and kill the tumor.^311^ It consists of molecular vaccines and cellular vaccines, among which molecular vaccines include tumor-associated proteins or peptides and gene vaccines expressing tumor antigens. Cellular vaccines, on the other hand, are tumor cells, which are genetically modified to express MHC molecules and then injected into patients. Tumor vaccines can enhance the immunogenicity of the tumor, activate the patient’s immune system, induce the body’s cellular and humoral immune response and also override the immunosuppressive state caused by the tumor. It is designed to not only induce tumor regression, but also to eliminate minimal residual disease (MRD), establish long-lasting anti-tumor memory and avoid non-specific or adverse reactions. Such vaccines have been developed for B-cell leukemia and lymphoma, ranging from commonly-mutated genes to DC vaccines.^312,313^ Vaccines targeting immunoglobulin light chain and EBV antigens are also available.^314,315^ As clinical trials have been conducted,^316–318^ although not yet widely used, the prospects are promising.

## How immunotherapies work: to promote “Cancer-Immunity Cycle”

**Fig. 4:**
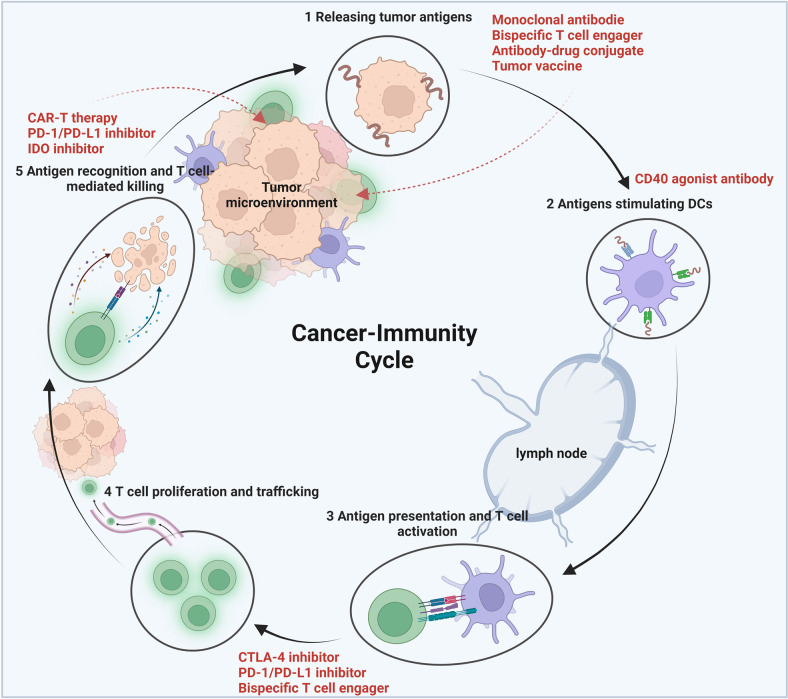
How immunotherapies work? To promote “Cancer-Immunity Cycle”. The “Cancer-Immunity Cycle” can be divided into multiple steps.^6^ Dysregulation of the “Cancer-Immunity Cycle” is the consequence of tumorigenesis and treatment failure. Meanwhile, the TME may also suppress these effector cells engaged in the “Cancer-Immunity Cycle” and resultant cancer immune evasion. Numerous factors that play a part in any step of this cycle offer a wide range of potential therapeutic targets: (i) promoting antigen release, presentation and recognition; (ii) priming and activating the immune response; (iii) overcoming immune evasion; (iv) targeting immune suppression in the TME

The generation of anti-cancer immunity is a cyclical process that can be self-perpetuating, with the accumulated immunostimulatory factors that should, in principle, boost the T cell immune response. This cycle can also be interrupted by suppressive stimuli, which result in immunomodulatory feedback mechanisms that impede the generation of anti-cancer immunity.^6^ Generally, the “Cancer-Immunity Cycle” can be divided into multiple steps. First, the neoantigens that are produced during tumorigenesis are released and then captured by the DCs for processing. This must be accompanied by immune-specific signals so as not to induce peripheral immune tolerance to the tumor antigens. Then, DCs deliver antigens that are captured on MHC molecules to T cells, leading to the priming and activation of effector T cells. Subsequently, through the interaction between the TCR and the cognate antigen bound to MHC-I, these activated effector T cells traffic towards and infiltrate into the tumor, where they specifically recognize and bind to the cancer cells and kill them. Noteworthy, the killing of these targeted cancer cells also leads to the release of more TAAs. This in turn extends the breadth and depth of the immune response in subsequent cycles of rotation.^6^ Dysregulation of the “Cancer-Immunity Cycle” is the consequence of tumorigenesis and treatment failure. Meanwhile, the TME may also suppress these effector cells engaged in the “Cancer-Immunity Cycle” and resultant cancer immune evasion.^6,7^ Therefore, cancer immunotherapy requires initiating and promoting the self-sustainability of the “Cancer-Immunity Cycle” so that it can normally amplify and spread, but not to the point of generating an unrestrained autoimmune inflammatory response. In the meantime, cancer immunotherapy also needs to be carefully tailored to counteract these negative feedback mechanisms.^8–10^ Numerous factors that play a part in any step of the “Cancer-Immunity Cycle” offer a wide range of potential therapeutic targets (Fig. 4): (i) promoting antigen release, presentation and recognition; (ii) priming and activating the immune response; (iii) overcoming immune evasion; (iv) targeting immune suppression in the TME.

### Promote antigen release, presentation and recognition

Although not established as immunotherapies, chemotherapy, radiotherapy and targeted therapies (e.g., mAbs, bsAbs, and ADCs) can kill large numbers of cancer cells, then promote antigen release and T cell activation. The majority of tumor vaccines are therapeutic vaccines, which are based on the principle that tumor antigens are introduced into the patient’s body to improve immunogenicity, activate the immune system and elicit cellular and humoral immune responses to control or eliminate the tumor.^311^ Theoretically, it is feasible to promote the activation of the immune system through the specific proteins of cancer cells so as to eliminate cancer cells. Nevertheless, tumor antigens are heterogeneous thus the primary problem in tumor vaccine development is to find the universal or specific antigens expressed on the surface of tumor cells.^319,320^ CD40 agonist antibodies are used to promote the maturation and antigen-presenting ability of DCs by mimicking CD40L cross-linking CD40, inducing the expansion of tumor antigen-specific cytotoxic T cells and thus eradicating tumors.^105,321,322^ CAR T-cell therapy is the process of transferring genetic material with specific antigen recognition structural domain and T cell activation signal into T cells through genetic modification. In this way, the modified T cells can be activated in an MHC-independent manner by directly binding with specific tumor antigens and directly killing the tumor cells by releasing perforin, granzyme B, etc. and also by secreting cytokines to recruit human endogenous immune cells to help to kill tumor cells.

### Priming and activation of immune response

CTLA-4, an inhibitory receptor that is expressed primarily on T cells, has a suppressive function on T cell activation and is upregulated upon T cell activation. Antibodies targeting the immunomodulatory receptor CTLA-4 have two putative mechanisms of action: direct inhibition of CLTA-4 binding to its cognate ligand and depletion of immunosuppressive regulatory T (Treg) cells via Fc-mediated immune-mediated mechanisms, mainly including ADCC and CDC.^245^ More importantly, the BiTEs are able to redirect T cells to specific tumor antigens and to directly activate the T cells.^323^ Because T cells lack Fcγ receptors, natural antibodies cannot directly recruit these T cells. The BiTE molecule typically targets a tumor antigen and a CD3 molecule at the same time. The CD3 molecule associates non-covalently with the T cell receptor (TCR) and participates in antigen-specific signal transduction that can induce T cell activation. In addition, directly expanding and making available increased numbers of functionally competent immune cells represents an intuitively desirable therapeutic concept.^19^ HSCT refers to the transplantation of hematopoietic stem cells from a donor into a recipient to rebuild or restore the recipient’s immune system. Cellular immunotherapy stimulates the body’s anti-tumor immune response by isolating autologous or allogeneic immune effector cells, activating them in vitro and then injecting them into the body. As with CAR-T cell therapy, the scFv recognizes specific TAAs, including the proteins, glycoproteins and other components. CD3-ζ is typically a signaling region containing three ITAMs (immunoreceptor tyrosine-based activation motifs). Upon scFv recognition and binding to TAA, phosphorylation of the ITAM triggers ZAP70 signal transduction and subsequent signaling to initiate and prime the T cell immune responses.^324^ This is a principle similar to antigen-antibody complementarity, which can bypass the MHC-dependent antigen presentation and enable the TAA to directly stimulate the activation of CAR-T cells.

### Overcoming immune evasion

An important mechanism by which tumor immune evasion occurs is by suppressing the function of effector immune cells. Immune checkpoints are a class of molecules that have a negative effect on immune cell function and are most expressed in immune cells. They can regulate the degree of activation of the immune system, resulting in them playing an important role in the prevention of autoimmune effects. However, these molecules are susceptible to being hijacked by tumor cells, which means the tumor cells can bind to the corresponding ligand/receptor on the immune cell, activating the inhibitory pathway and preventing immune cells from killing the tumor, thus enabling the immune escape of the tumor.^3^ ICIs aim to block the corresponding immune checkpoints to prevent the activation of the relevant immunosuppressive pathways and have been widely used in various types of solid and hematologic malignancies.^325,326^ Moreover, T cell exhaustion occurs due to a multi-factorial etiology resulting from sustained exposure to tumor antigens, the loss of stimulation/secretion of effector cytokines, the involvement of immunosuppressive cell types and immunophenotypic alterations including increased expression of inhibitory receptors and checkpoints such as LAG3 (lymphocyte-activation gene 3), TIGIT (T cell immune receptor with Ig and ITIM domains), TIM3 (T cell immunoglobulin mucin 3). Therefore, T cell exhaustion may be reversed and the anti-tumor immune response enhanced by inhibitors targeting these inhibitory receptors and checkpoints.

### Targeting immune suppression in TME

The TME is the internal environment in which tumor cells survive and develop and immune cells in the TME have different mechanisms of pro- or anti-tumor immune action in tumor growth and progression. Tregs suppress T cell activity either directly or by secreting suppressor cytokines such as IL-10 and TGF-β; myeloid-derived suppressor cells (MDSCs) suppress T cell activity and modulate the intrinsic immune response to suppress the immune response. Therefore, targeting the TME is another important mechanism of cancer immunotherapy. For example, overexpression of indoleamine 2,3-dioxygenase (IDO) in tumors inhibits T cell proliferation and promotes regulatory T cell differentiation and IDO inhibitors can effectively improve the immunosuppressive microenvironment of tumors and enhance the anti-tumor immune response.

## Representative clinical trials and outcomes

### HSCT

Numerous clinical trials have validated the elimination of hematologic malignancies through transplantation.^327–329^ Transplantation-related clinical trials mainly involve two aspects: (i) exploration of peripheral blood stem cell transplantation (PBSCT) and RIST; (ii) comparison of allogeneic HSCT with HLA genotype identical sibling donors (ISD) and haploidentical donors (HID). Around the beginning of the 21st century, several clinical trials were conducted to investigate the efficacy and safety of PBSCT.^330,331^ These results confirmed the advantage of PBSCT in terms of hematopoietic system reconstitution. Meanwhile, it makes HSCT less harmful to the donor. To expand the application, RIST has been raised for those who can’t tolerate allo-HSCT. And relevant clinical trials were designed to discover the appropriate chemotherapy regimen and compared RIST with high-dose conventional conditioning. A 7-year clinical trial showed that 8 out of 12 patients who received RIST were still alive after 1 year, while only 3 out of 13 patients who received high-dose chemotherapy were still alive.^332^ Fludarabine-melphalan as a preparative regimen for RIST is associated with a significant reduction in transplant-related mortality according to an update from the MD Anderson Center.^333^ The study in Europe has also shown a reduction in the non-relapse mortality rate in RIST.^334^ To date, more clinical trials are ongoing to evaluate RIST in elderly patients with AML and MDS.^335–337^ Haplo-HSCT is now being used regularly for patients. However, it was not until 2015 that the technology became more mature and clinical trials comparing it to the ISD-HSCT were conducted.^338,339^ The results of haplo-HSCT performed in patients who were in remission did not differ significantly from those of ISD-HSCT. In later studies, both transplantation methods were applied to patients not in remission, where haplo-HSCT showed better efficacy.^340^ Although there may be a higher rate of GVHD, it has the potential to be used in high-risk child patients.^341^ In addition, haplo-HSCT can be followed by adoptive T-cell therapy and the results of such trials have shown that T-cell infusion can be beneficial in reconstituting the immune system and preventing relapse.^342,343^

### mAbs

The most representative mAb used in the treatment of lymphoma is none other than Rituximab. There is a pivotal phase II trial of rituximab monotherapy that was conducted in 166 patients with R/R low-grade NHL, in which the ORR was 48 and 6% of the patients achieved the complete response.^344^ The stage was set for the approval of rituximab with these and subsequent results.^345^ However, an increasing number of clinical trials have opted to use rituximab in combination with other chemotherapy regimens to improve efficacy. In 2001, one phase II trial of the first-line R-CHOP regimen was initiated in 33 patients with aggressive NHL. The results were surprising with an ORR of 94% and a CRR (complete response rate) of 61%, demonstrating for the first time the feasibility and safety of the R-CHOP regimen in these patients.^86^ Clinical trials of R-CHOP in MCL were then conducted. As implied by the results of a prospective randomized trial conducted by the German Low-Grade Lymphoma Study Group (GLSG), R-CHOP was significantly superior to CHOP as first-line therapy in terms of ORR (94%), CRR (34%) and time to treatment failure (21 months), although no differences were observed in progression-free survival (PFS).^346^ Currently, R-CHOP has been designated as the first-line treatment agent for NHL by the National Comprehensive Cancer Network (NCCN), while there are numerous clinical trials to validate the efficacy of R-CHOP as a treatment to overcome relapse or refractory of NHL.^347^ It is approved in Europe and the United States for use in combination with chemotherapy to treat patients with previously untreated or R/R CLL.^348^ For example, a phase II trial evaluated the efficacy of the addition of rituximab to first-line chemotherapy with fludarabine and cyclophosphamide. And the chemo-immunotherapy group achieved a better clinical outcome, with 65% of patients free of disease progression at 3 years after the randomization.^349^ Venetoclax-rituximab was also proved to be able to be applied in R/R CLL with significantly higher rates of PFS(84.9%) at 2 years.^88^

The development of new mAbs is ongoing and clinical trials are being conducted. Ofatumumab, a fully human mAb, has been used as a single-agent CD20 immunotherapy in R/R CLL and FL in international clinical trials and has been shown to be an active, well-tolerated treatment with significant clinical improvements.^71,90,350^ There are some clinical trials, such as GAUDI, GAUGUIN and GADOLIN, to investigate the efficacy of obinutuzumab (also called GA101) monotherapy and immunochemical combination with it in treating patients with DLBCL, MCL, FL and CLL.^73,92–95^ It has also been used to treat CD20-positive indolent NHL refractory to rituximab. In this study, the median PFS was 25.8 months and OS was also prolonged, demonstrating the clinical benefit of obinutuzumab.^74,351^ As well, tafasitamab (anti-CD19 mAb) is also approved for the treatment of R/R DLBCL and FL as a novel agent.^23,209,210,352,353^ Some mAbs which has already been approved in autoimmune disease, such as alemtuzumab (anti-CD52 mAb) and ublituximab (anti-CD20 mAb), also expanded their indications to hematologic malignancies. In the GENUINE trial, ublituximab plus ibrutinib achieved encouraging efficacy in high-risk CLL and the ORR was 83%.^354^ Alemtuzumab combined with CHOP similarly showed better outcomes with an ORR of 72% and CRR of 60% in the phase 3 trial.^100^ In recent years, mAbs have gradually been introduced into the treatment of other hematologic malignancies. Daratumumab, an anti-CD38 mAb, is initially used as monotherapy in R/R MM. In a phase I-II dose-expansion study, daratumumab was administered to patients who had received a median of four prior therapies, including 76% of patients who had received autologous HSCT. The ORR was 36% in the cohort with a dose of 16 mg/kg and 10% in the cohort with a dose of 8 mg/kg. PFS was 5.6 months and 65% of patients who responded had no disease progression at 12 months.^76^ The results of the SIRIUS trial were similar and both were favorable in terms of safety and exciting efficacy.^77^ Daratumumab was also combined with classical regimens of MM to investigate the efficacy. The phase 3 trial suggested that the ORR was higher in the daratumumab combination group (82.9%) than that in the control group with bortezomib and dexamethasone alone (63.2%).^24^ A similar outcome also occurred in the trial that compared the regimen of lenalidomide and dexamethasone, with an ORR of 92.9%.^75^ Afterwards, daratumumab plus bortezomib, melphalan and prednisone was also considered as a prior-line therapy for untreated MM patients. And the outcome indicated that the addition of daratumumab resulted in a lower risk of disease progression or death.^78^ Another anti-CD38 mAb named isatuximab has improved its effectiveness when combined with classical therapy regimens. Randomized phase 3 trials have been completed for all of thesecombinations.^82–84^ Meanwhile, elotuzumab targeted CS1 on MM cells and also indicated encouraging results in serial clinical trials called ELOQUENT that was conducted in R/R and newly diagnosed MM patients.^80,81^ In a word, mAbs occupy an important position in hematologic cancers and chemoimmunotherapy associated with mAbs has become a popular trend at the present.

### bsAbs

In hematologic malignancies, bsAb therapy usually refers to the BiTEs. Blinatumomab is the first bsAb designed for this field. Some early clinical trials were conducted for NHL in the year 2008. Out of 38 patients who received blinatumomab, a response was only observed in 11 patients. And the longest duration of CR is 13 months in one MCL patient.^355^ Furthermore, it has been studied in more cases of B-ALL. A phase II trial has demonstrated that blinatumomab is effective in MRD-positive B-ALL patients who are resistant to previous chemotherapy. The drug showed a high response rate, with an ORR of 76% and a relapse-free survival (RFS) rate of 78%.^356^ Other studies showed similar results,^26,357,358^ and blinatumomab is also effective in children and young adults with the first relapse of B-ALL.^359,360^ Therefore, it has already become an approved therapy for R/R B-ALL. Recently, there’re emerging trials to discover the efficacy of the combination therapies of blinatumomab and other regimens for newly diagnosed Philadelphia chromosome (Ph) positive or negative B-ALL.^124,361–363^ Blinatumomab was also used to treat patients with R/R B-NHL and DLBCL and showed great anti-tumor efficacy.^125,126,364,365^ Meanwhile, some novel bsAbs entered the market in 2022, representing the rapid development of this field. Mosunetuzumab, a CD20/CD3 bispecific antibody, was approved for R/R FL based on the results of the multicenter phase II study in which 90 patients with FL received mosunetuzumab and the ultimate CRR was 60%.^131^ Glofitamab is also targeted to CD20 but has been shown to induce durable CR in patients with R/R DLBCL.^132,133^ In the phase I/II study, 52 patients who had previously received CAR-T therapy were enrolled and 35% of them achieved a CR and 78% of CR were sustained at 12 months.^133^ Epcoritamab, odronextamab and plamotamab are all anti-CD20/CD3 antibodies and relevant clinical trials have demonstrated that they are competitive in terms of efficacy and safety.^134,136^ A anti-BCMA/CD3 bsAb, teclistamab, was the first BiTE developed for MM.^150–154^ In the trial MajesTEC-1, teclistamab demonstrated promising efficacy, with durable responses that deepened over time and was well tolerated in R/R MM patients.^153^ Another phase 1–2 study also showed that teclistamab resulted a high rate of deep and durable response in patients with triple-class-exposed R/R MM.^151^ The ORR was 63%, the median duration of response was 18.4 months and the median duration of PFS was 11.3 months.^151^ Patients enrolled in the trial MajesTEC-2 had received ≥1 prior line of therapy.^152^ While in the trial s MajesTEC-4 and MajesTEC-7, newly-diagnosed patients were enrolled and teclistamab was combined with classical regimens for treating MM.^150,154^ In addition to teclistamab, other anti-BCMA/CD3 bsAbs have emerged currently, including linvoseltamab, elranatamab and alnuctamab and serial trials are being conducted.^155–159,366–368^ In a Phase 2 study, 232 patients received talquetamab (anti- GPRC5D/CD3 bsAb) monotherapy and 70% of those experienced a response and the median duration of response was 10.2 months.^168^ A multicenter, open-label, phase 1/2 study of flotetuzumab (MGD006, anti-CD123/CD3 bsAb) was conducted in 88 adults with R/R AML and showed acceptable safety and encouraging evidence of activity in PIF (primary induction failure)/ER (early relapse) patients.^142^ JNJ-63709178, another kind of anti-CD123/CD3 bsAb, was found to have limited exposures and clinical activity with an unfavorable safety profile.^145^

### ADCs

Over the past several decades, ADCs have been evaluated in many preclinical models and early-phase clinical trials of hematologic malignancies. Gemtuzumab ozogamicin, an anti-CD33 ADC, was once used in AML patients with their first relapse and no history of an antecedent hematologic disorder and a median age of 61 years. This was based on the result of the clinical trial which revealed that 30% of patients who were treated with gemtuzumab ozogamicin achieved a remission, characterized by 5% or fewer blasts in the bone marrow.^369^ However, a phase III SWOG S0106 randomized comparative trial did not confirm the clinical benefit of gemtuzumab ozogamic in combination therapy, such as CR rate, disease-free survival (DFS) and OS. Moreover, increased toxicity was observed and probably caused by relatively high instability of the linker in the bloodstream combined with a high recommended dose.^370^ Thus, gemtuzumab ozogamicin was withdrawn in 2010 due to its serious toxicities and poor outcomes of survival.^371–373^ It has been re-approved until 2017, following adjustments to the dosage and conditions as well as extensive clinical trials.^374–377^ At present, it is believed that the benefit of gemtuzumab ozogamicin can be predicted by some related conditions and this is the reason why gemtuzumab ozogamicin is used in AML with high CD33 expression levels and corresponding mutated genetic profiles (e.g. NPM-1 mutated, KMT2A rearranged).^198,378,379^ Furthermore, gemtuzumab ozogamicin is effective when used in newly diagnosed core binding factor (CBF)-deficient AML in the clinical trial conducted by MD Anderson.^380^ In addition, a humanized anti-CD22 ADC called inotuzumab ozogamicin was initially given to patients with R/R B-NHL in a phase 1 clinical trial. Unfortunately, the final ORR was only 39% for the 79 patients enrolled.^187^ Later on, inotuzumab ozogamicin has been tried to be used in R/R B-ALL patients. In the phase 2 trial, the ORR was 57% for the 49 patients in the study.^381^ To further demonstrate the promise of inotuzumab ozogamicin, it was compared to standard intensive chemotherapy for ALL in a phase 3 trial. In the inotuzumab ozogamicin group, the CR rate was significantly higher (80.7%), the median duration of remission was longer (4.6 months) and the median PFS was also longer (5.0 months).^180^ Based on these results, the FDA approved the use of inotuzumab ozogamicin in adult R/R B-ALL. Meanwhile, clinical trials continued to evaluate the efficacy of the combination therapy in Ph(-) ALL and in pediatric patients.^188,382,383^ Another anti-CD22 ADC, called moxetumomab pasudotox, has been developed for the treatment of R/R hairy cell leukemia (HCL).^189^ In the long-term follow-up from the pivotal trial, complete responders lasting ≥60 months was 61% and median PFS without the loss of hematologic remission was 71.7 months. Moxetumomab pasudotox fills the gap in R/R HCL where there is no adequate therapy.^384^ In 2022, brentuximab vedotin (anti-CD30 ADC) was used in patients with III/IV-stage cHL. Compared with the classical ABVD (doxorubicin, bleomycin, vinblastine and dacarbazine) regimen, the combination of brentuximab vedotin plus BVD (bleomycin, vinblastine and dacarbazine) showed better consequences with a 6-year OS of 93.9%.^181^ Polatuzumab vedotin has been designed to target CD79b and used for the treatment of R/R B-NHL including DLBCL and FL.^191,209,210^ Polatuzumab vedotin combined with bendamustine and rituximab resulted in a significantly higher CR rate and reduced the risk of death by 58% compared with bendamustine and rituximab in patients with transplantation-ineligible R/R DLBCL.^210^ Loncastuximab tesirine (ADCT-402) is a humanized anti-CD19 IgG1 mAb conjugated through a protease-cleavable Val-Ala linker to a pyrrolobenzodiazepines dimer, a DNA crosslinking agent.^203,204^ A phase 1 study of loncastuximab tesirine in R/R B-cell NHL showed that ORR in evaluable patients was 45.6%, including 26.7% CRs. ORRs in patients with DLBCL, MCL, and FL were 42.3%, 46.7%, and 78.6%, respectively.^185^ Further, a multicentre, open-label, single-arm, phase 2 trial (LOTIS-2) was conducted in patients with R/R DLBCL after two or more multiagent systemic treatments with an ORR of 48.3% and a CRR of 24.1%.^203^ Belantamab mafodotin chose BCMA as the target and fills the gap of ADC in MM and the serial trials continue to discover its clinical efficacy and durability as monotherapy or combined with other regimens.^211,385,386^ The DREAMM-2, a two-arm, randomized, open-label, phase 2 study, demonstrated that 31% of 97 patients in the 2·5 mg/kg cohort and 34 of 99 patients in the 3–4 mg/kg cohort achieved an overall response.^211^ In DREAMM-6 trial, belantamab mafodotin showed a better outcome with an ORR of 75% and a median PFS of 8.6 months.^386^ It seems that ADCs have already played an important role and became a new trend in immunotherapy for hematologic malignancies nowadays. These ADC drugs have achieved satisfactory results in clinical trials and have been approved for use in the diseases for which they are intended.^191,203,204,210,353^ Furthermore, there’re still some novel ADCs waiting for approval and the corresponding clinical trials are ongoing.^199,200,221^

### ICIs

Several clinical trials of ICIs have been conducted in hematologic malignancies, including MM, ALL, AML, NHL and HL.^387–390^ However, only the results of PD-1 blockade in HL are particularly remarkable. Some observations may suggest why HL is uniquely sensitive to PD-1/PD-L1 blockade.^391^ First, HL biopsies typically show the Reed-Sternberg (R-S) cells that are surrounded by an extensive immune infiltration, but it is ineffective. Moreover, increased surface expression of PD-L1 was also observed in HL biopsies. Second, HL is characterized by the genetic alterations in 9p24.1 that result in copy gain and overexpression of PD-L1 and PD-L2, with an increase in copy gain or amplification of 9p24.1 in more than 97% of newly diagnosed HL biopsy specimens.^392,393^ Third, infection with Epstein-Barr virus (EBV) is common in HL patients and also causes PD-L1 to be overexpressed, which is one of the key mechanisms by which the virus could persist in the host.^394^ In contrast, NHL does not display a high frequency of 9p24.1 alterations, thus the efficacy of ICI decreased for NHL patients.^395^

Table 2 gives a summary of representative clinical trials of ICIs that are already approved by the FDA or some novel ICIs (e.g., dual-target ICI) that are still in the stage of the clinical study.^387,396–407^ Ipilimumab, a CTLA-4 inhibitor, has been evaluated in clinical trials of the treatment for NHL and HL patients,^396–398^ but only showed certain therapeutic effects in HL with an ORR of 76% and CRR of 57%.^398^ Since HL has the property of being more sensitive to ICIs targeting PD-1, most of the clinical trials of PD-1 blockades, including nivolumab, pembrolizumab and pidilizumab, were conducted on R/R HL.^194,387,396,398,400,408^ In recent years, nivolumab and pembrolizumab have been used in patients with NHL, CLL^404,409,410^ and even in some lymphomas for which there is no effective therapy, such as PCNSL (primary central nervous system lymphoma) and PMBCL (primary mediastinal large B-cell lymphoma).^402,403,411^ Moreover, there’s a clinical trial of pidilizumab conducted in advanced hematologic malignancies including MM, promoting the wide application of ICIs.^412^ In addition to PD-1 blockade, CD47 blockade has emerged as the treatment for R/R NHL, MM and especially for AML/MDS, where PD-1 blockade shows poor efficacy.^413–417^ To improve the overall response, one phase 1b trial explored the safety and efficacy of combined PD-1 and CTLA-4 blockade in patients with R/R lymphoid malignancies, including HL, NHL, and MM.^399^ But it is regrettable that there was no meaningful improvement in the efficacy of the combinations over single-agent nivolumab in the diseases studied. While this combination was active in HL (ORR 74%, CRR 23%), the toxicity of nivolumab /ipilimumab was higher than expected from nivolumab alone.

**Table 2:** Representative clinical trials and outcomes of ICIs for treating hematologic malignancies

Drug & Target	Trial	Phase	Monotherapy/combination therapy	Type of disease	Prior lines of treatment	No. of patients	Response	Survival	FDA approval	Refs.
Ipilimumab, CTLA-4	NCT00060372	I	Monotherapy	Pan-cancers, including R/R cHL	Received allo-HSCT	29	Only 3 patients demonstrated objective disease responses after ipilimumab alone, 1 patient achieved PR and 2 patients achieved CR	Median OS of all 29 patients was 24.7 months	Yes	^396^
	NCT00089076	I	Monotherapy	R/R B-NHL	1–4 chemotherapy regimens	18	Only 2 patients had clinical response: 1 patient achieved PR and 1 patient achieved CR	1 patient with FL had PR lasting 19 months and 1 patient with DLBCL had an ongoing CR ( > 31 months)	Yes	^397^
	NCT01896999	I/II	Combination therapy	R/R cHL	≥1	61	ORR 76% CRR 57%	Median PFS 1.2 years	Yes	^398^
Ipilimumab and Nivolumab, CTLA-4 and PD-1	NCT01592370	I	Combination therapy	R/R cHL, NHL, and MM	≥1 or ≥2	65	cHL: ORR 74%, CRR 23% NHL: ORR 19%, CRR 6% MM: ORR 0%	cHL: median PFS was not reached at 17 months all corhorts: median PFS was 1–2 months	Yes	^399^
Nivolumab, PD-1	NCT01592370	I	Monotherapy	R/R cHL	≥1 without ASCT in 100 days	23	ORR 87% CRR 17%	PFS: 86% at 24 weeks; OS: 91% at 1 year	Yes	^387^
	NCT03016871	II	Comparison of monotherapy and combination therapy	R/R cHL	/	43	ORR 93% CRR 91%	PFS 72% at 2 years	Yes	^400^
	ADVL1412	I/II	Monotherapy	Pan-cancers, including NHL and HL	≥1	HL 10, NHL 10	HL: ORR 30% NHL: ORR 10%	HL: the median of cycles the patients completed was 4.5; NHL: one patient had response and remained on therapy for 11 cycles.	Yes	^401^
	/	/	Monotherapy	R/R PCNSL and PTL with CNS relapse	≥1	5	ORR 100% CRR 80%	PFS: 60% at 17 months	Yes	^402^
	CheckMate 436	I/II	Combination therapy	R/R PMBCL	≥2 or received ASCT	30	ORR 73% CRR 37%	Median PFS and OS were not reached	Yes	^403^
	NCT02038933	II	Monotherapy	R/R DLBCL	Experienced failure of ASCT	121	Allo-HSCT-failed: ORR 10% at 9 months, ASCT-ineligible: ORR 3% at 6 months	Allo-HSCT-failed: median PFS 1.9 months, median OS 12.2 months; allo-HSCT-ineligible: median PFS 1.4 months, OS 5.8 months	Yes	^404^
	NCT02329847	I/II	Combination therapy	R/R FL/DLBCL/CLL and SLL/RT	≥1	144	CLL/SLL: ORR 61% FL: ORR 33% DLBCL: ORR 36% RT: ORR 65%	CLL/SLL: median duration of response 19.2 months FL: median PFS 9.1 months DLBCL: median PFS 2.6 months RT: median PFS 5.0 months	Yes	^409^
Pembrolizumab, PD-1	KEYNOTE-013	I	Monotherapy	R/R cHL	Received BV without ASCT	21	ORR 65% CRR 16%	PFS 46% at 52 weeks	Yes	^408^
	KEYNOTE-013	I	Monotherapy	R/R PMBCL	Median 3	18	ORR 41%	Median OS was not reached	Yes	^411^
	NCT02453594	II	Monotherapy	R/R cHL	Received BV and/or HSCT	210	ORR 69% CRR 22.4%	PFS 72.4%, OS 99.5% at 6 months	Yes	^405^
	KEYNOTE-204	III	Monotherapy	R/R cHL	≥1 without ASCT	151	ORR 65.6%, CRR 24.5%	Median PFS was 13.2 months	Yes	^194^
	NCT02332980	II	Monotherapy	R/R CLL, RT	≥1	16	RT: ORR 44% CLL: ORR 0%	RT cohort: median OS 10.7 months	Yes	^410^
Pidilizumab (CT-011), PD-1	NCT00532259	II	Monotherapy	R/R DLBCL	Received ASCT	66	ORR 51%	PFS 72%, OS 85% at 16 months	Yes	^390^
	NCT00904722	II	Combination therapy	R/R FL	≥1	32	ORR 62%, CRR 52%	Median PFS was 18.8 months	Yes	^406^
	CT-011 Trial	I	Monotherapy	Advanced NHL, HL, CLL, MM	/	7	B-NHL: ORR 14%, CRR 14%	The survival time ranged from 1.7 to >77 weeks.	Yes	^412^
Margrolimab, CD47	NCT02953509	I	Combination therapy	DLBCL, FL	≥2	22	ORR 50%, CR 36%	91% of response were ongoing at median follow-up.	No	^407^
Lemzoparlimab, CD47	NCT03934814	I	Combination therapy	R/R NHL	≥2	8	ORR 57%, overall DCR 100%	All responders remained in clinical response at the time of data cutoff.	Yes	^414^
	NCT04202003	I	Monotherapy	R/R AML and MDS	≥2	5	1 patient achieved morphologic leukemia-free state	/	– n–	^415^
Evorpacept, SIRPα/CD47	ASPEN-02	I/II	Combination therapy	R/R MDS	≥1 HMA-based regimen	13	ORR 60% of 5 ND patients, 40% of 5 R/R patients	/	Yes	^417^
TG-1081, CD19/CD47	NCT03804996	I	Comparison of monotherapy and combination therapy	R/R B-cell lymphoma	≥1	14	Monotherapy: ORR 21%; Combination with ublituximab: ORR 44%, CRR 6%	10 patients had durable response at the time of data cutoff.	No	^413^

*FDA* Food and Drug Administration, *R/R* refactory/relapse, *ND* newly diagnosed, *cHL* classical Hodgkin lymphoma, *ASCT* autologous stem cell transplantation, *ORR* overall response rate, *CR* complete response, *PFS* progression-free survival, *OS* overall survival, *DCR* disease control rate, *NHL* non-Hodgkin lymphoma, *PCNSL* primary central nervous system lymphoma, *CNS* central nervous system, *PTL* primary testicular lymphoma, *PMBCL* primary mediastinal large B-cell lymphoma, *DLBCL* diffused large B-cell lymphoma, *CLL* chronic lymphocytic leukemia, *BV* brentuximab vedotin, *RT* Richter transformation, *FL* follicular lymphoma, *MM* multiple myeloma, *AML* acute myelocytic leukemia, *MDS* myelodysplastic syndromes, *HMA* hypomethylating agents, “/”, not available

### ACTs

Our primary focus has been on the large clinical trials of CAR-T cell therapy in various hematologic malignancies. Table 3 gives a summary of representative clinical trials and outcomes of CAR-T cell monotherapy for blood cancers. Tisagenlecleucel, axicabtagene ciloleucel and lisocabtagene maraleuecel are the most representative anti-CD19 CAR-T cell products and they have been studied in a large number of clinical trials. For tisagenlecleucel, ELIANA has indicated its efficacy in pediatric patients with B-ALL.^418^ Other clinical trials with tisagenlecleucel are predominantly focused on B-NHL. Among them, JULIET investigated the CAR-T therapeutic efficacy in R/R DLBCL,^419^ BELINDA raised tisagenlecleucel as second-line treatment,^420^ and ELARA enrolled patients with R/R FL.^421^ The clinical trials for axicabtagene ciloleucel are called ZUMA^422–427^ and cover the treatment of R/R LBCL, B-ALL, and MZL. The most recent one, ZUMA-12, demonstrated the high response rate of axicabtagene ciloleucel as first-line therapy for untreated high-risk LBCL.^427^ Lisocabtagene maraleuecel has fewer trials in comparison with the two products above, but the 2022 TRANSCEND CLL 004 study showed surprising results suggesting an ORR of 82% in patients with R/R CLL and small lymphocytic lymphoma (SLL).^428^ However, the antigen expression of tumor cells still limits the efficacy of CAR-T therapy. Since CD19 may not cover all types of lymphoma subclones,^428–430^ CAR-T cells targeting other highly-specific antigens and dual targets,^302,431–437^ such as CD22, CD19/CD20, and CD30, have been developed as well. The anti-CD30 CAR-T cells have also been developed in HL,^435–437^ and more clinical trials are being conducted to verify their efficacy.

**Table 3:** Representative clinical trials and outcomes of CAR-T monotherapy for treating hematologic malignancies

Type of CAR-T therapy	CAR-T Product & Target	Structure of binding domain & costimulatory domain	Trial	Phase	Type of disease	No. of prior lines of treatment	No. of patients	Response	Survival	FDA approval	Refs.
Monospecific, autologous	Tisagenlecleucel, CD19	FMC63 scFv, 4-1BB	ELIANA	I/II	R/R B-ALL	1–8 (median 3)	75	ORR 81%, CR/CRi 81%	EFS 50%, OS 76% (12months)	Yes	^418^
			JULIET	II	R/R DLBCL	1–6 (median 3)	93	ORR 52%, CRR 40%	RFS 65%, OS 49% (12 months)	Yes	^419^
			BELINDA	III	Aggressive B-NHL	1	322	ORR 46.3%	PFS 25.9% (6 weeks)	Yes	^420^
			ELARA	II	R/R FL	2–13 (median 4)	97	ORR 86.2%, CRR 69.1%	PFS 67% (12 months)	Yes	^421^
	Axicabtagene ciloleucel (KTE- X19), CD19	FMC63 scFv, CD28	ZUMA-1	I/II	R/R LBCL	1–4 (median 3)	101	ORR 82%, CRR 58%	PFS 44% (12months), OS 52% (18 months)	Yes	^422^
	Brexucabtagene, CD19	FMC63 scFv, CD28	ZUMA-2	II	R/R MCL	1–5 (median 3)	71	ORR 93%, CRR 67%	PFS 61%, OS 83% (12months)	Yes	^423^
	Axicabtagene ciloleucel (KTE- X19), CD19	FMC63 scFv, CD28	ZUMA-3	I/II	R/R B-ALL	≥2 (median 2)	55	CR/CRi: 71%	Median RFS 11.6 months, median OS 18.2 months	–	^424^
			ZUMA-5	I/II	R/R FL or MZL	2–3 (median 3)	153	ORR 96%, CRR 77%	PFS 74%, OS 93% (12 months)	–	^425^
			ZUMA-7	III	R/R LBCL	1	179	ORR 83%, CRR 65%	EFS 41% (24 months)	–	^426^
			ZUMA-12	I/II	High-risk LBCL	Untreated	40	ORR 89%, CRR 78%	PFS 75%, OS 91% (12 months)	Yes	^427^
	PD1-19bbz, CD19	Anti-CD19 scFv, 4-1BB	NCT04213469	I	R/R B-NHL	Not treated with CAR-T	8	ORR 100%, CRR 87.5%	PFS 100% (12 months)	No	^465^
	Lisocabtagene maraleuecel, CD19	FMC63 scFv, 4-1BB	TRANSCEND NHL001	I	R/R LBCL	1–4 (median 3)	269	ORR 73%, CRR 53%	Median follow-up for OS 18.8 months	Yes	^267^
			TRANSFORM	III	R/R LBCL	1	92	ORR 79%, CRR 61%	PFS 52.3%, OS 79.1% (12 months)	Yes	^429^
			PILOT	II	R/R LBCL	1 (without HSCT)	61	ORR 49%, CRR 33%	Median PFS 9.03 months	Yes	^430^
			TRANSCEND CLL 004	I	R/R CLL/SLL	≥2 (median 4)	23	ORR 82%, CRR 45%	Median PFS 18 months	Yes	^428^
	CD20 CAR-T	/	ChiCTR2000036350	I	R/R B-NHL	1	15	ORR 100%, CRR 80%	PFS and OS 100% (12.4 months)	No	^431^
	CD22 CAR-T	Anti-CD22 scFv, 4-1BB	NCT02315612	I	R/R B-ALL	≥1 (except 1 untreated)	21	CRR 57%	Among the 12 patients who attained CR, 3 remain in ongoing CR at 21, 9 and 6 months. 8 patients relapsed 1.5–12 months post CAR infusion.	No	^432^
Monospecific, universal (allogenic)	ALLO-501A, CD19	Anti-CD19 scFv, with disrupted TCRα and edited CD52 gene	ALPHA2	I/II	R/R LBCL	≥2	15	ORR and CR 50%	/	No	^456^
	CTX110, CD19	Anti-CD19 scFv, with disrupted TCR and elimination of β_2_-microglobulin gene	CARBON	I	R/R LBCL	≥2	32	ORR 67%, CRR 41%	Nearly 50% patients received CR maintained it out to at least 6 months	No	^459^
	PBCAR0191, CD19	Anti-CD19 scFv, with TCR/CD3 knockout	NCT03666000	I/II	R/R B-ALL	≥2	15	CR/CRi 60%	One patient achieved progression-free more than 250 days. Others had progression or died less than 150 days.	No	^461^
					R/R B-NHL	≥2	13	ORR 77%, CRR 54%	Duration of response assessment is ongoing.	No	^460^
	UCART22, CD22	anti-CD22 scFv,4-1BB	BALLI-01	I	R/R B-ALL	≥1	9	CRi 11%	/	No	^457^
Bispecific, autologous	CD19/22-CAR T	Anti-CD19 FMC63 scFv and anti-CD22 m971 scFv, 4-1BB	NCT03233854	I	R/R B-ALL/ LBCL	≥2	38	B-ALL: ORR 100%, CRR 88%; LBCL: ORR 62%, CRR 29%	B-ALL: median OS 11.8 months, median PFS 5.8 months LBCL: median OS 22.5 months, median PFS 3.2 months	No	^433^
	LV20.19, CD19/20	Anti-CD19 FMC63 scFv and anti-CD20 leu-16 scFv, 4-1BB	NCT03019055	I	R/R B-NHL, CLL	2–12 (median 4)	22	ORR 82%, CRR 64%	Median OS 20.3 months	No	^434^
	TanCAR7 T cells, CD19/CD20	Anti-CD19 FMC63 scFv and anti-CD20 leu-16 scFv, 4-1BB	NCT03097770	I/II	R/R B-NHL	≥1 (most are 3–5)	28	ORR 79%, CRR 71%	PFS 64% (12 months)	No	^302^
Bispecific, universal (allogenic)	CTA101, CD19/CD22	Anti-CD19 FMC63 and anti-CD22 m971 scFv with CRISPR/Cas9-disrupted TCRα region and CD52 gene, 4-1BB	NCT04227015	I	R/R B-ALL	≥1	6	CR/CRi 83.3%	50% patients remained MRD negative at a median follow-up of 4.3 months	No	^301^
Monospecific, autologous	CD30.CAR-Ts, CD30	Anti-CD30 scFv derived from HSR3 antibody, 4-1BB	NCT02690545 NCT02917083	II	R/R HL	2–23 (median 7)	41	ORR 62%, CRR 51%	PFS 36%, OS 94% (1 year)	No	^435^
	CD30.CAR-Ts, CD30		NCT01316146	I	R/R HL/ALCL	≥3	9	ORR 33.3%	HL; 1 patient remained CR for 2.5 years, 1 patient remained CR for 2 years ALCL: 1 patient remained CR for 9 months	No	^436^
	CART-30, CD30		NCT02259556	I	R/R HL	≥10	18	ORR 39%	Median PFS 6 months	No	^437^
Monospecific, autologous	Idecabtagene vicleucel (ide-cel, bb2121), BCMA	Murine anti-BCMA scFv, 4-1BB	NCT02658929	I	R/R MM	3–23 (median 7)	33	ORR 85%, CRR 45%	Median PFS 11.8 months	Yes	^438^
			KarMMa	II	R/R MM	3–16 (median 6)	128	ORR 73%, CRR 33%	Median PFS 8.8 months	Yes	^439^
			KarMMa	III	R/R MM	2–4	254	ORR 71%, CRR 39%	Median PFS 13.3 months	Yes	^440^
	bb21217, BCMA		CRB-402	I	R/R MM	3–17 (median 6)	69	ORR 60%, CRR 28%	Median PFS was not reached	No	^441^
	Ciltacabtagene autoleucel, BCMA	Llama- derived anti-BCMA VHH, 4-1BB	CARTITUDE-1	I/II	R/R MM	4–8 (median 6)	97	ORR 97%, sCR 67%	PFS 77%, OS 89% (12 months)	Yes	^442^
	CT103A, BCMA	Fully human anti-BCMA scFv, 4-1BB	ChiCTR1800018137	I	R/R MM	3–6 (median 4)	18	ORR 100%, sCR/CR 72.2%	PFS 58.3% (12 months)	No	^278^
	P-BCMA-101, BCMA	Murine anti-BCMA scFv, 4-1BB	PRIME	I/II	R/R MM	2–18 (median 8)	53	ORR 57%	/	No	^443^
	Orva-cel, BCMA	Humanized anti-BCMA scFv, 4-1BB	EVOLVE	I/II	R/R MM	3–18 (median 6)	62	ORR 92%, CRR 36%	/	No	^444^
	CT053, BCMA	Humanized anti-BCMA scFv, 4-1BB	Lummicar-2	I/II	R/R MM	2–11 (median 4)	14	ORR 100%, sCR/CR 40%	/	No	^445^
Monospecific, universal (allogenic)	ALLO-715, BCMA	Humanized anti-BCMA scFv with CD52 & TCR knockout, 4-1BB	UNIVERSAL	I	R/R MM	3–11 (median 5)	43	ORR 55.8%	Median DOR 8.3 months	No	^446^
Monospecific, autologous	MCARH109, GPRC5D	Humanized anti-GPRC5D scFv, 4-1BB	NCT04555551	I	R/R MM	4–14 (median 6)	17	ORR 71%, CRR 35%	PFS 50% (10.1 months)	No	^299^
	CC-95266, GPRC5D	Anti-GPRC5D scFv, 4-1BB	NCT04674813	I	R/R MM	≥3	17	ORR 89%, CR 47%	PFS 88% (at the time of analysis)	No	^448^
	OriCAR-017, GPRC5D	Anti-GPRC5D scFv with Ori, 4-1BB	POLARIS	I	R/R MM	≥3	10	ORR 100%, sCR 60%	PFS 80% (12months)	No	^290^
Combination of two monospecific CAR-T	CD19 and BCMA CAR-T	Humanized anti-CD19 scFv, murine anti-BCMA scFv, 4-1BB	ChiCTR-OIC-17011272	II	R/R MM	5–8 (median 6)	21	ORR 95%, sCR 43%, CR 14%	PFS 85% (602 days)	No	^450^
Bispecific, autologous	BM38, BCMA/CD38	Humanized anti-BCMA/CD38 scFv, 4-1BB	ChiCTR1800018143	I	R/R MM	2–9 (median 4)	23	ORR 87%, sCR/CR 54.5%	Median PFS 17.2 months	No	^291^
	BCMA/CS1	Murine anti-BCMA/CS1 scFv, 4-1BB	NCT04662099	I	R/R MM	≥2	16	ORR 100%, sCR 31%	OS 83.9%, PFS 55.2% (12 months)	No	^449^
Monospecific, HSCT donor-derived (allogenic)	CD7 CAR-T	Anti-CD7 scFv, 4-1BB	ChiCTR2000034762	I	R/R T-ALL	2–4 (median 3)	20	ORR 95%, CRR 90%	/	No	^284^
Monospecific, autologous	nanobody-derived fratricide-resistant CD7-CAR T	Humanized anti-CD7 nano- body-derived CAR, ICOS and 4-1BB	NCT04004637	I	R/R T-ALL/LBL	≥4	8	CRR 87.5%	/	No	^285^
Monospecific, autologous/donor-derived	NS7CAR, CD7	/	NCT04572308	I	R/R T-ALL/LBL	≥1	20	CRR 95%	/	No	^454^
Monospecific, universal (allogenic)	RD13-01, CD7	Anti-CD7 scFv with TCR/CD3 knockout and NK cell inhibitor, 4-1BB	NCT04538599	I	R/R T-ALL/LBL	2–7 (median 4)	12	ORR 81.8%, CRR 63.6%	/	No	^281^
Monospecific, autologous	CAR-T-38, CD38	Anti-CD38 scFv same as daratumumab, CD28 and 4-1BB	NCT04351022	I	R/R AML	>1 (received HSCT)	6	CR/CRi 66.7%	Median OS 7.9 months, LFS 6.4 months	No	^289^
	NKG2D CAR-T	Humanized NKG2D gene, without co-stimulation domain	NCT02203825	I	R/R AML/MDS/MM	0–4	12	ORR not seen	Median OS 4.7 months	No	^451^
	CLL-1 CAR-T	Anti-CLL-1 scFv, 4-1BB	ChiCTR2000041054	I	R/R AML	2–10 (median 5)	10	CR/CRi 70%	/	No	^452^
Monospecific, universal (allogenic)	UCART123v1.2, CD123	Anti-CD123 scFv, with disrupted TRAC and CD52 genes	Ameli-01	I	R/R AML	≥2 or received prior allogenic HSCT	8	ORR 25%	/	No	^453^

*CAR-T* chimeric antigen receptor T cell, *FDA* Food and Drug Administration, *scFv* single chain variable fragment, *R/R* relapsed or refractory, *B-ALL* B-cell acute lymphoblastic leukemia, *ORR* overall response rate, *CR* complete response, *CRi* complete remission with incomplete hematological recovery, *EFS* event-free survival, *OS* overall survival, *DLBCL* diffused large B-cell lymphoma, *RFS* relapse-free survival, *NHL* non-Hodgkin lymphoma, *PFS* progression-free survival, *FL* follicular lymphoma, *CRR* complete response rate, *LBCL* large B-cell lymphoma, *MCL* mantle cell lymphoma, *MRFS* median relapse-free survival, *MZL* marginal zone lymphoma, *HSCT* hematopoietic stem cell transplantation, *CLL* chronic lymphocytic leukemia, *SLL* small lymphocytic lymphoma, *HL* Hodgkin lymphoma, *ALCL* anaplastic large cell lymphoma, *MM* multiple myeloma, *sCR* stringent complete response, *DOR* duration of response, *T-ALL* T-cell acute lymphoblastic leukemia, *T-ALL/LBL* T-cell acute lymphoblastic leukemia/lymphoma, *AML* acute myelocytic leukemia, *LFS* leukemia-free survival, *MDS* myelodysplastic syndromes, *TRAC* T-cell receptor alpha constant, “/”, not available

In addition to lymphoma and leukemia, CAR-T cells have also made great progress in treatment of MM.^278,438–446^ Idecabtagene vicleucel (ide-cel) and ciltacabtagene autoleucel (cilta-cel) have already been approved by the FDA based on responses and safety demonstrated in the KarMMa and CARTITUDE-1 trials.^439,440,442^ Meanwhile, more companies have launched CAR-T cell products for MM, such as orva-cel, P-BCMA-101.^443,444,446–449^ CAR-T cell products against the new target, GPRC5D, have also been developed without delay. Usually, patients enrolled in CAR-T clinical trials have a good baseline condition. This is to prevent them from not being able to tolerate side effects such as CRS. However, the first clinical trial of the GPRC5D target was conducted in patients with poor baseline conditions who had received multiple lines of therapy.^299^ The clinical results also showed a high level of safety and efficacy, taking the development of CAR-T to a new level. In other types of hematological malignancies, CAR-T therapy is still in the exploratory phase of development. A single-center, single-arm, phase 2 trial assessed the activity and safety of a combination of humanized anti-CD19 and anti-BCMA CAR T cells in patients with R/R MM and confirmed that this combined infusion is feasible with ORR of 95% and CRR of 43%.^450^ As for AML, CAR-T therapy seems to be less effective due to the lack of appropriate tumor targets and is still being explored in preclinical and clinical studies.^289,451–453^ The difficulty of manufacturing cell products using autologous T cells is the major problem facing CAR-T therapy in T-ALL. As a result, several institutions have developed donor-derived CAR-T cells and have conducted clinical trials to confirm the efficacy and safety of these CAR-T cells.^281,284,454^ The donor-derived CAR-T cells suggested encouraging effects, especially in those patients who received allo-HSCT.^455^

The universal CAR-T cells, also known as “off-the-shelf”, can overcome the problem of long period of manufacturing and enable those patients whose T cells are under poor condition to receive CAR-T therapy. Due the heterologous nature of allogeneic CAR-T cells, many products are designed to knock out of the TCR or edit the CD52 gene to overcome GVHD and HVGD (host versus graft disease). It is slao essential to examine the safety and in vivo persistence of universal CAR-T cells through clinical trials.^446,456–460^ Anti-CD19 universal CAR-T cells, like PBCAR0191 and bispecific universal CAR-T CTA101, also showed high rates of CR (60% and 83.3%).^301,461^ 81.8% of patients showed OR after RD13-01 infusion (CRR 63.6%) without GVHD and severe CRS.^458^ A phase 1 UNIVERSAL trial reported a first-in-class, allogeneic, anti-BCMA CAR-T cell therapy (ALLO-715) engineered to abrogate GVHD and minimize CAR-T rejection. ALLO-647 (anti-CD52 antibody) was used for lymphodepletion with fludarabine and/or cyclophosphamide before ALLO-715 infusion. There was obvious expansion in 83.3% of patients yet 63.3% of patients showed undetectable levels of CAR-T cells by the day 28.^446^ Overall, universal CAR-T cells have made some progress, but the clinical safety, efficacy and the duration of response of these products still requires further observation.

Although CAR-T cell therapy has achieved outstanding results when used as a monotherapy, there are still certain patients who do not benefit from it and further research is urgently needed to improve and prolong the efficacy of CAR-T therapy. Therefore, researchers are focusing on the combination of other immunotherapies with CAR-T cell therapy. The immune checkpoint molecule PD-1 on the surface of CAR-T cells has been reported to be overexpressed due to T-cell overactivation and thus blocking the PD-1/PD-L1 pathway might effectively restore the function of CAR-T cells.^462^ Clinical trials have been performed with the combination of PD-1 blockers and CAR-T therapy and the results have been encouraging. A phase II clinical trial of anti-CD30 CAR-T treatment in combination with PD-1 inhibitor in R/R CD30-positive lymphoma has been conducted. Among the 12 patients who were evaluated for response, the ORR was 91.7% and the CRR was 50%. And 7 patients maintained their response until the end of the follow-up.^463^ Additionally, the combination of CD19 CAR-T cells and PD-1 blockade was proven to reduce intracranial tumor burden in a patient with centrally-invasive lymphoma.^464^ However, some researchers have chosen to construct endogenous PD-1 dominant-negative receptors (DNRs) within CAR-T cells to allow them to bind both TAA and PD-1 on tumor cells, ensuring that CAR-T function is not inhibited.^465,466^ In combination with CAR-T cell therapy, HSCT is also a popular alternative. Bridging therapy with donor CAR-T cells after allogeneic transplantation can have shown a prolonged effect on the efficacy of the transplant.^467,468^ According to the results from a retrospective study, haplo-HSCT with pre-transplant negative MRD after CAR-T cell therapy can significantly improve LFS (leukemia-free survival) and OS in patients with R/R B-ALL.^469^ This finding was confirmed in subsequent clinical trials. In the subgroups of patients who achieved MRD-negative CR after CAR-T cell therapy, event-free survival (EFS), and RFS were significantly prolonged by allo-HSCT.^470^ As a result, CAR-T therapy followed by transplantation can improve survival in a similar manner and is a viable option for achieving a durable remission of the disease.

Currently, CAR-NK is also a hot topic of research, with the major advantage that NK cells are able to be produced from healthy donor-derived PBMC, core blood, or iPSCs (induced pluripotent stem cells) without any appreciable toxicity. 11 patients were treated in a phase 1/2 study with anti-CD19 CAR-NK derived from core blood. Among them, 8 patients experienced a response and 7 of them experienced a CR. The infused CAR-NK cells proliferated and persisted in vivo at low levels for at least 12 months.^471^ Although it was not effective in B-ALL patients unfortunately, NKX019 showed a favorable efficacy in R/R B-NHL patients and the ORR was 83% and CRR was 50% in the higher-dose group.^472^ Besides, NKX101 targeted NKG2D (natural killer cell group 2 member D) and achieved an ORR of 47% in all R/R AML patients enrolled.^473^ For R/R MM, FT576 was proved to be safe and tolerates without CRS, GVHD, or neurotoxicity and was determined a recommended dose in a phase 1 trial.^474^ In addition, more researches on CAR-NK cells are still in the pre-clinical stage or early clinical trials.^308,475^ Further research is also needed to perfect the design and manufacturing to improve the efficacy and durability of CAR-NK cells.^476,477^

## AEs and toxicity management

The era of immunotherapy has brought revolutionary breakthroughs for hematologic malignancies. These therapies are designed to stimulate the immune system to recognize and attack cancer cells, thereby extending survival and improving outcomes. However, immunotherapy poses new clinical problems and challenges for hematologists due to its toxicity, which is different from traditional chemotherapy, depending on the specific mechanism of action.^478^ The occurrence of AEs cannot be ignored and can affect almost all organs and systems. These AEs further impede the clinical application of immunotherapy and, in severe cases, even threaten the patient’s life.^478^ Therefore, the need for and importance of toxicity management has become increasingly apparent. Treatment of AEs usually depends on the organ involved and the severity of symptoms. These toxicities often require specific management, including steroids and immunomodulatory therapy, for which consensus guidelines have been proposed and published. Here, we summarize the typical AEs associated with various immunotherapies, including HSCT, antibody-based therapies, ICIs, and CAR-T cell therapies, and then discuss their clinical management.

### AEs of HSCT

Although HSCT can give some patients a chance at a cure, it is not an easy decision to be made. Transplantation has been a cure for thousands of patients with lethal forms of cancer. However, there can still be life-threatening risks and complications. Most of the side effects that can occur shortly after the transplant are the result of the bone marrow being destroyed by drugs or radiation just before the transplant. Others may be due to side effects of the conditioning treatments themselves. A short-term side effect that can occur with chemotherapy and radiation is mucositis. To prevent this, doctors often give anti-nausea medication at the same time as chemotherapy. Patients can easily get serious infections for at least the first six weeks after the transplant until the new stem cells start to produce white blood cells.^479–481^ To prevent possible infections, antibiotics are used until the blood counts reach a certain level. It can take about 6 months to 1 year after the transplant for the immune system to take effect. Injuries and bleeding are other potential risk because the conditioning regimen can damage the body’s ability to generate platelets. Pneumonitis is a type of inflammation of the lung tissue that’s most commonly seen in the first 100 days after the transplant. However, some kinds of lung problems can occur much later after a transplant. Pneumonia caused by an infection is more common, but pneumonitis can also be caused by radiation, GVHD, or chemotherapy, rather than by the infection itself. Pneumonitis can be particularly severe if the patient has received total body irradiation with chemotherapy as part of the pre-transplant regimen. Acute kidney injury (AKI) directly related to stem cell transplant encompasses a wide range of both structural and functional disorders, which may be of the vascular (hypertension, thrombotic microangiopathy), glomerular (albuminuria, nephrotic glomerulopathies), and/or tubulointerstitial type.^482–484^ AKI is a common complication following stem cell transplantation, affecting ~10–73% of patients.^482^ A serious side effect in which tiny veins and other blood vessels in the liver become blocked is a hepatic veno-occlusive disease (VOD).^485^ It is very rare and is only seen in people who have had an allogeneic transplant.^485,486^ The onset of VOD is usually about 3 weeks after transplantation. It is more common in older patients who have had liver disease before the transplant and in patients who have acute GVHD. The symptoms are yellow skin and eyes, dark urine, tenderness under the rib cage and a rapid increase in body mass.^485^ It is life-threatening, so it is very important to recognize and diagnose VOD at an early stage.^487,488^

#### GVHD

GVHD is a leading contributor to mortality and morbidity after allo-HSCT.^489,490^ The donated immune cells may also attack some of the organs, most typically the skin, the gastrointestinal tract and the liver. As a result, there may be some changes in the functioning of the body’s organs and an elevated risk of infections.^491^ GVHD reactions are very common and can range in severity from barely noticeable to life-threatening.^39,492,493^ Acute GVHD can occur between 10 and 90 days after the transplant and lasts for a short period of time. Chronic GVHD has a later onset and longer duration. The patient may experience one or both types of GVHD, or neither type of GVHD. Acute GVHD develops in approximately one-third to one-half of allogeneic transplant recipients. It is less frequent in the younger patients and the ones with a more closely matched HLA. A rash, burning and redness of the skin on the palms and the soles of the feet are usually the first symptoms. The rash may spread to the rest of the body. Other symptoms may include nausea, vomiting, stomach cramps, decreased appetite, jaundice, abdominal pain, and weight loss. Medications that can suppress the immune system may be given to prevent acute GVHD, such as steroids (glucocorticoids), methotrexate, cyclosporine, tacrolimus, or some types of mAbs.^492,494^ These are administered before acute GVHD begins to occur. The risk of acute GVHD can also be reduced by the removal of immune cells from the donor stem cells prior to transplantation. However, this also increases the risk of viral infection, leukemic recurrence and graft failure. Researchers are exploring new ways to remove allo-activated T cells from donor transplants, which would reduce the severity of GVHD while still allowing donor T cells to destroy any remaining cancer cells. Mild cases of GVHD can usually be treated with topical steroid medications. More severe cases of GVHD may need to be treated with oral steroid medications or intravenous steroid medications. Chronic GVHD, which can lead to significant morbidity and mortality, usually occurs within one year of allo-HSCT.^495^ When engrafted immune cells attack host cells, it causes inflammation and fibrosis in various types of tissues and multiple organ systems, such as the esophagus, gastrointestinal tract, neuromuscular system, genitourinary tract, liver, lungs, mouth, eyes, muscles, and joints.^495,496^ Symptoms of chronic GVHD may include dry eyes, raised or discolored rash, thickened skin, swollen abdomen, yellowing of the skin and eyes, dry mouth, breathlessness, difficulty swallowing, fatigue, muscle weakness, and joint stiffness. Chronic GVHD can also be treated with immunosuppressive drugs, but these drugs increase the risk of infection. Most patients who have chronic GVHD will be able to stop taking the immunosuppressive medication if their symptoms are getting better.

#### Secondary cancers

It is possible for the original type of cancer to come back and for a second type of cancer to develop after the transplant.^497–499^ The cancers that can develop are solid tumors in various organs, leukemia and MDS. They tend to occur a few years or even longer after engraftment.^500,501^ Post-transplant lymphoproliferative disorder (PTLD) is an out-of-control growth of lymphocytes that can occur following alloHSCT.^502,503^ Normally, T cells assist the body in getting rid of virally infected cells. The pretransplant treatment compromises the immune system, enabling EBV infections to get out of control. PTLD after allo-HSCT is relatively rare and generally occurs within one to six months. The symptoms of PTLD consist of swollen lymph nodes, fever and chills.^503^ Although there is no standard treatment, the usual management is to reduce the use of immunosuppressive drugs and encourage the patient’s immune system to fight back. Other options involve infusing lymphocytes to boost the immune response and the administration of antiviral drugs.^503–506^

### AEs of antibody-based therapies

Antibody-based drugs have been generally considered to be less toxic than cytotoxic chemotherapeutics used for cancer therapy, while some of these elements may be recognized as foreign substances and thereby cause hyperactivation of immune and innate reactions. A wide spectrum of AEs to antibodies is observed, necessitating efforts to identify, manage and minimize side effects. Some toxicities result from the binding of a therapeutic antibody to its target antigen on normal cells, which refers to the “on-target, off-tumor” toxicity. Therefore, the manifestations of such toxicities are dependent on the target of antibody drugs. For example, rituximab can cause profound first-dose toxicity related to the rapid lysis of normal and malignant B cells that bear the target antigen, CD20.^507^ Acute reactions can be caused by a variety of mechanisms, including acute IgE-mediated hypersensitivity and anaphylactoid reactions against the antibodies, serum sickness, tumor lysis syndrome (TIS) and CRS.^508,509^ Clinical manifestations include local skin reactions at the injection site, fever and influenza-like syndrome and potentially fatal acute anaphylaxis and systemic inflammatory response syndrome (SIRS). Hypersensitivity reactions may be severe enough to require aggressive management and discontinuation of therapy. Meanwhile, these antibodies have immunomodulatory effects thus they can also induce various autoimmune diseases. AEs are also common in patients receiving bsAbs, with the majority of them being grade 3 or higher-grade AEs. A phase II study which included R/R B-ALL patients revealed that the common AEs during blinatumomab therapy included pyrexia (81%), fatigue (50%), headache (47%), tremor (36%), and leukopenia (19%), and most of the AEs occurred during the first cycle of administration.^357^ In another trial, patients in the blinatumomab group suffered more AEs but the rate of serious AEs in the blinatumomab group was lower than that in the chemotherapy group.^510^ The T-cell activation induced by BiTE poses the risk of unique complications such as CRS, neurotoxicity and TIS.^511^ Moreover, severe CRS and neurological toxicity are the main reasons for the interruption of BiTE therapy, which can be controlled by close clinical monitoring and timely preventive or therapeutic intervention.

More importantly, the immunogenicity of antibodies is not only related to the percentage of homology, as specific amino acid changes at some positions can also affect immunogenicity. Drug-induced immunogenicity has been recognized as a major challenge in the development of antibodies, resulting in adverse effects and loss of efficacy. Drug administration to patients may induce humoral immune responses, causing the formation of anti-drug antibodies (ADAs). ADAs can complex with circulating therapeutic antibodies, making it difficult to achieve efficacious levels of circulating therapeutic antibodies. ADAs may not only inactivate the drug and cause a loss of targeting and/or increased clearance of ADA-drug complexes but also induce increased toxicity caused by the immune response that accompanies ADA formation, loss of drug targeting, or formation of highly immunogenic complexes.^508,512^ Therefore, ADA assays should be rationally designed to allow an understanding of the characteristics and consequences of the detected ADAs.

Both the cytotoxic molecules and the antibody portion of ADCs can affect normal cells, resulting in “off-tumor” toxicities.^513^ These “off-tumor” toxicities can be divided into “on-target” and “off-target” toxicities. The “on-target” toxicity is caused by ADCs killing normal tissues that express the target antigen, while “off-target” toxicity refers to the killing of ADCs in tissues that do not express the target antigen. Based on clinical observations, “on-target toxicity” caused by small molecule toxins is the major source of adverse effects of ADCs. Both antibody-mediated ADCC and CDC effects can occur in normal cells expressing the target antigen and lead to adverse reactions such as secondary kidney injury. In addition, like mAbs and bsAbs, ADCs can block the signaling of target antigens in normal cells, resulting in adverse reactions such as lung injury and liver toxicity. The “off-target toxicity” can be caused by the shedding of cytotoxic molecules into the circulation, bystander effect on normal cells and endocytosis and uptake of ADC by normal cells, causing normal cells to suffer damage from cytotoxic molecules.^514^ The main victims are lymphocytes, granulocytes, and platelets in the bloodstream, followed by kidneys, lungs, nerves, skin and other tissues, causing clinically observed side effects similar to those of chemotherapeutic drugs.^513^ Common AEs include fever, nausea, infection, vomiting, and stomatitis. Severe side effects were low blood counts, liver damage including hepatic VOD, infusion-related reactions and hemorrhage. Treatment discontinuation should be considered for patients who develop obvious signs or symptoms of anaphylaxis, including severe respiratory symptoms or clinically significant hypotension. Premedication with a corticosteroid, antihistamine and acetaminophen is recommended about one hour prior to the administration of ADC agent.^515^

### Immune-related adverse effects during ICI therapy

AEs linked to the use of ICIs are referred to as immune-related AEs (irAEs). These primarily include immune-related skin toxicity, endocrinopathies, hepatotoxicity, gastrointestinal toxicity, pulmonary toxicity, hematologic toxicity, central nervous system toxicity, cardiovascular toxicity, rheumatologic toxicity, immunotoxicity, renal toxicity, ocular toxicity, etc.). The incidence of irAEs with single-agent ICIs varies depending on the single agent, the tumor type and the disease setting.^478^ Grading of irAEs is in accordance with the Common Terminology Criteria for AEs (CTCAE). Recommendations for the monitoring, diagnosis and treatment of irAEs are available in consensus guidelines from the American Society of Clinical Oncology (ASCO), the European Society of Medical Oncology (ESMO), the NCCN and the Society for Immunotherapy of Cancer (SITC).^516–520^ In principle, there are four sequential steps in the management of irAE: (i) diagnosing and grading irAEs, (ii) ruling out differential diagnoses and workup before immunosuppression, (iii) selecting the appropriate immunosuppression strategy for grade ≥2 cases, and (iv) actively evaluating at 72 h to make treatment adjustments.^521^ While the management depends on the affected organ system, in general, ICI therapy should be followed with close monitoring for grade 1 toxicities, except for some neurologic, hematologic and cardiovascular toxicities.^521^ ICI therapy may be discontinued for the majority of grade 2 toxicities. Consideration should be given to resuming ICI therapy if symptoms revert ≤grade 1. Suspension of ICIs and initiation of high-dose corticosteroids is generally warranted for grade 3 toxicities. Corticosteroids should be tapered over the course of a minimum of 4 to 6 weeks. For grade 4 toxicities, permanent discontinuation of ICIs is generally recommended. This does not apply to endocrinopathies that have been controlled with hormone replacement therapy.^521,522^

#### Immune-related skin toxicity

Dermatologic toxicity seems to be one of the most commonly occurring AEs during treatment with ICIs.^523–527^ Maculopapular eruption and pruritus are the most common symptoms. Serious dermatologic toxicities, such as Stevens-Johnson syndrome (SJS), toxic epidermal necrolysis (TEN), drug rash with eosinophilia and systemic symptoms (DRESS) and acute febrile neutrophilic dermatosis (Sweet syndrome) and systemic symptoms, are rare.^524^ Although serious cutaneous AEs are rare, cutaneous side events can have a significant impact on the quality of life, reduce patient compliance and lead to dose adjustments or even discontinuation of treatment.^525,528^ The question of whether ICIs can be resumed after grade 3 skin toxicity has been reduced to grade 1 or less with hormonal therapy should be discussed with the dermatologist. ICIs should be discontinued permanently and patients referred to a dermatologist if severe (grade 4) herpetic dermatoses occur.^516–520^

#### Immune-related endocrinopathies

Immune-related endocrinopathies involving the thyroid gland (hypothyroidism or thyrotoxicosis), pituitary hypophysitis, adrenal glands (adrenal insufficiency), and pancreas (diabetes mellitus) are a frequent cause of acute and prolonged morbidity and may even be fatal.^529–533^ Mild symptoms can be managed with continuation of ICI therapy with appropriate hormone replacement therapy; moderate symptoms require immediate discontinuation of ICI therapy and moderate symptoms require oral prednisolone 0.5–1 mg/kg; severe symptoms require intravenous prednisolone 1 mg/kg (methyl) tapered to 5 mg depending on symptom control, but hormone therapy cannot be discontinued. Routine monitoring of blood glucose levels is recommended in patients treated with ICIs and caution is required for the development of life-threatening ketoacidosis.^533^ Unlike other irAEs, endocrinopathies are almost always permanent and require lifelong hormone replacement.^520^ Due to the relatively vague nature of the symptoms associated with these endocrinopathies, prompt recognition and initiation of treatment can have a dramatic impact on a patient’s health and quality of life.

#### Immune-related hepatotoxicity

ICI-associated hepatitis is mainly characterized by elevated levels of transaminases with mildly elevated levels of bilirubin.^534–536^ The diagnosis of immune-related hepatitis may be aided by laboratory tests, which include viral serologies, liver ultrasound, cross-sectional imaging, and liver biopsy.^534^ Serum transaminase and bilirubin levels are recommended for all patients receiving ICI therapy before each treatment cycle to assess liver function. Hepatitis is usually asymptomatic, with some patients presenting with low-grade fever and malaise, which may be associated with transaminase levels.^535^ Most patients with immune-related hepatitis respond to corticosteroids, but a substantial fraction require treatment with a secondary immunosuppressive agent.^534^ It is also important to be alert to cases in which rebound transaminase levels or even fulminant hepatitis have been observed clinically, even after transaminase levels have been reduced to normal. The patient’s clinical presentation and serologic test results must continue to be monitored after recovery of liver function.

#### Immune-related gastrointestinal toxicity

Immune-related gastrointestinal toxicity is also a common adverse effect of ICI therapy, mainly manifested as diarrhea, colitis and small bowel inflammation.^537–540^ The risk of gastrointestinal side effects is much higher with anti-CTLA-4 mAbs than with anti-PD-1/PD-L1 mAbs and can occur at any time during treatment, even months after treatment has ended. The median time for gastrointestinal side effects was 3 months. Following the diagnosis of immune-related gastrointestinal adverse events, the clinical selection of treatment options was based on the severity and duration of diarrhea. In addition to discontinuation of ICI, patients with grade 1 diarrhea may be treated with antidiarrheal drugs alone (loperamide, etc.) based on active rehydration and correction of water-electrolyte imbalance; for grade 2 diarrhea and above, glucocorticoids are the first recommended treatment; for grade 3–4 diarrhea or if glucocorticoid therapy is ineffective, immunosuppressive agents (e.g, infliximab, vedolizumab) are also an option.

#### Immune-related pulmonary toxicity

Immune-related pulmonary toxicity is a heterogeneous group of disorders that includes various clinical manifestations such as interstitial lung disease (ILD) or pneumonitis and rarer presentations such as bronchiolitis or pulmonary sarcoidosis.^519,541–543^ Immune-related pulmonary toxicity usually appears in the first few months and is accompanied by non-specific clinical manifestations but with suggestive radiologic signs.^544^ Exploratory endoscopy, including bronchoalveolar lavage and transbronchial lung biopsies, can further refine the diagnosis by ruling out a lung infection and demonstrating lymphocytic alveolitis. Any new respiratory symptoms, such as upper respiratory tract infection, cough, wheezing and dyspnea, should prompt a chest CT (computerized tomography) scan. Follow-up and monitoring are recommended for those who have imaging changes only and no clinical symptoms (grade 1); prednisolone therapy is suggested for those with mild to moderate symptoms (grade 2) and those with severe or life-threatening symptoms. For grade 2 pneumonia, clinical symptoms should be evaluated every 2–3 days; for grade 3–4 pneumonia, clinical symptoms and imaging should be evaluated after 2 days of treatment and if there is no evidence of improvement, immunosuppressive agents such as infliximab, cyclophosphamide, or mycophenolate mofetil can be considered.^517–519^

#### Other rare immune-related toxicities

Rare immune-related toxicities during ICI treatment mainly include neurotoxicity, cardiotoxicity, rheumatologic immunotoxicity, hematologic toxicity, neuromuscular toxicity, and nephrotoxicity.^545–553^ However, they are still reported in 1–12% of cases and are more common in patients receiving combination therapy. As an increasing number of patients with cancer are being treated with checkpoint inhibitors, the balance between clinical benefits and treatment-related toxicities for each patient is becoming more challenging.^554^ Rarity is not the same as insignificance and the extent of damage to patients after its occurrence can even lead to death in a short period of time. In general, patients who experience a severe grade 3 or 4 irAE during ICI therapy are at risk of experiencing serious toxicities when rechallenged with checkpoint inhibitors.^555^

### CAR-T therapy-related toxicities

Cytokine Release Syndrome (CRS) and neurotoxicity are the most common and unique toxicities associated with CAR T-cell therapies,^556–567^ and they are completely different from the irAEs that are associated with the treatment of ICIs. CAR-based therapies have the advantage of higher targeting specificity over conventional chemotherapy and radiotherapy. However, like antibody-based therapies, targeted antigens od CAR-T cells are also expressed in normal cells, such as CD19 in the normal B-cell lineage. The “on-target, off-tumor” toxicity is widespread, although a large part of others has not been identified or overlapped with other symptoms. Some toxicities, such as hypogammaglobulinemia, are a direct consequence of the “on-target, off-tumor” effects of the CAR-T cells and others may be an indirect result of the immunosuppressed state of the host.^565^ For early recognition of potential toxicities and timely intervention, clinical monitoring before, during and after CAR-T cell therapy is critically required. Perhaps more importantly, with the appropriate management strategies, some of these toxicities associated with CAR-T therapies can be reversed with appropriate monitoring and management (Table 4).^559,568–570^

**Table 4:** Monitoring and management of toxicities associated with CAR-T therapy

CAR-T therapy-related AEs	Biomarkers to monitor	Toxicity management
CRS	CRP, IFN-γ, IL-1, IL-2, IL2Rα, IL-4, IL-6, IL-8, IL-10, TNF-α, granzyme B, MIP-1α, MCP-1, and GM-CSF in PB	Grade 1: broad-spectrum antibiotics along with supportive care; Grade ≥2: Intravenous tocilizumab ≤4 doses; Grade ≥3 and in cases of grade 2 toxicity with sustained hypotension after anti-IL-6 therapy: add corticosteroids; Refractory to both tocilizumab and corticosteroids: use other agents include the Janus-associated kinase inhibitor, cyclophosphamide, extracorporeal cytokine adsorption with continuous renal replacement therapy, IVIG and anti-thymocyte globulin.
Neurotoxicity	IL-1, IL-6, IFN-γ, TNF-α/β, CRP, coagulation markers, ferritin in PB; MCP1, IL-6, IL-8 in CSF; ICE score	Grade ≥1 ICANS: monitoring, supportive care and corticosteroids alone; Tocilizumab was not recommended unless patients have concurrent CRS.
HLH/MAS	Blood routine test; IFN-γ, IL-6, GM-CSF, CRP, ferritin in PB	Suppress the overactive immune cells; Corticosteroids, anakinra or intrathecal cytarabine can be considered in cases when the HLH/MAS is caused by resistance to tocilizumab.
CARAC	Primary coagulation markers including platelet count in PB, APTT, PT, FIB, FDP, and D-dimer; test for CRS	Management of CRS; replacement therapy to decrease the risk of bleeding and control active bleeding, including the transfusion of platelet, fresh frozen plasma and prothrombin complex concentrates and fibrinogen and cryoprecipitate; anticoagulant therapy and/or antifibrinolytic therapy should be used as appropriate for patients with high-grade CRS.
Cytopenia	Blood routine test, CRP, ferritin in PB; cytology of blood marrow	Growth factors, thrombopoietin receptor agonists, stem cell enhancement, transfusion support; Elimination of infectious risk
Hypogammaglobulinemia	Gammaglobulinemia in PB	Intravenous or subcutaneous immunoglobulin G
Infection	IL-6, CRP in PB; lymphocyte count; CT of lungs; viral and bacterial etiologic test	Provide antibacterial or antifungal prophylaxis; For certain patients with concurrent severe or recurrent infections and hypogammaglobulinemia: IVIG is recommended as replacement treatment.
ADAs	Detection of ADAs in serum (HAMA is the ADA occurred in CAR-T therapy with murine-derived scFv)	Secondary reinfusion by altering the target and strengthening lymphodepletion.

*CAR-T* chimeric antigen receptor T cell, *AEs* adverse effects, *CRS* cytokine release syndrome, *CRP* C-reactive protein, *IFN* interferon, *IL* interleukin, *TNF* tumor necrosis factor, *MIP* macrophage inflammatory protein, *MCP* monocyte chemoattractant protein, *GM-CSF* granulocyte/macrophage colony-stimulating factor, *PB* peripheral blood, *IVIG* intravenous immunoglobulin G, *CSF* cerebrospinal fluid, *ICE* immune effector cell associated encephalopathy, *ICANS* immune effector cell-associated neurotoxicity syndrome, *HLH/MAS* Hemophagocytic lymphohistiocytosis/macrophage activation syndrome, *CARAC* CAR-T therapy-associated coagulopathy, *APTT* activated partial thromboplastin time, *PT* prothrombin time, *FIB* fibrinogen, *FDP* fibrin degradation products, *CT* Computed Tomography, *ADA* anti-drug antibody, *HAMA* human-anti-mouse antibody, *scFv* single chain variable fragment

#### CRS

CRS is the most common life-threatening adverse event associated with CAR T-cell therapy. Variable incidence of CRS has been reported with different CAR T-cell therapies due to differences in grading scales used to assess CRS severity, CAR T-cell design and generation and clinical trial design.^565,571^ The typical time to the onset of CRS ranges from 2 to 3 days, with a persistent duration of 7 to 8 days, although CRS can occur within a few hours or as late as 10 to 15 days after CAR-T cell infusion. The onset of CRS is usually characterized by fever and constitutional symptoms such as malaise and anorexia. In severe cases, CRS also manifests with features of a systemic inflammatory response. These include hypotension, hypoxia, cytopenia, coagulopathy and even organ dysfunction. The organ dysfunction may be the secondary effect of hypotension or hypoxia, but it may also be a direct result of the release of cytokines. Organ dysfunction can be prevented or even reversed in the majority of patients if the symptoms and signs of CRS are recognized and addressed in a prompt and timely manner.^564^ CAR-T cell-mediated cancer elimination was also the trigger for the systemic inflammatory response, which is the hallmark of CRS.^556,558,572^ Thus, from a clinical standpoint, the most important management to overcome CRS is to block the feedback loop of cytokines.^573^ Cytokines and markers of inflammation that have been implicated in more severe CRS are C-reactive protein (CRP), ferritin, interferon (IFN)-γ, IL-1, IL-2, soluble IL2-Rα, IL-4, IL-6, IL-8, IL-10, tumor necrosis factor (TNF)-α, granzyme B, granulocyte/macrophage colony-stimulating factor (GM-CSF), macrophage inflammatory protein-1α (MIP-1α), and monocyte chemoattractant protein-1 (MCP-1).^564,574–577^ A number of risk factors for severe CRS have been implicated, although these vary between different studies and likely between different indications. In general, these include an increased CAR-T cell expansion and a higher tumor burden.^578,579^ Moreover, bone marrow (BM) suppression is also considered a determinant of the occurrence and evolution of CRS.^579^ Because the management of CRS depends on the severity of the disease, several institutions had independently developed different CRS grading systems prior to the publication of consensus guidelines. These guidelines have contributed to the standardization of CRS management. Both direct targeting and non-specific immunosuppressive strategies to counteract overactive immune cells and elevated cytokine are used to control CRS in patients receiving CAR T-cell therapy. IL-6 has been implicated as an activating signal for CAR-T cells and is considered a pivotal mediator of CRS. The empirical testing of various blocking antibodies soon identified IL-6 as a critical driver of CRS. Tocilizumab, a monoclonal antibody that blocks signaling through the IL-6 receptor (IL-6R), became a cornerstone of CRS management.^564,580–583^ In general, patients with grade 1 CRS should be given broad-spectrum antibiotics along with supportive care. This may vary depending on the end-organ toxicities that are observed. Intravenous tocilizumab should be administered for a maximum of 4 doses to patients with grade ≥2 CRS. In cases of grade ≥3 CRS and in cases of grade 2 toxicity with sustained hypotension after anti-IL-6 therapy, the addition of corticosteroids should be considered.^518^ To prevent the progression of CRS, emergent intervention is warranted. However, other potential causes of the inflammatory response, including infection and malignant progression, should be ruled out. If there is no improvement in CRS after treatment with tocilizumab and steroids, an examination for infection should be performed and managed as necessary. In addition to siltuximab and anakinra, other agents may be considered for patients who are refractory to both tocilizumab and corticosteroids. These agents include the Janus-associated kinase inhibitor, cyclophosphamide, extracorporeal cytokine adsorption with continuous renal replacement therapy, intravenous IgG (IVIG) and anti-thymocyte globulin. Data in support of the use of any of these agents are mostly from anecdotal reports or small case series.

#### Neurotoxicity

Neurotoxicity is another adverse event that has been a concern in clinical trials of various immune effector cell therapies.^577,584,585^ Neurologic toxicity may occur concurrently with CRS. However, in some cases, neurologic toxicity may not occur simultaneously but may occur before or days after CRS. Like CRS rates, neurotoxicity incidence rates across clinical trials vary considerably. Neurologic toxicities are diverse and may include temporary working memory loss, delirium, seizures and rarely, acute cerebral edema.^564^ Neurotoxicity associated with CAR T-cell therapies has been referred to as immune effector cell-associated neurotoxicity syndrome (ICANS). It is characterized by a pathologic process involving the central nervous system following any immunotherapy that results in the activation or engagement of endogenous or infused T cells and other immune effector cells. The time to the onset of neurotoxicity is typically 4–10 days after the administration of CAR-T cells, with a duration of 14–17 days. For BCMA-directed CAR T-cell therapies, the duration may be somewhat shorter. CRS is considered to be a potent risk factor for ICANS and the severity of CRS is highly correlated with that of ICANS. The development of neurotoxicity is associated with a higher pre-treatment disease burden, a higher peak CAR T-cell expansion, a higher baseline inflammatory status, an earlier and higher elevation of pro-inflammatory cytokines in the blood and cerebrospinal fluid and the presence of pre-existing neurological comorbidities.^563^ Pro-inflammatory cytokines were accumulated in the cerebrospinal fluid during severe neurotoxicity, with a disproportionately high level of IL-6, IL-8, and MCP1, suggesting a production that is specific to the central nervous system.^563^ IL-1, derived from monocytes, has recently been highlighted as a key driver of neurotoxicity.^556^ Gust et al. also described the endothelial dysfunction and increased permeability of the blood-brain barrier (BBB) during neurotoxicity following adoptive immunotherapy with CD19 CAR-T cells, which may help to identify risk predictors for neurotoxicity.^585^ Increased BBB permeability may enable inflammatory cytokines and immune cells to migrate into the central nervous system and potentially contribute to inflammation of the nervous system.^585,586^ As in the case of CRS, the risk factors and the incidence of CRS are reported with variability between studies. CD19-directed CAR is more likely than BCMA-directed CAR to be accompanied by high-grade ICANS. The grade of ICANS determines the management of neurotoxicity. Consensus guidelines with recommended grading of ICANS have been issued by the American Society for Transplantation and Cellular Therapy (ASTCT). It’s recommended that clinicians use this scale to grade any CAR-T cell-related neurotoxicity.^587,588^ Along with careful monitoring and supportive care, corticosteroids are the cornerstone of ICANS management. Since tocilizumab may exacerbate ICANS,^575^ for patients with grade 1 CRS (fever only) and higher grade ICANS, corticosteroids alone may be preferred. The NCCN consensus panel does not recommend treating patients receiving CAR T-cell therapy for neurotoxicity with tocilizumab unless they have concurrent CRS.^518^

#### Hemophagocytic lymphohistiocytosis/macrophage activation syndrome

Hemophagocytic lymphohistiocytosis/macrophage activation syndrome (HLH/MAS) is regarded as a serious immunologic syndrome that is triggered by out-of-control immune activation, which includes the hyperactivation of macrophages and lymphocytes, increased production of pro-inflammatory cytokines, infiltration of lymphocytes and histiocytes into tissues and organs and multi-organ failure.^569,584,589,590^ In contrast to primary HLH/MAS, CAR T-cell therapy-induced HLH/MAS is thought to be a type of secondary HLH/MAS because it is initiated by an immune trigger.^558^ In a recent study, it was estimated that HLH/MAS occurs in 3.5% of the patients who receive CAR T-cell therapy.^591^ Nevertheless, the actual incidence of HLH/MAS has been disputed, in part because of the close overlap in symptoms between CRS and HLH/MAS. The definitive diagnosis of HLH/MAS after CAR T-cell therapy can be challenging as the clinical features and laboratory abnormalities overlap substantially with the CRS.^558,591^ The majority of patients with moderate-to-severe CRS exhibit the typical laboratory abnormalities of HLH/MAS, such as elevated levels of CRP, cytopenia, hyperferritinemia, hypofibrinogenemia, coagulopathy, and increased levels of several serum cytokines, in particular IL-6, INF-γ and GM-CSF.^478,569,584,589^ The clinical manifestations related to CAR T cell-induced HLH/MAS typically comprise fever, multi-organ dysfunction and central nervous system disorders and occasionally hepatosplenomegaly or hemophagocytosis in the bone marrow or other organs.^589,591^ Suppressing the overactive immune cells that are contributing to symptoms is the ultimate goal of clinical management of HLH/MAS. In some cases, resistance to tocilizumab may also lead to late-onset HLH/MAS-like lesions. Corticosteroids, anakinra, or intrathecal cytarabine should be considered in such cases.^584,589,590^ However, there is still a lack of data to support the use of such drugs in this setting.

#### Hypogammaglobulinemia

Hypogammaglobulinemia is another potential risk related to CAR T-cell therapy. Hypogammaglobulinemia has been reported in up to 53% of patients who have been treated with CAR-T cells in clinical studies.^592,593^ Hypogammaglobulinemia is a disorder that is characterized by decreased levels of antibodies in the blood and an increase in the risk of infection. Hypogammaglobulinemia is the consequence of an extremely small number of B cells or plasma cells, termed B cell aplasia or plasma cell aplasia, respectively.^593^ Even in patients in CR after CAR T-cell therapy, long-term hypogammaglobulinemia may still occur. The recommendations are made based on experts’ opinions, institution-specific experience and infection prevention approaches and strategies from other contexts due to the lack of randomized, controlled clinical trials for the treatment of hypogammaglobulinemia.^593^ Hypogammaglobulinemia can be controlled with either intravenous or subcutaneous immunoglobulin G, a product of fractionated blood derived from the pooled plasma of many individuals.^592–595^ Immunoglobulin offers broad protection from opportunistic infections because it contains antibodies against a variety of infectious agents.^592^

#### Cytopenia

Patients receiving CAR T-cell therapy are also at high risk for developing hematologic toxicities, particularly sustained cytopenia such as neutropenia, thrombocytopenia, anemia and/or leukopenia.^596–598^ Cytopenia may appear following CAR-T infusion and always presents at an early stage (<30 days), frequently for a prolonged period (30–90 days) and sometimes persists or appears at a late stage (>90 days).^597^ The onset and duration of cytopenia are often correlated with the severity of CRS and ICANS, the burden of the tumor, the number of prior therapies, baseline blood counts, peak levels of CRP and ferritin, as well as the CAR construct.^596,597,599,600^ Bone marrow biopsy is critical for the evaluation of both primary disease and secondary bone marrow neoplasm in patients with persistent or late-onset cytopenia. The management options for cytopenia are somewhat limited and need to be individualized based on the likely underlying etiology. These options may include growth factors, thrombopoietin receptor agonists, stem cell enhancement, transfusion support and the elimination of infectious risk.^597,601,602^

#### Coagulopathy

The typical time to onset of CAR-T therapy-associated coagulopathy (CARAC) is often 6 to 10 days after CAR-T cell infusion and closely follows the elevation of IL-6 and other cytokines and gradually relieves as the CRS is controlled.^503^ CARAC, including disseminated intravascular coagulation (DIC), prolonged prothrombin time/activated partial thromboplastin time, and hypofibrinogenemia, often occurs in patients with severe CRS.^603^ Over half of the patients experienced thrombocytopenia or at least one abnormal coagulation parameter after CAR-T therapy. Clinically bleeding events occurred in about 19.6% of patients with coagulopathy and 14 to 50% of patients with coagulopathy developed DIC; 6.7 to 42.9% of patients with DIC died.^503^ Monitoring of patients with CARAC is imperative to avoid the potential for bleeding events and even life-threatening hemorrhage. Since the severity of CARAC is highly associated with that of CRS, the management of CRS is of great importance. As bleeding is the main feature of CARAC, replacement therapy can decrease the risk of bleeding and control active bleeding, including the transfusion of platelet, fresh frozen plasma and prothrombin complex concentrates and fibrinogen, and cryoprecipitate. More importantly, anticoagulant therapy and/or antifibrinolytic therapy should be used as appropriate for patients with high-grade CRS.^503^

#### Infection

Infectious complications following CAR T-cell therapy are very common. They have been reported in up to ~70% of recipients.^604–606^ The majority of infections develop shortly following infusion and can be attributed to several causes, such as the depletion of normal B cells or plasma cells resulting from the direct action of the CAR-T cells, the depletion of lymphocytes and granulocytes caused by conditioning chemotherapy, anti-cytokine, or corticosteroid therapies given for CRS or neurologic toxicity and immunocompromise induced by the patient’s underlying malignancy.^604,607^ Infections, including bacterial, viral and fungal infections, have been reported following CAR T-cell therapy and can be life-threatening.^604^ An increased likelihood of acute infections may also be linked to the seriousness of CRS. The control of infections is generally with agents that are selective for the source of the infection. Risk stratification should be performed based on patient characteristics such as prior suppressive therapy, history of infection, etc. when determining whether to provide antibacterial or antifungal prophylaxis.^604^ For certain patients with concurrent severe or recurrent infections and hypogammaglobulinemia, the NCCN guidelines suggest IVIG as a replacement treatment.^518^

#### ADA

Since CAR is an exogenous sequence, it has certain immunogenicity which leads to ADA production by humoral immunity after infusion. Early-generation CAR-T cells were constructed from murine-derived scFv and the species difference resulted in the generation of a HAMA.^276^ Even though humanized CAR circumvented immunogenicity to a certain extent, some patients were still reported to have ADA in clinical trials of CD19 and BCMA, which eventually affected the efficacy or led to earlier relapse.^278,608^ Therefore, monitoring of ADA has become an important part of current CAR-T clinical trials. Although with large individual variability, the factors related to the production of ADA are currently thought to be the use of CAR-T with murine scFv and multiple infusions of the same CAR-T product.^275,609^ There is no targeted method to solve the problem of ADA, but it should be monitored by ELISA (enzyme-linked immunosorbent assay) and flow cytometry, to understand the reason for drug resistance or recurrence in patients in time,^610,611^ and to reduce the impact of ADA on secondary reinfusion by replacing the target and strengthening lymphodepletion.^609,612^

## Challenges and future prospects

Each immunotherapy strategy has achieved varying degrees of encouraging results in hematologic malignancies. Different immunotherapeutic approaches have their advantages but also shortcomings that need to be addressed (Table 5). Further clinical exploration will be needed to further improve the prognosis of patients with hematologic malignancies. The allo-HSCT remains the primary treatment for hematologic malignancies with a potentially curative outcome. The haplo-HSCT modality can best address the limited source of allo-HSCT donors, but it’s still necessary to further explore how to minimize the severity of GVHD and transplant-related death while improving anti-tumor effects, especially for patients with R/R hematologic cancers. The future direction of transplantation will be toward personalization, in which a combination therapy strategy is very essential. R/R patients can be pre-treated with CAR-T therapy or other targeted therapies to achieve remission before bridging to HSCT. Patients, who still have residual disease after incomplete remission with various treatments such as chemotherapy, targeted therapy and immunotherapy, can be treated with donor CAR-T combined with allo-HSCT. For patients with positive MRD after transplantation, CAR-T therapy can also be recommended. The second direction is to optimize donor selection, especially for familial donors and to avoid selecting donors who carry the same genetic defect as the patient. Molecular testing can be used to detect HLA loss and guide the search for donors for patients who need a second transplantation. In addition, anti-tumor therapy needs to be considered along with GVHD prevention and thus individualized management should be conducted after transplantation to balance the anti-GVHD and anti-tumor benefits. The mAbs, bsAbs, and ADC-based agents have also improved the treatment of cancer patients to some extent, but the clinical toxicities remain unavoidable. Meanwhile, some patients have demonstrated little or no responsiveness to such treatments. Ideal tumor antigens need to be screened for these antibody-based therapies to improve the anti-tumor effects and reduce the incidence of “off-tumor, on-target” effects. The technical threshold for the development of bsAbs is more difficult compared to single-target mAbs. Selecting the best target combination is only the first step, followed by a rational structural design based on the receptor structure as well as the biological mechanism of the disease. In addition, inappropriate clinical design and dosing regimens will result in higher toxicity in patients, which can be improved by optimizing treatment strategy, dose and timing to reduce side effects to some extent. The payload and linker in ADC drugs can also directly affect effectiveness and safety. In addition, how to solve the complexity of pharmacokinetics, enhance drug stability, improve drug efficacy and reduce drug resistance are also urgent to be explored. The bsADC (bispecific antibody-drug conjugate) combines the advantages of bsAbs and ADCs and is a major challenge for the future. Compared to mAbs, bsADCs can target tumor cells more specifically through two antibodies, overcoming drug resistance while increasing the safety. Meanwhile, novel therapeutic agents, such as bifunctional checkpoint-inhibitory T cell engager (CiTE),^613^ simultaneous multiple interaction T cell engager (SMITE),^614^ trispecific killer engager (TriKE) and BiTE-expressing CAR-T cells, are being designed to integrate various immune functions into one molecule or a single cellular vector and thereby enhance efficacy without compromising safety.^172^ ICIs have shown superior efficacy mainly in HL and primary mediastinal large B-cell lymphoma, but has limited efficacy in other hematologic cancers. Serious irAEs may also occur with ICI therapy, which will impede its application in the clinic. The exploration of more-effective and rational combinatorial approaches is an area of great interest in improving the efficacy of ICI therapy. The emergence of ACTs, especially CAR T-cell therapy, offers a new therapeutic avenue and hope for R/R patients with hematologic malignancies. However, these therapeutic approaches are usually accompanied by serious complications such as CRS, ICANS, and “off-target” effects, while achieving remarkable results. Challenges remain in the optimization of CAR design and cell products, improvement of remission rates, prolongation of remission duration, reduction of toxicity and expansion of this therapeutic modality to other cancer types. To further improve patient outcomes, innovative strategies are needed to enhance the therapeutic efficacy and in vivo persistence of CAR-T cells and to mitigate tumor cell resistance. Elucidation of mechanisms of resistance and immune escape has long been a big challenge. Epigenetic mechanisms play an important role in both tumor development and anti-tumor immune regulation and epi-drugs represented by DNA methylation inhibitors and histone deacetylation inhibitors can coordinate, potentiate and reduce immune escape effects in several aspects by regulating tumor killing and enhancing the anti-tumor immunity.^615–617^ Therefore, a deeper and broader exploration of epi-immunotherapy will further advance the understanding of this emerging concept and bring more creative breakthroughs in immunotherapy. Allogeneic CAR-T cells also have the potential to overcome many of the manufacturing limitations of traditional autologous CAR T-cell therapies. Universal CAR-T cells will undoubtedly be the future direction of CAR-T therapy. While there are still concerns about host-versus-graft and graft-versus-host reactions caused by CAR-T cells in the allogeneic environment, the risks and side effects are being reduced through gene knockout technology and the safety of universal CAR-T cells will be further enhanced. Universal CAR-T therapies are expected to bring less expensive and more immediately available “off-the-shelf” therapies to patients with malignant hematologic cancers. However, there are many challenges with universal CAR-T cells and clinical studies are still in the early stages. Tumor vaccines take advantage of tumor-associated antigens or tumor-specific antigens to stimulate the immune system but are currently in their infant stage and there is still much space for refinement to discover their full potential.

**Table 5:** The advantages and limitations of various immunotherapies in hematologic malignancies

Type of immunotherapy	Advantages	Limitations	Future directions
allo-HSCT	The only option to achieve a cure for hematologic malignancies.	Incidence of transplant related mortality and graft-versus-host disease.	Personalization and combination therapy; optimization of donor selection, maintenance therapy to balance the anti-GVHD and anti-tumor benefits.
mAb	Specifically targeting tumor antigen and inducing cancer cell death; their combination with chemotherapy has been first-line therapy for several cancers.	Incidence of “off tumor, on target” effect and therapy-related toxicities.	Requirement for suitable target antigen; optimization of treatment strategy; overcome drug resistance to single-agent therapies.
bsAb	Combining the binding sites of two monoclonal antibodies in the same one molecule to promote cancer cell killing.	Incidence of “off tumor, on target” effect and therapy-related toxicities; a lack of co-stimulation might induce T-cell anergy and compromise the clinical efficacy;	Requirement for suitable target antigen; need to selecting the best target combination; require rational structural design; optimization of treatment strategy; overcome drug resistance to single-agent therapies.
ADC	Utilizing the specific binding properties of mAb to selectively deliver cytotoxic agents to cancer cells to increase the therapeutic potentials of cytotoxic agents.	Incidence of “off tumor, on target” effect and therapy-related toxicities.	Requirement for suitable target antigen; require rational structural design; solve the complexity of pharmacokinetics, enhance drug stability, improve drug efficacy and reduce drug resistance; optimization of treatment strategy; design of bsADCs; overcome drug resistance to single-agent therapies.
ICI	Blockade of immunosuppressive checkpoint signaling pathway.	Incidence of irAEs; only the therapeutic results in HL was remarkable.	Overcome drug resistance to single-agent therapies; combination therapy with epi-drugs, CAR-T therapy and/or HSCT.
CIK, γδ T and NK cells	Non-specific cellular therapies; no demand for genetical modification.	Requirement for a large number of cells; limited efficacy in hematologic malignancies.	Improvement of clinical efficacy and reduction of toxicity; combination therapy with epi-drugs, ICIs and/or HSCT.
CAR-T cell therapy	Specific cellular therapies; no restriction of MHC; achieve rapid development and great success in treating hematologic malignancies, especially R/R patients; serve as the “bridge” to transplant; several cell products have achieved FDA’s approval and entered into the commercialized field.	Therapy-related toxicities, such as CRS and neurotoxicity; long period of manufacturing; high cost.	Requirement for suitable target antigen; optimization of CAR design and cell products; improvement of remission rates; prolongation of remission duration; reduction of toxicity and expansion of this therapeutic modality to other cancer types; universal CAR-T products; overcome drug resistance to monotherapy; combination therapy with epi-drugs, ICIs and/or HSCT.
CAR-NK cell therapy	Specific cellular therapies; no restriction of MHC; provide an “off-the-shelf” cell product and could be readily available for immediate clinical use; serve as the “bridge” to transplant.	Still in early stage of clinical studies; limited efficacy in hematologic malignancies.	Requirement for suitable target antigen; optimization of CAR design and cell products; improvement of clinical efficacy and reduction of toxicity; expansion of this therapeutic modality to other cancer types; overcome drug resistance to monotherapy; combination therapy with epi-drugs, ICIs and/or HSCT.
Tumor vaccine	Taking advantage of tumor-associated antigens or tumor-specific antigens to stimulate the immune system.	Still in very early stage of clinical study; limited efficacy in hematologic malignancies.	Requirement for suitable target antigen and vaccine vectors; improvement of clinical efficacy and reduction of toxicity.

*allo-HSCT* allogeneic hematopoietic stem cell transplantation, *mAb* monoclonal antibody, *bsAb* bispecific antibody, *ADC* antibody-drug conjugate, *bsADC* bispecific antibody-drug conjugate, *ICI* immune checkpoint inhibitor, *HL* Hodgkin lymphoma, *irAEs* immune-related adverse effects, *MHC* major histocompatibility complex, *R/R* refractory and relapsed, *FDA* Food and Drug Administration, *CIK* cytokine-induced killer cells, *γδ* T gamma/delta T, *NK* natural killer, *CAR-T* chimeric antigen receptor T, *CRS* cytokine release syndrome

The current status quo in cancer treatment is that immunotherapy is generally used as a second-, third-, or even last-line treatment option when patients have no better options. Based on promising results in terms of efficacy and safety, immunotherapy is expected to become the first line of treatment in the future, while conventional treatment will be relegated to the second line.^618^ Treatment regimens for patients with hematologic cancers typically include 3–4 or even 5 cytotoxic drugs and the addition of immunotherapy drugs can reduce the use of these chemotherapy agents. Several clinical trials have confirmed that the combination of immunotherapy with reduced chemotherapy regimens has improved rather than suppressed therapeutic effects. Therefore, one of the major trends in cancer treatment is that immunotherapy will become increasingly prominent.^618^ Combination immunotherapy is an exciting area of research that may further enhance our ability to utilize the immune system against hematologic malignancies. Currently, HSCT remains a fundamental treatment option and combining HSCT with novel immunotherapies is a promising direction for our future. Many clinical questions remain to be answered. Which immunotherapy works best in the context of HSCT? Which immunotherapy is better suited as a bridge to HSCT or as a preferred option after HSCT relapse? Which immunotherapy approach is more appropriate for patients who are ineligible for HSCT? With the continuous development and advancement of molecular biology and immunology technologies, immunotherapy is expected to further change the existing treatment paradigm of hematological cancers. The detailed information generated by multidimensional omics technologies, single-cell sequencing and others will not only provide insights into the complex determinants of efficacy and toxicity of immunotherapies but also help identify predictive biomarkers and develop new treatment strategies. As future research helps to address these challenges, these advanced technologies may eventually become the standard and necessary tool in the field of immunotherapy, revealing the relationship between key drivers of cancer phenotypes and enabling clinicians to better predict and monitor patient responses, thereby facilitating more comprehensive and realistic personalized treatments for cancer patients.^619–632^

## Conclusion

Malignant hematologic cancers are major diseases that pose a serious threat to human health. The past and present are very exciting eras for immunotherapy of hematologic malignancies, but the future looks quite incredible and we are rapidly moving in that direction. Although the various immunotherapies aim to treat cancer patients through different mechanisms of action, the core is to restart and maintain the “Cancer-Immunity Cycle” and restore normal anti-tumor immunity. Multiple categories of immunotherapies have been developed for the treatment of blood cancers and are being further evaluated in clinical trials. More importantly, some of these immunotherapies have been approved by the FDA for the treatment of blood cancers or have even entered the commercialization stage. At present, immunotherapy for blood cancers still faces a series of challenges. The most important of these is safety, where different therapies are accompanied by varying degrees of treatment-related side effects, thus emphasizing the importance of early detection and intervention of toxicities. As mentioned above, clinical experts have been developing guidelines for the management of toxicities based on clinical trials and real-world clinical experience. The establishment of these guidelines has provided a solid foundation for improving the safety and widespread use of immunotherapy. In addition, they are gaining experience in managing the unique complications associated with novel immunotherapies and establishing practice guidelines that will be critical to expanding their use worldwide. Another notable issue is treatment failure due to resistance and relapse. This illustrates the striking difference in the ability of each patient to respond to immunotherapy, highlighting the potentially urgent need for and importance of personalized cancer treatment.

## References

[1] Koebel CM (2007). Adaptive immunity maintains occult cancer in an equilibrium state. Nature.

[2] von Locquenghien, M., Rozalén, C. & Celià-Terrassa, T. Interferons in cancer immunoediting: sculpting metastasis and immunotherapy response. *J. Clin. Invest*. **131**, e143296 (2021). 10.1172/JCI143296 PMC7773346 33393507

[3] Vinay DS (2015). Immune evasion in cancer: mechanistic basis and therapeutic strategies. Semin. Cancer Biol..

[4] Zitvogel L, Tesniere A, Kroemer G (2006). Cancer despite immunosurveillance: immunoselection and immunosubversion. Nat. Rev. Immunol..

[5] Dunn GP, Old LJ, Schreiber RD (2004). The immunobiology of cancer immunosurveillance and immunoediting. Immunity.

[6] Chen DS, Mellman I (2013). Oncology meets immunology: the cancer-immunity cycle. Immunity.

[7] Motz GT, Coukos G (2013). Deciphering and reversing tumor immune suppression. Immunity.

[8] McNutt M (2013). Cancer immunotherapy. Science.

[9] Couzin-Frankel J (2013). Breakthrough of the year 2013. Cancer immunotherapy. Science.

[10] Couzin J (2002). Cancer immunotherapy. select T cells, given space, shrink tumors. Science.

[11] Pui CH, Evans WE (1998). Acute lymphoblastic leukemia. N. Engl. J. Med..

[12] Döhner H, Weisdorf DJ, Bloomfield CD (2015). Acute myeloid leukemia. N. Engl. J. Med..

[13] Kayser S, Levis MJ (2023). The clinical impact of the molecular landscape of acute myeloid leukemia. Haematologica.

[14] Armitage JO, Gascoyne RD, Lunning MA, Cavalli F (2017). Non-Hodgkin lymphoma. Lancet.

[15] Wang HW, Balakrishna JP, Pittaluga S, Jaffe ES (2019). Diagnosis of hodgkin lymphoma in the modern era. Br. J. Haematol..

[16] Ansell SM (2015). Hodgkin lymphoma: diagnosis and treatment. Mayo Clin. Proc..

[17] van de Donk N, Pawlyn C, Yong KL (2021). Multiple myeloma. Lancet.

[18] Kennedy JA, Ebert BL (2017). Clinical implications of genetic mutations in myelodysplastic syndrome. J. Clin. Oncol..

[19] Bachireddy P, Burkhardt UE, Rajasagi M, Wu CJ (2015). Haematological malignancies: at the forefront of immunotherapeutic innovation. Nat. Rev. Cancer.

[20] Im A, Pavletic SZ (2017). Immunotherapy in hematologic malignancies: past, present, and future. J. Hematol. Oncol..

[21] Salles G (2017). Rituximab in B-cell hematologic malignancies: a review of 20 years of clinical experience. Adv. Ther..

[22] Tilly H (2015). Diffuse large B-cell lymphoma (DLBCL): ESMO clinical practice guidelines for diagnosis, treatment and follow-up. Ann. Oncol..

[23] Salles G (2020). Tafasitamab plus lenalidomide in relapsed or refractory diffuse large B-cell lymphoma (L-MIND): a multicentre, prospective, single-arm, phase 2 study. Lancet Oncol..

[24] Palumbo A (2016). Daratumumab, bortezomib, and dexamethasone for multiple myeloma. N. Engl. J. Med..

[25] Feldman EJ (2005). Phase III randomized multicenter study of a humanized anti-CD33 monoclonal antibody, lintuzumab, in combination with chemotherapy, versus chemotherapy alone in patients with refractory or first-relapsed acute myeloid leukemia. J. Clin. Oncol..

[26] Topp MS (2015). Safety and activity of blinatumomab for adult patients with relapsed or refractory B-precursor acute lymphoblastic leukaemia: a multicentre, single-arm, phase 2 study. Lancet Oncol..

[27] Connors JM (2018). Brentuximab vedotin with chemotherapy for stage III or IV Hodgkin’s lymphoma. N. Engl. J. Med..

[28] Horwitz S (2019). Brentuximab vedotin with chemotherapy for CD30-positive peripheral T-cell lymphoma (ECHELON-2): a global, double-blind, randomised, phase 3 trial. Lancet.

[29] Oka Y (2003). Wilms tumor gene peptide-based immunotherapy for patients with overt leukemia from myelodysplastic syndrome (MDS) or MDS with myelofibrosis. Int. J. Hematol..

[30] Oka Y (2004). Induction of WT1 (Wilms’ tumor gene)-specific cytotoxic T lymphocytes by WT1 peptide vaccine and the resultant cancer regression. Proc. Natl Acad. Sci. USA.

[31] Xu-Monette ZY, Zhou J, Young KH (2018). PD-1 expression and clinical PD-1 blockade in B-cell lymphomas. Blood.

[32] Bagchi S, Yuan R, Engleman EG (2021). Immune checkpoint inhibitors for the treatment of cancer: clinical impact and mechanisms of response and resistance. Annu Rev. Pathol..

[33] Sebestyen Z (2020). Translating gammadelta (γδ) T cells and their receptors into cancer cell therapies. Nat. Rev. Drug Discov..

[34] Wang W, Jiang J, Wu C (2020). CAR-NK for tumor immunotherapy: clinical transformation and future prospects. Cancer Lett..

[35] Xu Z, Huang X (2021). Cellular immunotherapy for hematological malignancy: recent progress and future perspectives. Cancer Biol. Med..

[36] Melenhorst JJ (2022). Decade-long leukaemia remissions with persistence of CD4(+) CAR T cells. Nature.

[37] Holstein SA, Lunning MA (2020). CAR T-cell therapy in hematologic malignancies: a voyage in progress. Clin. Pharmacol. Ther..

[38] Holtick, U. et al. Bone marrow versus peripheral blood allogeneic haematopoietic stem cell transplantation for haematological malignancies in adults. *Cochrane Database Syst. Rev*. Cd010189 (2014). 10.1002/14651858.CD010189.pub2 PMC10612998 24748537

[39] Penack O (2020). Prophylaxis and management of graft versus host disease after stem-cell transplantation for haematological malignancies: updated consensus recommendations of the European Society for Blood and Marrow Transplantation. Lancet Haematol..

[40] Du J (2021). Comparison of allogeneic stem cell transplant and autologous stem cell transplant in refractory or relapsed peripheral T-cell lymphoma: a systematic review and meta-analysis. JAMA Netw. Open..

[41] Cornelissen JJ, Blaise D (2016). Hematopoietic stem cell transplantation for patients with AML in first complete remission. Blood.

[42] Gagelmann N (2022). Reduced intensity hematopoietic stem cell transplantation for accelerated-phase myelofibrosis. Blood Adv..

[43] Kato K, Khaled Y, Mineishi S (2007). Reduced-intensity stem cell transplantation for hematological malignancies: current status and the future. Curr. Stem Cell Res. Ther..

[44] Cohen S (2020). Hematopoietic stem cell transplantation using single UM171-expanded cord blood: a single-arm, phase 1-2 safety and feasibility study. Lancet Haematol..

[45] Huang XJ (2004). Combined transplantation of G-CSF primed allogeneic bone marrow cells and peripheral blood stem cells in treatment of severe aplastic anemia. Chin. Med. J..

[46] Luznik L (2008). HLA-haploidentical bone marrow transplantation for hematologic malignancies using nonmyeloablative conditioning and high-dose, posttransplantation cyclophosphamide. Biol. Blood Marrow Transpl..

[47] Kanakry CG, Fuchs EJ, Luznik L (2016). Modern approaches to HLA-haploidentical blood or marrow transplantation. Nat. Rev. Clin. Oncol..

[48] Chang YJ (2016). Controlled, randomized, open-label trial of risk-stratified corticosteroid prevention of acute graft-versus-host disease after haploidentical transplantation. J. Clin. Oncol..

[49] Gooley TA (2010). Reduced mortality after allogeneic hematopoietic-cell transplantation. N. Engl. J. Med..

[50] Kanakry CG, Fuchs EJ, Luznik L (2016). Modern approaches to HLA-haploidentical blood or marrow transplantation. Nat. Rev. Clin. Oncol..

[51] Ciurea SO (2015). Haploidentical transplant with posttransplant cyclophosphamide vs matched unrelated donor transplant for acute myeloid leukemia. Blood.

[52] Tomita M, Tsumoto K (2011). Hybridoma technologies for antibody production. Immunotherapy.

[53] Buist MR, Kenemans P, van Kamp GJ, Haisma HJ (1995). Minor human antibody response to a mouse and chimeric monoclonal antibody after a single i.v. infusion in ovarian carcinoma patients: a comparison of five assays. Cancer Immunol. Immunother..

[54] Klee GG (2000). Human anti-mouse antibodies. Arch. Pathol. Lab. Med..

[55] Legouffe E (1994). Human anti-mouse antibody response to the injection of murine monoclonal antibodies against IL-6. Clin. Exp. Immunol..

[56] Arakawa F (1996). Cloning and sequencing of the VH and V kappa genes of an anti-CD3 monoclonal antibody, and construction of a mouse/human chimeric antibody. J. Biochem..

[57] Nishimura Y (1987). Recombinant human-mouse chimeric monoclonal antibody specific for common acute lymphocytic leukemia antigen. Cancer Res..

[58] Smith GP (1985). Filamentous fusion phage: novel expression vectors that display cloned antigens on the virion surface. Science.

[59] Saw PE, Song EW (2019). Phage display screening of therapeutic peptide for cancer targeting and therapy. Protein Cell.

[60] Chao G (2006). Isolating and engineering human antibodies using yeast surface display. Nat. Protoc..

[61] Boder ET, Wittrup KD (1997). Yeast surface display for screening combinatorial polypeptide libraries. Nat. Biotechnol..

[62] Glukhova XA (2016). Updates on the production of therapeutic antibodies using human hybridoma technique. Curr. Pharm. Des..

[63] Buettner MJ (2018). Improving immunotherapy through glycodesign. Front. Immunol..

[64] Tsao LC, Force J, Hartman ZC (2021). Mechanisms of therapeutic antitumor monoclonal antibodies. Cancer Res..

[65] Mossner E (2010). Increasing the efficacy of CD20 antibody therapy through the engineering of a new type II anti-CD20 antibody with enhanced direct and immune effector cell-mediated B-cell cytotoxicity. Blood.

[66] Tipton TR (2015). Anti-mouse FcγRIV antibody 9E9 also blocks FcγRIII in vivo. Blood.

[67] Herter S (2013). Preclinical activity of the type II CD20 antibody GA101 (obinutuzumab) compared with rituximab and ofatumumab In vitro and in xenograft models. Mol. Cancer Ther..

[68] Overdijk MB (2015). Antibody-mediated phagocytosis contributes to the anti-tumor activity of the therapeutic antibody daratumumab in lymphoma and multiple myeloma. MAbs.

[69] Krejcik J (2016). Daratumumab depletes CD38(+) immune regulatory cells, promotes T-cell expansion, and skews T-cell repertoire in multiple myeloma. Blood.

[70] Moreno L (2019). The mechanism of action of the anti-CD38 monoclonal antibody isatuximab in multiple myeloma. Clin. Cancer Res..

[71] Wierda WG (2010). Ofatumumab as single-agent CD20 immunotherapy in fludarabine-refractory chronic lymphocytic leukemia. J. Clin. Oncol..

[72] Hillmen P (2015). Chlorambucil plus ofatumumab versus chlorambucil alone in previously untreated patients with chronic lymphocytic leukaemia (COMPLEMENT 1): a randomised, multicentre, open-label phase 3 trial. Lancet.

[73] Goede V (2014). Obinutuzumab plus chlorambucil in patients with CLL and coexisting conditions. N. Engl. J. Med..

[74] Sehn LH (2016). Obinutuzumab plus bendamustine versus bendamustine monotherapy in patients with rituximab-refractory indolent non-Hodgkin lymphoma (GADOLIN): a randomised, controlled, open-label, multicentre, phase 3 trial. Lancet Oncol..

[75] Dimopoulos MA (2016). Daratumumab, lenalidomide, and dexamethasone for multiple myeloma. N. Engl. J. Med..

[76] Lokhorst HM (2015). Targeting CD38 with daratumumab monotherapy in multiple myeloma. N. Engl. J. Med..

[77] Lonial S (2016). Daratumumab monotherapy in patients with treatment-refractory multiple myeloma (SIRIUS): an open-label, randomised, phase 2 trial. Lancet.

[78] Mateos MV (2018). Daratumumab plus bortezomib, melphalan, and prednisone for untreated myeloma. N. Engl. J. Med..

[79] Lonial S (2015). Elotuzumab therapy for relapsed or refractory multiple myeloma. N. Engl. J. Med..

[80] Dimopoulos MA (2022). Addition of elotuzumab to lenalidomide and dexamethasone for patients with newly diagnosed, transplantation ineligible multiple myeloma (ELOQUENT-1): an open-label, multicentre, randomised, phase 3 trial. Lancet Haematol..

[81] Dimopoulos MA (2023). Elotuzumab plus pomalidomide and dexamethasone for relapsed/refractory multiple myeloma: final overall survival analysis from the randomized phase II ELOQUENT-3 trial. J. Clin. Oncol..

[82] Moreau P (2021). Isatuximab, carfilzomib, and dexamethasone in relapsed multiple myeloma (IKEMA): a multicentre, open-label, randomised phase 3 trial. Lancet.

[83] Attal M (2019). Isatuximab plus pomalidomide and low-dose dexamethasone versus pomalidomide and low-dose dexamethasone in patients with relapsed and refractory multiple myeloma (ICARIA-MM): a randomised, multicentre, open-label, phase 3 study. Lancet.

[84] Goldschmidt H (2022). Addition of isatuximab to lenalidomide, bortezomib, and dexamethasone as induction therapy for newly diagnosed, transplantation-eligible patients with multiple myeloma (GMMG-HD7): part 1 of an open-label, multicentre, randomised, active-controlled, phase 3 trial. Lancet Haematol..

[85] Coiffier B (1998). Rituximab (anti-CD20 monoclonal antibody) for the treatment of patients with relapsing or refractory aggressive lymphoma: a multicenter phase II study. Blood.

[86] Vose JM (2001). Phase II study of rituximab in combination with chop chemotherapy in patients with previously untreated, aggressive non-Hodgkin’s lymphoma. J. Clin. Oncol..

[87] Salles G (2011). Rituximab maintenance for 2 years in patients with high tumour burden follicular lymphoma responding to rituximab plus chemotherapy (PRIMA): a phase 3, randomised controlled trial. Lancet.

[88] Seymour JF (2018). Venetoclax-rituximab in relapsed or refractory chronic lymphocytic leukemia. N. Engl. J. Med..

[89] Morschhauser F (2013). 90Yttrium-ibritumomab tiuxetan consolidation of first remission in advanced-stage follicular non-Hodgkin lymphoma: updated results after a median follow-up of 7.3 years from the International, Randomized, Phase III First-LineIndolent trial. J. Clin. Oncol..

[90] Coiffier B (2008). Safety and efficacy of ofatumumab, a fully human monoclonal anti-CD20 antibody, in patients with relapsed or refractory B-cell chronic lymphocytic leukemia: a phase 1-2 study. Blood.

[91] Morschhauser F (2009). Humanized anti-CD20 antibody, veltuzumab, in refractory/recurrent non-Hodgkin’s lymphoma: phase I/II results. J. Clin. Oncol..

[92] Morschhauser FA (2013). Obinutuzumab (GA101) monotherapy in relapsed/refractory diffuse large b-cell lymphoma or mantle-cell lymphoma: results from the phase II GAUGUIN study. J. Clin. Oncol..

[93] Radford J (2013). Obinutuzumab (GA101) plus CHOP or FC in relapsed/refractory follicular lymphoma: results of the GAUDI study (BO21000). Blood.

[94] Salles G (2012). Phase 1 study results of the type II glycoengineered humanized anti-CD20 monoclonal antibody obinutuzumab (GA101) in B-cell lymphoma patients. Blood.

[95] Salles GA (2013). Obinutuzumab (GA101) in patients with relapsed/refractory indolent non-Hodgkin lymphoma: results from the phase II GAUGUIN study. J. Clin. Oncol..

[96] Morschhauser F (2010). Results of a phase I/II study of ocrelizumab, a fully humanized anti-CD20 mAb, in patients with relapsed/refractory follicular lymphoma. Ann. Oncol..

[97] Forero-Torres A (2012). Results of a phase 1 study of AME-133v (LY2469298), an Fc-engineered humanized monoclonal anti-CD20 antibody, in FcγRIIIa-genotyped patients with previously treated follicular lymphoma. Clin. Cancer Res..

[98] Ganjoo KN (2015). Phase 1/2 study of ocaratuzumab, an Fc-engineered humanized anti-CD20 monoclonal antibody, in low-affinity FcγRIIIa patients with previously treated follicular lymphoma. Leuk. Lymphoma.

[99] Cheney CM (2014). Ocaratuzumab, an Fc- engineered antibody demonstrates enhanced antibody- dependent cell- mediated cytotoxicity in chronic lymphocytic leukemia. Mabs.

[100] Wulf GG (2021). Alemtuzumab plus CHOP versus CHOP in elderly patients with peripheral T-cell lymphoma: the DSHNHL2006-1B/ACT-2 trial. Leukemia.

[101] Cortelezzi A (2009). Low-dose subcutaneous alemtuzumab in refractory chronic lymphocytic leukaemia (CLL): results of a prospective, single-arm multicentre study. Leukemia.

[102] Ansell S (2003). Phase I/II study of a fully human anti-CD30 monoclonal antibody (MDX-060) in Hodgkin’s disease (HD) and anaplastic large cell lymphoma (ALCL). Blood.

[103] Ansell SM (2004). Phase I/II, open-label, dose-escalating study of MDX-060 administered weekly for 4 weeks in subjects with refractory/relapsed CD30 positive lymphoma. Blood.

[104] Hussein M (2010). A phase I multidose study of dacetuzumab (SGN-40; humanized anti-CD40 monoclonal antibody) in patients with multiple myeloma. Haematologica.

[105] Advani R (2009). Phase I study of the humanized anti-CD40 monoclonal antibody dacetuzumab in refractory or recurrent non-Hodgkin’s lymphoma. J. Clin. Oncol..

[106] Fayad L (2015). Dacetuzumab plus rituximab, ifosfamide, carboplatin and etoposide as salvage therapy for patients with diffuse large B-cell lymphoma relapsing after rituximab, cyclophosphamide, doxorubicin, vincristine and prednisolone: a randomized, double-blind, placebo-controlled phase 2b trial. Leuk. Lymphoma.

[107] Stein R (2009). Combining milatuzumab with bortezomib, doxorubicin, or dexamethasone improves responses in multiple myeloma cell lines. Clin. Cancer Res..

[108] Alinari L (2011). Combination anti-CD74 (milatuzumab) and anti-CD20 (rituximab) monoclonal antibody therapy has in vitro and in vivo activity in mantle cell lymphoma. Blood.

[109] Smith MR, Jin F, Joshi I (2014). Milatuzumab and veltuzumab induce apoptosis through JNK signalling in an NF-κB dependent human transformed follicular lymphoma cell line. Br. J. Haematol..

[110] Hertlein E (2010). Milatuzumab immunoliposomes induce cell death in CLL by promoting accumulation of CD74 on the surface of B cells. Blood.

[111] Vasu S (2022). A phase I study of the fully human, fragment crystallizable-engineered, anti-CD-33 monoclonal antibody BI 836858 in patients with previously-treated acute myeloid leukemia. Haematologica.

[112] Ohmachi K (2019). A multicenter phase I study of inebilizumab, a humanized anti-CD19 monoclonal antibody, in Japanese patients with relapsed or refractory B-cell lymphoma and multiple myeloma. Int. J. Hematol..

[113] Czuczman MS (2018). Phase II trial of galiximab (anti-CD80 monoclonal antibody) plus rituximab (CALGB 50402): Follicular Lymphoma International Prognostic Index (FLIPI) score is predictive of upfront immunotherapy responsiveness. Ann. Oncol..

[114] Leonard JP (2005). Combination antibody therapy with epratuzumab and rituximab in relapsed or refractory non-Hodgkin’s lymphoma. J. Clin. Oncol..

[115] Hicklin DJ, Ellis LM (2005). Role of the vascular endothelial growth factor pathway in tumor growth and angiogenesis. J. Clin. Oncol..

[116] Viardot A, Bargou R (2018). Bispecific antibodies in haematological malignancies. Cancer Treat. Rev..

[117] Tian Z, Liu M, Zhang Y, Wang X (2021). Bispecific T cell engagers: an emerging therapy for management of hematologic malignancies. J. Hematol. Oncol..

[118] Wang Z (2021). Bispecific antibody-activated T cells enhance NK cell-mediated antibody-dependent cellular cytotoxicity. J. Hematol. Oncol..

[119] Suurs FV, Lub-de Hooge MN, de Vries EGE, de Groot DJA (2019). A review of bispecific antibodies and antibody constructs in oncology and clinical challenges. Pharmacol. Ther..

[120] Velasquez MP, Bonifant CL, Gottschalk S (2018). Redirecting T cells to hematological malignancies with bispecific antibodies. Blood.

[121] Li H, Er Saw P, Song E (2020). Challenges and strategies for next-generation bispecific antibody-based antitumor therapeutics. Cell. Mol. Immunol..

[122] Nagorsen D, Kufer P, Baeuerle PA, Bargou R (2012). Blinatumomab: a historical perspective. Pharmacol. Ther..

[123] Kantarjian H (2017). Blinatumomab versus chemotherapy for advanced acute lymphoblastic leukemia. N. Engl. J. Med..

[124] Advani AS (2022). SWOG 1318: a phase II trial of blinatumomab followed by POMP maintenance in older patients with newly diagnosed Philadelphia chromosome-negative B-cell acute lymphoblastic leukemia. J. Clin. Oncol..

[125] Goebeler ME (2016). Bispecific T-cell engager (BiTE) antibody construct blinatumomab for the treatment of patients with relapsed/refractory non-Hodgkin lymphoma: final results from a phase I study. J. Clin. Oncol..

[126] Viardot A (2016). Phase 2 study of the bispecific T-cell engager (BiTE) antibody blinatumomab in relapsed/refractory diffuse large B-cell lymphoma. Blood.

[127] Reusch U (2015). A tetravalent bispecific TandAb (CD19/CD3), AFM11, efficiently recruits T cells for the potent lysis of CD19(+) tumor cells. MAbs.

[128] Falchi L, Vardhana SA, Salles GA (2023). Bispecific antibodies for the treatment of B-cell lymphoma: promises, unknowns, and opportunities. Blood.

[129] Sun LL (2015). Anti-CD20/CD3 T cell-dependent bispecific antibody for the treatment of B cell malignancies. Sci. Transl. Med..

[130] Bock AM, Nowakowski GS, Wang Y (2022). Bispecific antibodies for non-Hodgkin lymphoma treatment. Curr. Treat. Options Oncol..

[131] Budde LE (2022). Safety and efficacy of mosunetuzumab, a bispecific antibody, in patients with relapsed or refractory follicular lymphoma: a single-arm, multicentre, phase 2 study. Lancet Oncol..

[132] Hutchings M (2021). Glofitamab, a novel, bivalent CD20-targeting T-cell-engaging bispecific antibody, induces durable complete remissions in relapsed or refractory B-cell lymphoma: a phase I trial. J. Clin. Oncol..

[133] Dickinson MJ (2022). Glofitamab for relapsed or refractory diffuse large B-cell lymphoma. N. Engl. J. Med..

[134] Hutchings M (2021). Dose escalation of subcutaneous epcoritamab in patients with relapsed or refractory B-cell non-Hodgkin lymphoma: an open-label, phase 1/2 study. Lancet.

[135] Thieblemont C (2023). Epcoritamab, a novel, subcutaneous CD3xCD20 bispecific T-cell-engaging antibody, in relapsed or refractory large B-cell lymphoma: dose expansion in a phase I/II trial. J. Clin. Oncol..

[136] Bannerji R (2022). Odronextamab, a human CD20×CD3 bispecific antibody in patients with CD20-positive B-cell malignancies (ELM-1): results from the relapsed or refractory non-Hodgkin lymphoma cohort in a single-arm, multicentre, phase 1 trial. Lancet Haematol..

[137] Patel K (2022). A phase 1 study of plamotamab, an anti-CD20 x anti-CD3 bispecific antibody, in patients with relapsed/refractory non-Hodgkin’s lymphoma: recommended dose safety/efficacy update and escalation exposure-response analysis. Blood.

[138] Patel K (2022). Phase 2 randomized, open-label, multicenter study to evaluate the efficacy and safety of plamotamab combined with tafasitamab (Tafa) plus lenalidomide (Len) Vs Tafa plus Len in relapsed or refractory DLBCL. Blood.

[139] Yeung YA (2020). An optimized full-length FLT3/CD3 bispecific antibody demonstrates potent anti-leukemia activity and reversible hematological toxicity. Mol. Ther..

[140] Reusch U (2016). Characterization of CD33/CD3 tetravalent bispecific tandem diabodies (TandAbs) for the treatment of acute myeloid leukemia. Clin. Cancer Res..

[141] Campagne O (2018). Integrated pharmacokinetic/pharmacodynamic model of a bispecific CD3xCD123 DART molecule in nonhuman primates: evaluation of activity and impact of immunogenicity. Clin. Cancer Res..

[142] Uy GL (2021). Flotetuzumab as salvage immunotherapy for refractory acute myeloid leukemia. Blood.

[143] Aigner M (2013). T lymphocytes can be effectively recruited for ex vivo and in vivo lysis of AML blasts by a novel CD33/CD3-bispecific BiTE antibody construct. Leukemia.

[144] Cheng P (2022). Immunodepletion of MDSC by AMV564, a novel bivalent, bispecific CD33/CD3 T cell engager, ex vivo in MDS and melanoma. Mol. Ther..

[145] Boyiadzis M (2023). First-in-human study of JNJ-63709178, a CD123/CD3 targeting antibody, in relapsed/refractory acute myeloid leukemia. Clin. Transl. Sci..

[146] Rettig, M. P. et al. Preliminary translational results from an ongoing phase 1 study of flotetuzumab, a CD123 x CD3 dart (R), in AML/MDS: rationale for combining flotetuzumab and anti-PD-1/PD-L1 immunotherapies. *Blood*. **130**, 637–637 (2017).

[147] Ravandi F (2018). Complete responses in relapsed/refractory acute myeloid leukemia (AML) patients on a weekly dosing schedule of XmAb14045, a CD123 x CD3 T cell-engaging bispecific antibody: initial results of a phase 1 study. Blood.

[148] van Loo PF (2019). MCLA-117, a CLEC12AxCD3 bispecific antibody targeting a leukaemic stem cell antigen, induces T cell-mediated AML blast lysis. Expert Opin. Biol. Ther..

[149] Dao T (2015). Therapeutic bispecific T-cell engager antibody targeting the intracellular oncoprotein WT1. Nat. Biotechnol..

[150] Krishnan AY (2022). MajesTEC-7: a phase 3, randomized study of teclistamab plus daratumumab plus lenalidomide (Tec-DR) versus daratumumab plus lenalidomide plus dexamethasone (DRd) in patients with newly diagnosed multiple myeloma who are either ineligible or not intended for autologous stem cell transplant. Blood.

[151] Moreau P (2022). Teclistamab in relapsed or refractory multiple myeloma. N. Engl. J. Med..

[152] Searle E (2022). Teclistamab in combination with subcutaneous daratumumab and lenalidomide in patients with multiple myeloma: results from one cohort of MajesTEC-2, a phase1b, multicohort study. Blood.

[153] Usmani SZ (2021). Teclistamab, a B-cell maturation antigenxCD3 bispecific antibody, in patients with relapsed or refractory multiple myeloma (MajesTEC-1): a multicentre, open-label, single-arm, phase 1 study. Lancet.

[154] Zamagni E (2022). MajesTEC-4 (EMN30): a phase 3 trial of teclistamab plus lenalidomide versus lenalidomide alone as maintenance therapy following autologous stem cell transplantation in patients with newly diagnosed multiple myeloma. Blood.

[155] Zonder JA (2022). Early, deep, and durable responses, and low rates of cytokine release syndrome with REGN5458, a BCMAxCD3 bispecific antibody, in a phase 1/2 first-in-human study in patients with relapsed/refractory multiple myeloma. Clin. Lymphoma Myeloma Leuk..

[156] Ferreri CJ (2022). Trial in progress: a phase II window of opportunity study of the BCMAxCD3 bispecific antibody REGN5458 in previously untreated patients with symptomatic multiple myeloma. Blood.

[157] Fonseca R (2022). MagnetisMM-9: an open-label, multicenter, non-randomized phase 1/2 study of elranatamab in patients with relapsed/refractory multiple myeloma. J. Clin. Oncol..

[158] Landgren O (2022). Magnetismm-4: an open label, phase 1b/2 umbrella study of elranatamab in combination with other anti-cancer treatments for patients with multiple myeloma. Blood.

[159] Wong SW (2022). Alnuctamab (ALNUC; BMS-986349; CC-93269), a B-cell maturation antigen (BCMA) x CD3 T-cell engager (TCE), in patients (pts) with relapsed/refractory multiple myeloma (RRMM): results from a phase 1 first-in-human clinical study. Blood.

[160] Fayon M (2021). Bi38-3 is a novel CD38/CD3 bispecific T-cell engager with low toxicity for the treatment of multiple myeloma. Haematologica.

[161] Zuch de Zafra CL (2019). Targeting multiple myeloma with AMG 424, a novel anti-CD38/CD3 bispecific T-cell-recruiting antibody optimized for cytotoxicity and cytokine release. Clin. Cancer Res.

[162] Topp MS (2020). Anti-B-cell maturation antigen BiTE molecule AMG 420 induces responses in multiple myeloma. J. Clin. Oncol..

[163] Kumar S (2021). A phase 1 first-in-human study of Tnb-383B, a BCMA x CD3 bispecific T-cell redirecting antibody, in patients with relapsed/refractory multiple myeloma. Blood.

[164] Harrison SJ (2020). A phase 1 first in human (FIH) study of AMG 701, an anti-B-cell maturation antigen (BCMA) half-life extended (HLE) BiTE (R) (bispecific T-cell engager) molecule, in relapsed/refractory (RR) multiple myeloma (MM). Blood.

[165] Lesokhin AM (2020). Preliminary safety, efficacy, pharmacokinetics, and pharmacodynamics of subcutaneously (SC) administered PF-06863135, a B-cell maturation antigen (BCMA)-CD3 bispecific antibody, in patients with relapsed/refractory multiple myeloma (RRMM). Blood.

[166] Mohan SR (2022). Initial results of dose escalation of ISB 1342, a novel CD3xCD38 bispecific antibody, in patients with relapsed/refractory multiple myeloma (RRMM). Blood.

[167] Richter JR (2018). Phase 1, multicenter, open-label study of single-agent bispecific antibody t-cell engager GBR 1342 in relapsed/refractory multiple myeloma. J. Clin. Oncol..

[168] Chari A (2022). Talquetamab, a T-cell-redirecting GPRC5D bispecific antibody for multiple myeloma. N. Engl. J. Med..

[169] Vij R (2022). CAMMA 1: a multicenter phase Ib trial evaluating the safety, pharmacokinetics, and activity of cevostamab-containing regimens in patients with relapsed or refractory multiple myeloma. J. Clin. Oncol..

[170] Zhao Y (2021). Tumor-intrinsic and -extrinsic determinants of response to blinatumomab in adults with B-ALL. Blood.

[171] Jabbour E (2018). Outcome of patients with relapsed/refractory acute lymphoblastic leukemia after blinatumomab failure: No change in the level of CD19 expression. Am. J. Hematol..

[172] Goebeler ME, Bargou RC (2020). T cell-engaging therapies - BiTEs and beyond. Nat. Rev. Clin. Oncol..

[173] Braig F (2017). Resistance to anti-CD19/CD3 BiTE in acute lymphoblastic leukemia may be mediated by disrupted CD19 membrane trafficking. Blood.

[174] Aldoss I (2017). Correlates of resistance and relapse during blinatumomab therapy for relapsed/refractory acute lymphoblastic leukemia. Am. J. Hematol..

[175] Ross T (2018). Preclinical characterization of AFM26, a novel B cell maturation antigen (BCMA)-directed tetravalent bispecific antibody for high affinity retargeting of NK cells against myeloma. Blood.

[176] Reusch U (2014). A novel tetravalent bispecific TandAb (CD30/CD16A) efficiently recruits NK cells for the lysis of CD30+ tumor cells. MAbs.

[177] Zhao L (2022). A novel CD19/CD22/CD3 trispecific antibody enhances therapeutic efficacy and overcomes immune escape against B-ALL. Blood.

[178] Chan WK (2018). A CS1-NKG2D bispecific antibody collectively activates cytolytic immune cells against multiple myeloma. Cancer Immunol. Res..

[179] Felices M (2016). CD16-IL15-CD33 trispecific killer engager (TriKE) overcomes cancer-induced immune suppression and induces natural killer cell-mediated control of MDS and AML via enhanced killing kinetics. Blood.

[180] Kantarjian HM (2016). Inotuzumab ozogamicin versus standard therapy for acute lymphoblastic leukemia. N. Engl. J. Med..

[181] Ansell SM (2022). Overall survival with brentuximab vedotin in stage III or IV Hodgkin’s lymphoma. N. Engl. J. Med..

[182] Chari RV, Miller ML, Widdison WC (2014). Antibody-drug conjugates: an emerging concept in cancer therapy. Angew. Chem. Int. Ed. Engl..

[183] Jin Y (2022). Stepping forward in antibody-drug conjugate development. Pharmacol. Ther..

[184] Meyer S, Rees AR (2015). The antibody molecule: from antitoxins to therapeutic antibodies. Soc. Hist. Med..

[185] Thomas A, Teicher BA, Hassan R (2016). Antibody-drug conjugates for cancer therapy. Lancet Oncol..

[186] Bargh JD, Isidro-Llobet A, Parker JS, Spring DR (2019). Cleavable linkers in antibody-drug conjugates. Chem. Soc. Rev..

[187] Advani A (2010). Safety, pharmacokinetics, and preliminary clinical activity of inotuzumab ozogamicin, a novel immunoconjugate for the treatment of B-cell non-Hodgkin’s lymphoma: results of a phase I study. J. Clin. Oncol..

[188] Brivio E (2021). A phase 1 study of inotuzumab ozogamicin in pediatric relapsed/refractory acute lymphoblastic leukemia (ITCC-059 study). Blood.

[189] Kreitman RJ (2012). Phase I trial of anti-CD22 recombinant immunotoxin moxetumomab pasudotox (CAT-8015 or HA22) in patients with hairy cell leukemia. J. Clin. Oncol..

[190] Short NJ (2018). A phase I study of moxetumomab pasudotox in adults with relapsed or refractory B-cell acute lymphoblastic leukaemia. Br. J. Haematol..

[191] Morschhauser F (2019). Polatuzumab vedotin or pinatuzumab vedotin plus rituximab in patients with relapsed or refractory non-Hodgkin lymphoma: final results from a phase 2 randomised study (ROMULUS). Lancet Haematol..

[192] Wayne AS (2010). Anti-CD22 immunotoxin RFB4(dsFv)-PE38 (BL22) for CD22-positive hematologic malignancies of childhood: preclinical studies and phase I clinical trial. Clin. Cancer Res..

[193] Kreitman RJ (2007). Phase II trial of CAT-3888 (BL22) in chemo-resistant hairy cell leukemia. J. Clin. Oncol..

[194] Kuruvilla J (2021). Pembrolizumab versus brentuximab vedotin in relapsed or refractory classical Hodgkin lymphoma (KEYNOTE-204): an interim analysis of a multicentre, randomised, open-label, phase 3 study. Lancet Oncol..

[195] Horwitz S (2022). The ECHELON-2 Trial: 5-year results of a randomized, phase III study of brentuximab vedotin with chemotherapy for CD30-positive peripheral T-cell lymphoma. Ann. Oncol..

[196] Pro B (2012). Brentuximab vedotin (SGN-35) in patients with relapsed or refractory systemic anaplastic large-cell lymphoma: results of a phase II study. J. Clin. Oncol..

[197] Prince HM (2017). Brentuximab vedotin or physician’s choice in CD30-positive cutaneous T-cell lymphoma (ALCANZA): an international, open-label, randomised, phase 3, multicentre trial. Lancet.

[198] Pollard JA (2021). Gemtuzumab ozogamicin improves event-free survival and reduces relapse in pediatric KMT2A-rearranged AML: results from the phase III children’s oncology group trial AAML0531. J. Clin. Oncol..

[199] Stein EM (2018). A phase 1 trial of vadastuximab talirine as monotherapy in patients with CD33-positive acute myeloid leukemia. Blood.

[200] Fathi AT (2018). A phase 1 trial of vadastuximab talirine combined with hypomethylating agents in patients with CD33-positive AML. Blood.

[201] Kovtun Y (2018). IMGN779, a novel CD33-targeting antibody-drug conjugate with DNA-alkylating activity, exhibits potent antitumor activity in models of AML. Mol. Cancer Ther..

[202] Cortes JE (2018). Maturing clinical profile of IMGN779, a next-generation CD33-targeting antibody-drug conjugate, in patients with relapsed or refractory acute myeloid leukemia. Blood.

[203] Caimi PF (2021). Loncastuximab tesirine in relapsed or refractory diffuse large B-cell lymphoma (LOTIS-2): a multicentre, open-label, single-arm, phase 2 trial. Lancet Oncol..

[204] Hamadani M (2021). Final results of a phase 1 study of loncastuximab tesirine in relapsed/refractory B-cell non-Hodgkin lymphoma. Blood.

[205] Carol H (2013). The anti-CD19 antibody-drug conjugate SAR3419 prevents hematolymphoid relapse postinduction therapy in preclinical models of pediatric acute lymphoblastic leukemia. Clin. Cancer Res..

[206] Ribrag V (2014). A dose-escalation study of SAR3419, an anti-CD19 antibody maytansinoid conjugate, administered by intravenous infusion once weekly in patients with relapsed/refractory B-cell non-Hodgkin lymphoma. Clin. Cancer Res..

[207] Jones L (2019). Preclinical activity of the antibody-drug conjugate denintuzumab mafodotin (SGN-CD19A) against pediatric acute lymphoblastic leukemia xenografts. Pediatr. Blood Cancer.

[208] Schindler J (2011). A phase I study of a combination of anti-CD19 and anti-CD22 immunotoxins (Combotox) in adult patients with refractory B-lineage acute lymphoblastic leukaemia. Br. J. Haematol..

[209] Diefenbach C (2021). Polatuzumab vedotin plus obinutuzumab and lenalidomide in patients with relapsed or refractory follicular lymphoma: a cohort of a multicentre, single-arm, phase 1b/2 study. Lancet Haematol..

[210] Sehn LH (2020). Polatuzumab vedotin in relapsed or refractory diffuse large B-cell lymphoma. J. Clin. Oncol..

[211] Lonial S (2020). Belantamab mafodotin for relapsed or refractory multiple myeloma (DREAMM-2): a two-arm, randomised, open-label, phase 2 study. Lancet Oncol..

[212] Figueroa-Vazquez V (2021). HDP-101, an anti-BCMA antibody-drug conjugate, safely delivers amanitin to induce cell death in proliferating and resting multiple myeloma cells. Mol. Cancer Ther..

[213] Stathis A (2018). Safety, tolerability, and preliminary activity of IMGN529, a CD37-targeted antibody-drug conjugate, in patients with relapsed or refractory B-cell non-Hodgkin lymphoma: a dose-escalation, phase I study. Invest. New Drugs.

[214] Pereira DS (2015). AGS67E, an anti-CD37 monomethyl auristatin E antibody-drug conjugate as a potential therapeutic for B/T-cell malignancies and AML: a new role for CD37 in AML. Mol. Cancer Ther..

[215] Pereira DS (2014). Ags67e, an anti-cd37 monomethyl auristatin e antibody (mmae) drug conjugate as a potential therapeutic for non-hodgkin’s lymphoma, chronic lymphocytic leukemia and acute myeloid leukemia. Cancer Res..

[216] Kelly KR (2021). Indatuximab ravtansine plus dexamethasone with lenalidomide or pomalidomide in relapsed or refractory multiple myeloma: a multicentre, phase 1/2a study. Lancet Haematol..

[217] Ailawadhi S (2019). A phase I study to assess the safety and pharmacokinetics of single-agent lorvotuzumab mertansine (IMGN901) in patients with relapsed and/or refractory CD-56-positive multiple myeloma. Clin. Lymphoma Myeloma Leuk..

[218] Govindan SV (2013). Milatuzumab-SN-38 conjugates for the treatment of CD74+ cancers. Mol. Cancer Ther..

[219] Huang WT (2022). Preclinical activity of LM-305 targeting G-protein-coupled receptor class 5 member D (GPRC5D) antibody drug conjugate for the treatment of multiple myeloma. Cancer Res..

[220] Daver N (2022). Broad activity for the pivekimab sunirine (PVEK, IMGN632), azacitidine, and venetoclax triplet in high-risk patients with relapsed/refractory acute myeloid leukemia (AML). Blood.

[221] Hamadani M (2021). Camidanlumab tesirine in patients with relapsed or refractory lymphoma: a phase 1, open-label, multicentre, dose-escalation, dose-expansion study. Lancet Haematol..

[222] Younes A (2012). Results of a pivotal phase II study of brentuximab vedotin for patients with relapsed or refractory Hodgkin’s lymphoma. J. Clin. Oncol..

[223] Lamb YN (2017). Inotuzumab ozogamicin: firstg global approval. Drugs.

[224] Mori J, Tsuda K, Tanimoto T (2016). Inotuzumab ozogamicin for acute lymphoblastic leukemia. N. Engl. J. Med..

[225] Leong S, Lam HPJ, Kirkham Z, Popat R (2023). Antibody drug conjugates for the treatment of multiple myeloma. Am. J. Hematol..

[226] Bartok O (2021). Anti-tumour immunity induces aberrant peptide presentation in melanoma. Nature.

[227] Röhrig UF (2019). Inhibition mechanisms of indoleamine 2,3-dioxygenase 1 (IDO1). J. Med. Chem..

[228] Ahmadzadeh M (2009). Tumor antigen-specific CD8 T cells infiltrating the tumor express high levels of PD-1 and are functionally impaired. Blood.

[229] Wei SC, Duffy CR, Allison JP (2018). Fundamental mechanisms of immune checkpoint blockade therapy. Cancer Discov..

[230] Qin G (2020). NPM1 upregulates the transcription of PD-L1 and suppresses T cell activity in triple-negative breast cancer. Nat. Commun..

[231] Fang W (2021). Progranulin induces immune escape in breast cancer via up-regulating PD-L1 expression on tumor-associated macrophages (TAMs) and promoting CD8(+) T cell exclusion. J. Exp. Clin. Cancer Res..

[232] Gordon SR (2017). PD-1 expression by tumour-associated macrophages inhibits phagocytosis and tumour immunity. Nature.

[233] Juneja VR (2017). PD-L1 on tumor cells is sufficient for immune evasion in immunogenic tumors and inhibits CD8 T cell cytotoxicity. J. Exp. Med..

[234] Patsoukis N, Wang Q, Strauss L, Boussiotis VA (2020). Revisiting the PD-1 pathway. Sci. Adv..

[235] Mahoney KM (2022). Soluble PD-L1 as an early marker of progressive disease on nivolumab. J. Immunother. Cancer.

[236] Tekguc M (2021). Treg-expressed CTLA-4 depletes CD80/CD86 by trogocytosis, releasing free PD-L1 on antigen-presenting cells. Proc. Natl Acad. Sci. USA.

[237] Wei SC (2017). Distinct cellular mechanisms underlie anti-CTLA-4 and anti-PD-1 checkpoint blockade. Cell.

[238] Garris CS (2018). Successful anti-PD-1 cancer immunotherapy requires T cell-dendritic cell crosstalk involving the cytokines IFN-γ and IL-12. Immunity.

[239] Mayoux M (2020). Dendritic cells dictate responses to PD-L1 blockade cancer immunotherapy. Sci. Transl. Med..

[240] Budimir N, Thomas GD, Dolina JS, Salek-Ardakani S (2022). Reversing T-cell exhaustion in cancer: lessons learned from PD-1/PD-L1 immune checkpoint blockade. Cancer Immunol. Res..

[241] Walker LS, Sansom DM (2011). The emerging role of CTLA4 as a cell-extrinsic regulator of T cell responses. Nat. Rev. Immunol..

[242] Wing K, Yamaguchi T, Sakaguchi S (2011). Cell-autonomous and -non-autonomous roles of CTLA-4 in immune regulation. Trends Immunol..

[243] Mahoney KM, Rennert PD, Freeman GJ (2015). Combination cancer immunotherapy and new immunomodulatory targets. Nat. Rev. Drug Discov..

[244] Mayes PA, Hance KW, Hoos A (2018). The promise and challenges of immune agonist antibody development in cancer. Nat. Rev. Drug Discov..

[245] Romano E (2015). Ipilimumab-dependent cell-mediated cytotoxicity of regulatory T cells ex vivo by nonclassical monocytes in melanoma patients. Proc. Natl Acad. Sci. USA.

[246] Vesely MD, Zhang T, Chen L (2022). Resistance mechanisms to anti-PD cancer immunotherapy. Annu. Rev. Immunol..

[247] Chen L (2018). CD38-mediated immunosuppression as a mechanism of tumor cell escape from PD-1/PD-L1 blockade. Cancer Discov..

[248] Banta KL (2022). Mechanistic convergence of the TIGIT and PD-1 inhibitory pathways necessitates co-blockade to optimize anti-tumor CD8(+) T cell responses. Immunity.

[249] Strauss L (2020). Targeted deletion of PD-1 in myeloid cells induces antitumor immunity. Sci. Immunol..

[250] Verma V (2019). PD-1 blockade in subprimed CD8 cells induces dysfunctional PD-1(+)CD38(hi) cells and anti-PD-1 resistance. Nat. Immunol..

[251] Hashimoto M (2022). PD-1 combination therapy with IL-2 modifies CD8(+) T cell exhaustion program. Nature.

[252] Vari F (2018). Immune evasion via PD-1/PD-L1 on NK cells and monocyte/macrophages is more prominent in Hodgkin lymphoma than DLBCL. Blood.

[253] Yi M (2022). Combination strategies with PD-1/PD-L1 blockade: current advances and future directions. Mol. Cancer.

[254] Zhang L (2020). Immunotherapy for advanced hepatocellular carcinoma, where are we?. Biochim. Biophys. Acta Rev. Cancer.

[255] Sommaggio R (2020). Adoptive cell therapy of triple negative breast cancer with redirected cytokine-induced killer cells. Oncoimmunology.

[256] Krishna S (2020). Stem-like CD8 T cells mediate response of adoptive cell immunotherapy against human cancer. Science.

[257] Lopez RD (2000). CD2-mediated IL-12-dependent signals render human gamma delta-T cells resistant to mitogen-induced apoptosis, permitting the large-scale ex vivo expansion of functionally distinct lymphocytes: implications for the development of adoptive immunotherapy strategies. Blood.

[258] Laskowski TJ, Biederstädt A, Rezvani K (2022). Natural killer cells in antitumour adoptive cell immunotherapy. Nat. Rev. Cancer.

[259] Ping Y, Liu C, Zhang Y (2018). T-cell receptor-engineered T cells for cancer treatment: current status and future directions. Protein Cell.

[260] Zhang X (2022). CAR-T cell therapy in hematological malignancies: current opportunities and challenges. Front. Immunol..

[261] Tanaka J (2021). Recent advances in chimeric antigen receptor natural killer cell therapy for overcoming intractable hematological malignancies. Hematol. Oncol..

[262] Yilmaz A, Cui H, Caligiuri MA, Yu J (2020). Chimeric antigen receptor-engineered natural killer cells for cancer immunotherapy. J. Hematol. Oncol..

[263] Gross G, Waks T, Eshhar Z (1989). Expression of immunoglobulin-T-cell receptor chimeric molecules as functional receptors with antibody-type specificity. Proc. Natl Acad. Sci. USA.

[264] Heuser C (2003). T-cell activation by recombinant immunoreceptors: impact of the intracellular signalling domain on the stability of receptor expression and antigen-specific activation of grafted T cells. Gene Ther..

[265] Jensen MC (2010). Antitransgene rejection responses contribute to attenuated persistence of adoptively transferred CD20/CD19-specific chimeric antigen receptor redirected T cells in humans. Biol. Blood Marrow Transplant..

[266] Locke FL (2017). Phase 1 results of ZUMA-1: a multicenter study of KTE-C19 anti-CD19 CAR T cell therapy in refractory aggressive lymphoma. Mol. Ther..

[267] Abramson JS (2020). Lisocabtagene maraleucel for patients with relapsed or refractory large B-cell lymphomas (TRANSCEND NHL 001): a multicentre seamless design study. Lancet.

[268] van der Stegen SJ, Hamieh M, Sadelain M (2015). The pharmacology of second-generation chimeric antigen receptors. Nat. Rev. Drug Discov..

[269] Bôle-Richard E (2020). CD28/4-1BB CD123 CAR T cells in blastic plasmacytoid dendritic cell neoplasm. Leukemia.

[270] Wang J (2018). CAR-T cells targeting CLL-1 as an approach to treat acute myeloid leukemia. J. Hematol. Oncol..

[271] Roselli E (2021). 4-1BB and optimized CD28 co-stimulation enhances function of human mono-specific and bi-specific third-generation CAR T cells. J. Immunother. Cancer.

[272] Liu Z (2023). Safety and antitumor activity of GD2-Specific 4SCAR-T cells in patients with glioblastoma. Mol. Cancer.

[273] Zhou X (2020). Phase I trial of fourth-generation anti-CD19 chimeric antigen receptor T cells against relapsed or refractory B cell non-Hodgkin lymphomas. Front. Immunol..

[274] Kagoya Y (2018). A novel chimeric antigen receptor containing a JAK-STAT signaling domain mediates superior antitumor effects. Nat. Med..

[275] Turtle CJ (2016). CD19 CAR-T cells of defined CD4+:CD8+ composition in adult B cell ALL patients. J. Clin. Invest..

[276] Maus MV (2013). T cells expressing chimeric antigen receptors can cause anaphylaxis in humans. Cancer Immunol. Res..

[277] Gu R (2020). Efficacy and safety of CD19 CAR T constructed with a new anti-CD19 chimeric antigen receptor in relapsed or refractory acute lymphoblastic leukemia. J. Hematol. Oncol..

[278] Wang D (2021). A phase 1 study of a novel fully human BCMA-targeting CAR (CT103A) in patients with relapsed/refractory multiple myeloma. Blood.

[279] Song F (2023). Safety and efficacy of autologous and allogeneic humanized CD19-targeted CAR-T cell therapy for patients with relapsed/refractory B-ALL. J. Immunother. Cancer.

[280] Depil S (2020). ‘Off-the-shelf’ allogeneic CAR T cells: development and challenges. Nat. Rev. Drug Discov..

[281] Hu Y (2022). Genetically modified CD7-targeting allogeneic CAR-T cell therapy with enhanced efficacy for relapsed/refractory CD7-positive hematological malignancies: a phase I clinical study. Cell Res..

[282] Parikh, R. H., Lonial, S. Chimeric antigen receptor T-cell therapy in multiple myeloma: a comprehensive review of current data and implications for clinical practice. *CA: A Cancer J. Clin.* **73**, 275–285 (2023). 10.3322/caac.21771 36627265

[283] Hay KA, Turtle CJ (2017). Chimeric antigen receptor (CAR) T Cells: lessons learned from targeting of CD19 in B-cell malignancies. Drugs.

[284] Pan J (2021). Donor-derived CD7 chimeric antigen receptor T cells for T-cell acute lymphoblastic leukemia: first-in-human, phase I trial. J. Clin. Oncol..

[285] Zhang M (2022). Autologous nanobody-derived fratricide-resistant CD7-CAR T-cell therapy for patients with relapsed and refractory T-cell acute lymphoblastic leukemia/lymphoma. Clin. Cancer Res..

[286] Freiwan A (2022). Engineering naturally occurring CD7- T cells for the immunotherapy of hematological malignancies. Blood.

[287] Gruss HJ (1994). Pleiotropic effects of the CD30 ligand on CD30-expressing cells and lymphoma cell lines. Blood.

[288] Walter RB, Appelbaum FR, Estey EH, Bernstein ID (2012). Acute myeloid leukemia stem cells and CD33-targeted immunotherapy. Blood.

[289] Cui Q (2021). CD38-directed CAR-T cell therapy: a novel immunotherapy strategy for relapsed acute myeloid leukemia after allogeneic hematopoietic stem cell transplantation. J. Hematol. Oncol..

[290] Zhang M (2023). GPRC5D CAR T cells (OriCAR-017) in patients with relapsed or refractory multiple myeloma (POLARIS): a first-in-human, single-centre, single-arm, phase 1 trial. Lancet Haematol..

[291] Mei H (2021). A bispecific CAR-T cell therapy targeting BCMA and CD38 in relapsed or refractory multiple myeloma. J. Hematol. Oncol..

[292] Zhang Z (2020). Point mutation in CD19 facilitates immune escape of B cell lymphoma from CAR-T cell therapy. J. Immunother. Cancer.

[293] Samur MK (2021). Biallelic loss of BCMA as a resistance mechanism to CAR T cell therapy in a patient with multiple myeloma. Nat. Commun..

[294] Ruella M (2018). Induction of resistance to chimeric antigen receptor T cell therapy by transduction of a single leukemic B cell. Nat. Med..

[295] Hamieh M (2019). CAR T cell trogocytosis and cooperative killing regulate tumour antigen escape. Nature.

[296] Nian Z (2021). Rapamycin pretreatment rescues the bone marrow AML cell elimination capacity of CAR-T cells. Clin. Cancer Res..

[297] Jain MD (2021). Tumor interferon signaling and suppressive myeloid cells are associated with CAR T-cell failure in large B-cell lymphoma. Blood.

[298] Shen Y (2023). Serum soluble BCMA can be used to monitor relapse of multiple myeloma patients after chimeric antigen receptor T-cell immunotherapy. Curr. Res. Transl. Med..

[299] Mailankody S (2022). GPRC5D-targeted CAR T cells for myeloma. N. Engl. J. Med..

[300] Smith EL (2019). GPRC5D is a target for the immunotherapy of multiple myeloma with rationally designed CAR T cells. Sci. Transl. Med..

[301] Hu Y (2021). CRISPR/Cas9-engineered universal CD19/CD22 dual-targeted CAR-T cell therapy for relapsed/refractory B-cell acute lymphoblastic leukemia. Clin. Cancer Res..

[302] Tong C (2020). Optimized tandem CD19/CD20 CAR-engineered T cells in refractory/relapsed B-cell lymphoma. Blood.

[303] He X (2020). Bispecific and split CAR T cells targeting CD13 and TIM3 eradicate acute myeloid leukemia. Blood.

[304] Li KX (2022). A novel approach for relapsed/refractory FLT3(mut+) acute myeloid leukaemia: synergistic effect of the combination of bispecific FLT3scFv/NKG2D-CAR T cells and gilteritinib. Mol. Cancer.

[305] Fousek K (2021). CAR T-cells that target acute B-lineage leukemia irrespective of CD19 expression. Leukemia.

[306] Han X, Wang Y, Wei J, Han W (2019). Multi-antigen-targeted chimeric antigen receptor T cells for cancer therapy. J. Hematol. Oncol..

[307] Rabilloud T (2021). Single-cell profiling identifies pre-existing CD19-negative subclones in a B-ALL patient with CD19-negative relapse after CAR-T therapy. Nat. Commun..

[308] Shao L (2022). Genome-wide profiling of retroviral DNA integration and its effect on clinical pre-infusion CAR T-cell products. J. Transl. Med..

[309] Xie G (2020). CAR-NK cells: a promising cellular immunotherapy for cancer. EBioMedicine.

[310] Pan K (2022). CAR race to cancer immunotherapy: from CAR T, CAR NK to CAR macrophage therapy. J. Exp. Clin. Cancer Res..

[311] Pao SC, Chu MT, Hung SI (2022). Therapeutic vaccines targeting neoantigens to induce T-cell immunity against cancers. Pharmaceutics.

[312] Keilholz U (2009). A clinical and immunologic phase 2 trial of Wilms tumor gene product 1 (WT1) peptide vaccination in patients with AML and MDS. Blood.

[313] Van Tendeloo VF (2010). Induction of complete and molecular remissions in acute myeloid leukemia by Wilms’ tumor 1 antigen-targeted dendritic cell vaccination. Proc. Natl Acad. Sci. USA.

[314] Saxena M, van der Burg SH, Melief CJM, Bhardwaj N (2021). Therapeutic cancer vaccines. Nat. Rev. Cancer.

[315] Smith C (2009). Discerning regulation of cis- and trans-presentation of CD8+ T-cell epitopes by EBV-encoded oncogene LMP-1 through self-aggregation. Blood.

[316] Frank MJ (2020). Autologous tumor cell vaccine induces antitumor T cell immune responses in patients with mantle cell lymphoma: A phase I/II trial. J. Exp. Med..

[317] Neelapu SS (2005). Vaccine-induced tumor-specific immunity despite severe B-cell depletion in mantle cell lymphoma. Nat. Med..

[318] Maslak PG (2018). Phase 2 trial of a multivalent WT1 peptide vaccine (galinpepimut-S) in acute myeloid leukemia. Blood Adv..

[319] Hu Z, Ott PA, Wu CJ (2018). Towards personalized, tumour-specific, therapeutic vaccines for cancer. Nat. Rev. Immunol..

[320] Tay, B. Q. et al. Evolution of cancer vaccines-challenges, achievements, and future directions. *Vaccines (Basel)*. **9** (2021). 10.3390/vaccines9050535 PMC8160852 34065557

[321] French RR, Chan HT, Tutt AL, Glennie MJ (1999). CD40 antibody evokes a cytotoxic T-cell response that eradicates lymphoma and bypasses T-cell help. Nat. Med..

[322] Sotomayor EM (1999). Conversion of tumor-specific CD4+ T-cell tolerance to T-cell priming through in vivo ligation of CD40. Nat. Med..

[323] Nagorsen D, Baeuerle PA (2011). Immunomodulatory therapy of cancer with T cell-engaging BiTE antibody blinatumomab. Exp. Cell Res..

[324] Lu J, Jiang G (2022). The journey of CAR-T therapy in hematological malignancies. Mol. Cancer.

[325] Pardoll DM (2012). The blockade of immune checkpoints in cancer immunotherapy. Nat. Rev. Cancer.

[326] Postow MA, Callahan MK, Wolchok JD (2015). Immune checkpoint blockade in cancer therapy. J. Clin. Oncol..

[327] Baker KS (1999). Autologous hematopoietic stem-cell transplantation for relapsed or refractory Hodgkin’s disease in children and adolescents. J. Clin. Oncol..

[328] Eckert C (2013). Use of allogeneic hematopoietic stem-cell transplantation based on minimal residual disease response improves outcomes for children with relapsed acute lymphoblastic leukemia in the intermediate-risk group. J. Clin. Oncol..

[329] Pession A (2013). Results of the AIEOP AML 2002/01 multicenter prospective trial for the treatment of children with acute myeloid leukemia. Blood.

[330] Vose JM (2002). Autologous transplantation for aggressive non-Hodgkin’s lymphoma: results of a randomized trial evaluating graft source and minimal residual disease. J. Clin. Oncol..

[331] Majolino I, Pearce R, Taghipour G, Goldstone AH (1997). Peripheral-blood stem-cell transplantation versus autologous bone marrow transplantation in Hodgkin’s and non-Hodgkin’s lymphomas: a new matched-pair analysis of the European Group for Blood and Marrow Transplantation Registry Data. Lymphoma Working Party of the European Group for Blood and Marrow Transplantation. J. Clin. Oncol..

[332] Bertz H, Illerhaus G, Veelken H, Finke J (2002). Allogeneic hematopoetic stem-cell transplantation for patients with relapsed or refractory lymphomas: comparison of high-dose conventional conditioning versus fludarabine-based reduced-intensity regimens. Ann. Oncol..

[333] Anderlini P (2008). Fludarabine-melphalan as a preparative regimen for reduced-intensity conditioning allogeneic stem cell transplantation in relapsed and refractory Hodgkin’s lymphoma: the updated M.D. Anderson Cancer Center experience. Haematologica.

[334] Sureda A (2012). Allogeneic stem cell transplantation after reduced intensity conditioning in patients with relapsed or refractory Hodgkin’s lymphoma. Results of the HDR-ALLO study - a prospective clinical trial by the Grupo Español de Linfomas/Trasplante de Médula Osea (GEL/TAMO) and the Lymphoma Working Party of the European Group for Blood and Marrow Transplantation. Haematologica.

[335] Beelen DW (2022). Treosulfan compared with reduced-intensity busulfan improves allogeneic hematopoietic cell transplantation outcomes of older acute myeloid leukemia and myelodysplastic syndrome patients: final analysis of a prospective randomized trial. Am. J. Hematol..

[336] Russell NH (2022). Outcomes of older patients aged 60 to 70 years undergoing reduced intensity transplant for acute myeloblastic leukemia: results of the NCRI acute myeloid leukemia 16 trial. Haematologica.

[337] Nakamura R (2021). Biologic assignment trial of reduced-intensity hematopoietic cell transplantation based on donor availability in patients 50–75 years of age with advanced myelodysplastic syndrome. J. Clin. Oncol..

[338] Wang Y (2015). Haploidentical vs identical-sibling transplant for AML in remission: a multicenter, prospective study. Blood.

[339] Wang Y (2016). Haploidentical versus matched-sibling transplant in adults with Philadelphia-negative high-risk acute lymphoblastic leukemia: a biologically phase III randomized study. Clin. Cancer Res..

[340] Lu Y (2020). Unmanipulated haplo-identical donor transplantation compared with identical sibling donor had better anti-leukemia effect for refractory/relapsed acute myeloid leukemia not in remission status. Ann. Hematol..

[341] Zheng FM (2020). Haploidentical- versus identical-sibling transplant for high-risk pediatric AML: a multi-center study. Cancer Commun..

[342] Zhou X (2014). Long-term outcome after haploidentical stem cell transplant and infusion of T cells expressing the inducible caspase 9 safety transgene. Blood.

[343] Martelli MF (2014). HLA-haploidentical transplantation with regulatory and conventional T-cell adoptive immunotherapy prevents acute leukemia relapse. Blood.

[344] McLaughlin P (1998). Rituximab chimeric anti-CD20 monoclonal antibody therapy for relapsed indolent lymphoma: half of patients respond to a four-dose treatment program. J. Clin. Oncol..

[345] Foran JM (2000). European phase II study of rituximab (chimeric anti-CD20 monoclonal antibody) for patients with newly diagnosed mantle-cell lymphoma and previously treated mantle-cell lymphoma, immunocytoma, and small B-cell lymphocytic lymphoma. J. Clin. Oncol..

[346] Lenz G (2005). Immunochemotherapy with rituximab and cyclophosphamide, doxorubicin, vincristine, and prednisone significantly improves response and time to treatment failure, but not long-term outcome in patients with previously untreated mantle cell lymphoma: results of a prospective randomized trial of the German Low Grade Lymphoma Study Group (GLSG). J. Clin. Oncol..

[347] Mounier N (2003). Rituximab plus CHOP (R-CHOP) overcomes bcl-2-associated resistance to chemotherapy in elderly patients with diffuse large B-cell lymphoma (DLBCL). Blood.

[348] Eichhorst B (2021). Chronic lymphocytic leukaemia: ESMO clinical practice guidelines for diagnosis, treatment and follow-up. Ann. Oncol..

[349] Hallek M (2010). Addition of rituximab to fludarabine and cyclophosphamide in patients with chronic lymphocytic leukaemia: a randomised, open-label, phase 3 trial. Lancet.

[350] Hagenbeek A (2008). First clinical use of ofatumumab, a novel fully human anti-CD20 monoclonal antibody in relapsed or refractory follicular lymphoma: results of a phase 1/2 trial. Blood.

[351] Cheson BD (2018). Overall survival benefit in patients with rituximab-refractory indolent non-Hodgkin lymphoma who received obinutuzumab plus bendamustine induction and obinutuzumab maintenance in the GADOLIN study. J. Clin. Oncol..

[352] Jurczak W (2018). Phase IIa study of the CD19 antibody MOR208 in patients with relapsed or refractory B-cell non-Hodgkin’s lymphoma. Ann. Oncol..

[353] Tilly H (2019). Polatuzumab vedotin in combination with immunochemotherapy in patients with previously untreated diffuse large B-cell lymphoma: an open-label, non-randomised, phase 1b-2 study. Lancet Oncol..

[354] Sharman JP (2021). Ublituximab plus ibrutinib versus ibrutinib alone for patients with relapsed or refractory high-risk chronic lymphocytic leukaemia (GENUINE): a phase 3, multicentre, open-label, randomised trial. Lancet Haematol..

[355] Bargou R (2008). Tumor regression in cancer patients by very low doses of a T cell-engaging antibody. Science.

[356] Topp MS (2011). Targeted therapy with the T-cell-engaging antibody blinatumomab of chemotherapy-refractory minimal residual disease in B-lineage acute lymphoblastic leukemia patients results in high response rate and prolonged leukemia-free survival. J. Clin. Oncol..

[357] Topp MS (2014). Phase II trial of the anti-CD19 bispecific T cell-engager blinatumomab shows hematologic and molecular remissions in patients with relapsed or refractory B-precursor acute lymphoblastic leukemia. J. Clin. Oncol..

[358] Klinger M (2012). Immunopharmacologic response of patients with B-lineage acute lymphoblastic leukemia to continuous infusion of T cell-engaging CD19/CD3-bispecific BiTE antibody blinatumomab. Blood.

[359] Brown PA (2021). Effect of postreinduction therapy consolidation with blinatumomab vs chemotherapy on disease-free survival in children, adolescents, and young adults with first relapse of B-cell acute lymphoblastic leukemia: a randomized clinical trial. JAMA.

[360] Locatelli F (2021). Effect of blinatumomab vs chemotherapy on event-free survival among children with high-risk first-relapse B-cell acute lymphoblastic leukemia: a randomized clinical trial. JAMA.

[361] Jabbour E (2022). Hyper-CVAD and sequential blinatumomab for newly diagnosed Philadelphia chromosome-negative B-cell acute lymphocytic leukaemia: a single-arm, single-centre, phase 2 trial. Lancet Haematol..

[362] Jabbour E (2023). Ponatinib and blinatumomab for Philadelphia chromosome-positive acute lymphoblastic leukaemia: a US, single-centre, single-arm, phase 2 trial. Lancet Haematol..

[363] Foà R (2020). Dasatinib-blinatumomab for Ph-positive acute lymphoblastic leukemia in adults. N. Engl. J. Med..

[364] Coyle L (2020). Open-label, phase 2 study of blinatumomab as second salvage therapy in adults with relapsed/refractory aggressive B-cell non-Hodgkin lymphoma. Leuk. Lymphoma.

[365] Katz DA (2022). Open-label, phase 2 study of blinatumomab after frontline R-chemotherapy in adults with newly diagnosed, high-risk DLBCL. Leuk. Lymphoma.

[366] Grosicki S (2022). Elranatamab in combination with daratumumab for patients (pts) with relapsed/refractory multiple myeloma (RRMM): results from the phase 3 magnetismm-5 study safety lead-in cohort. Blood.

[367] Lesokhin A (2021). Magnetismm-3: an open-label, multicenter, non-randomized phase 2 study of elranatamab (PF-06863135) in patients with relapsed or refractory multiple myeloma. Blood.

[368] Mateos MV (2022). MagnetisMM-7: an open label, randomized, phase 3 study of elranatamab versus lenalidomide in patients with newly diagnosed multiple myeloma who are minimal residual disease-positive after transplant. Clin. Lymphoma Myeloma Leuk..

[369] Sievers EL (2001). Efficacy and safety of gemtuzumab ozogamicin in patients with CD33-positive acute myeloid leukemia in first relapse. J. Clin. Oncol..

[370] Petersdorf SH (2013). A phase 3 study of gemtuzumab ozogamicin during induction and postconsolidation therapy in younger patients with acute myeloid leukemia. Blood.

[371] Löwenberg B (2010). Gemtuzumab ozogamicin as postremission treatment in AML at 60 years of age or more: results of a multicenter phase 3 study. Blood.

[372] Hasle H (2012). Gemtuzumab ozogamicin as postconsolidation therapy does not prevent relapse in children with AML: results from NOPHO-AML 2004. Blood.

[373] Amadori S (2013). Sequential combination of gemtuzumab ozogamicin and standard chemotherapy in older patients with newly diagnosed acute myeloid leukemia: results of a randomized phase III trial by the EORTC and GIMEMA consortium (AML-17). J. Clin. Oncol..

[374] Castaigne S (2012). Effect of gemtuzumab ozogamicin on survival of adult patients with de-novo acute myeloid leukaemia (ALFA-0701): a randomised, open-label, phase 3 study. Lancet.

[375] Burnett AK (2012). Addition of gemtuzumab ozogamicin to induction chemotherapy improves survival in older patients with acute myeloid leukemia. J. Clin. Oncol..

[376] Gamis AS (2014). Gemtuzumab ozogamicin in children and adolescents with de novo acute myeloid leukemia improves event-free survival by reducing relapse risk: results from the randomized phase III Children’s Oncology Group trial AAML0531. J. Clin. Oncol..

[377] Olombel G (2016). The level of blast CD33 expression positively impacts the effect of gemtuzumab ozogamicin in patients with acute myeloid leukemia. Blood.

[378] Fournier E (2020). Mutational profile and benefit of gemtuzumab ozogamicin in acute myeloid leukemia. Blood.

[379] Schlenk RF (2020). Gemtuzumab ozogamicin in NPM1-mutated acute myeloid leukemia: early results from the prospective randomized AMLSG 09-09 phase III study. J. Clin. Oncol..

[380] Borthakur G (2022). Retrospective comparison of survival and responses to fludarabine, cytarabine, GCSF (FLAG) in combination with gemtuzumab ozogamicin (GO) or idarubicin (IDA) in patients with newly diagnosed core binding factor (CBF) acute myelogenous leukemia: MD Anderson experience in 174 patients. Am. J. Hematol..

[381] Kantarjian H (2012). Inotuzumab ozogamicin, an anti-CD22-calecheamicin conjugate, for refractory and relapsed acute lymphocytic leukaemia: a phase 2 study. Lancet Oncol..

[382] Jabbour E (2018). Salvage chemoimmunotherapy with inotuzumab ozogamicin combined with mini-hyper-CVD for patients with relapsed or refractory Philadelphia chromosome-negative acute lymphoblastic leukemia: a phase 2 clinical trial. JAMA Oncol..

[383] Kantarjian H (2018). Inotuzumab ozogamicin in combination with low-intensity chemotherapy for older patients with Philadelphia chromosome-negative acute lymphoblastic leukaemia: a single-arm, phase 2 study. Lancet Oncol..

[384] Kreitman RJ (2021). Moxetumomab pasudotox in heavily pre-treated patients with relapsed/refractory hairy cell leukemia (HCL): long-term follow-up from the pivotal trial. J. Hematol. Oncol..

[385] Trudel S (2018). Targeting B-cell maturation antigen with GSK2857916 antibody-drug conjugate in relapsed or refractory multiple myeloma (BMA117159): a dose escalation and expansion phase 1 trial. Lancet Oncol..

[386] Quach H (2022). Safety and clinical activity of belantamab mafodotin with lenalidomide plus dexamethasone in patients with relapsed/refractory multiple myeloma (RRMM): DREAMM-6 arm-A interim analysis. Clin. Lymphoma Myeloma Leuk..

[387] Ansell SM (2015). PD-1 blockade with nivolumab in relapsed or refractory Hodgkin’s lymphoma. N. Engl. J. Med..

[388] Younes A (2016). Nivolumab for classical Hodgkin’s lymphoma after failure of both autologous stem-cell transplantation and brentuximab vedotin: a multicentre, multicohort, single-arm phase 2 trial. Lancet Oncol..

[389] Schnorfeil FM (2015). T cells are functionally not impaired in AML: increased PD-1 expression is only seen at time of relapse and correlates with a shift towards the memory T cell compartment. J. Hematol. Oncol..

[390] Armand P (2013). Disabling immune tolerance by programmed death-1 blockade with pidilizumab after autologous hematopoietic stem-cell transplantation for diffuse large B-cell lymphoma: results of an international phase II trial. J. Clin. Oncol..

[391] Armand P (2015). Immune checkpoint blockade in hematologic malignancies. Blood.

[392] Green MR (2010). Integrative analysis reveals selective 9p24.1 amplification, increased PD-1 ligand expression, and further induction via JAK2 in nodular sclerosing Hodgkin lymphoma and primary mediastinal large B-cell lymphoma. Blood.

[393] Roemer MG (2016). PD-L1 and PD-L2 genetic alterations define classical hodgkin lymphoma and predict outcome. J. Clin. Oncol..

[394] Green MR (2012). Constitutive AP-1 activity and EBV infection induce PD-L1 in Hodgkin lymphomas and posttransplant lymphoproliferative disorders: implications for targeted therapy. Clin. Cancer Res..

[395] Merryman RW, Armand P, Wright KT, Rodig SJ (2017). Checkpoint blockade in Hodgkin and non-Hodgkin lymphoma. Blood Adv..

[396] Bashey A (2009). CTLA4 blockade with ipilimumab to treat relapse of malignancy after allogeneic hematopoietic cell transplantation. Blood.

[397] Ansell SM (2009). Phase I study of ipilimumab, an anti-CTLA-4 monoclonal antibody, in patients with relapsed and refractory B-cell non-Hodgkin lymphoma. Clin. Cancer Res..

[398] Diefenbach CS (2020). Ipilimumab, nivolumab, and brentuximab vedotin combination therapies in patients with relapsed or refractory Hodgkin lymphoma: phase 1 results of an open-label, multicentre, phase 1/2 trial. Lancet Haematol..

[399] Armand P (2021). A phase 1b study of dual PD-1 and CTLA-4 or KIR blockade in patients with relapsed/refractory lymphoid malignancies. Leukemia.

[400] Mei MG (2022). Response-adapted anti-PD-1-based salvage therapy for Hodgkin lymphoma with nivolumab alone or in combination with ICE. Blood.

[401] Deng Q (2020). Characteristics of anti-CD19 CAR T cell infusion products associated with efficacy and toxicity in patients with large B cell lymphomas. Nat. Med..

[402] Nayak L (2017). PD-1 blockade with nivolumab in relapsed/refractory primary central nervous system and testicular lymphoma. Blood.

[403] Zinzani PL (2019). Nivolumab combined with brentuximab vedotin for relapsed/refractory primary mediastinal large B-cell lymphoma: efficacy and safety from the phase II checkMate 436 study. J. Clin. Oncol..

[404] Ansell SM (2019). Nivolumab for relapsed/refractory diffuse large B-cell lymphoma in patients ineligible for or having failed autologous transplantation: a single-arm, phase II study. J. Clin. Oncol..

[405] Chen R (2017). Phase II study of the efficacy and safety of pembrolizumab for relapsed/refractory classic Hodgkin lymphoma. J. Clin. Oncol..

[406] Westin JR (2014). Safety and activity of PD1 blockade by pidilizumab in combination with rituximab in patients with relapsed follicular lymphoma: a single group, open-label, phase 2 trial. Lancet Oncol..

[407] Advani R (2018). CD47 blockade by Hu5F9-G4 and rituximab in non-Hodgkin’s lymphoma. N. Engl. J. Med..

[408] Armand P (2016). Programmed death-1 blockade with pembrolizumab in patients with classical Hodgkin lymphoma after brentuximab vedotin failure. J. Clin. Oncol..

[409] Younes A (2019). Safety and activity of ibrutinib in combination with nivolumab in patients with relapsed non-Hodgkin lymphoma or chronic lymphocytic leukaemia: a phase 1/2a study. Lancet Haematol..

[410] Ding W (2017). Pembrolizumab in patients with CLL and Richter transformation or with relapsed CLL. Blood.

[411] Zinzani PL (2017). Safety and tolerability of pembrolizumab in patients with relapsed/refractory primary mediastinal large B-cell lymphoma. Blood.

[412] Berger R (2008). Phase I safety and pharmacokinetic study of CT-011, a humanized antibody interacting with PD-1, in patients with advanced hematologic malignancies. Clin. Cancer Res..

[413] Hawkes E (2022). First-in-human (FIH) study of the fully-human kappa-lambda CD19/CD47 bispecific antibody TG-1801 in patients (pts) with B-cell lymphoma. Blood.

[414] Mehta A (2021). Lemzoparlimab, a differentiated anti-CD47 antibody in combination with rituximab in relapsed and refractory non- Hodgkin’s lymphoma: initial clinical results. Blood.

[415] Qi JY (2020). A phase I/IIa study of lemzoparlimab, a monoclonal antibody targeting CD47, in patients with relapsed and/or refractory acute myeloid leukemia (AML) and myelodysplastic syndrome (MDS): initial phase I results. Blood.

[416] Stadtmauer E (2021). Lemzoparlimab (TJ011133), an anti-CD47 antibody, with/without dexamethasone plus anti myeloma regimens for relapsed/refractory multiple myeloma: a phase 1b dose escalation and expansion study. J. Immunother. Cancer.

[417] Garcia-Manero G (2021). Evorpacept (ALX148), a CD47-blocking myeloid checkpoint inhibitor, in combination with azacitidine: a phase 1 / 2 study in patients with myelodysplastic syndrome (ASPEN-02). Blood.

[418] Maude SL (2018). Tisagenlecleucel in children and young adults with B-cell lymphoblastic leukemia. N. Engl. J. Med..

[419] Schuster SJ (2019). Tisagenlecleucel in adult relapsed or refractory diffuse large B-cell lymphoma. N. Engl. J. Med..

[420] Bishop MR (2022). Second-line tisagenlecleucel or standard care in aggressive B-cell lymphoma. N. Engl. J. Med..

[421] Fowler NH (2022). Tisagenlecleucel in adult relapsed or refractory follicular lymphoma: the phase 2 ELARA trial. Nat. Med..

[422] Locke FL (2019). Long-term safety and activity of axicabtagene ciloleucel in refractory large B-cell lymphoma (ZUMA-1): a single-arm, multicentre, phase 1-2 trial. Lancet Oncol..

[423] Wang M (2020). KTE-X19 CAR T-cell therapy in relapsed or refractory mantle-cell lymphoma. N. Engl. J. Med..

[424] Shah BD (2021). KTE-X19 for relapsed or refractory adult B-cell acute lymphoblastic leukaemia: phase 2 results of the single-arm, open-label, multicentre ZUMA-3 study. Lancet.

[425] Jacobson CA (2022). Axicabtagene ciloleucel in relapsed or refractory indolent non-Hodgkin lymphoma (ZUMA-5): a single-arm, multicentre, phase 2 trial. Lancet Oncol..

[426] Locke FL (2022). Axicabtagene ciloleucel as second-line therapy for large B-cell lymphoma. N. Engl. J. Med..

[427] Neelapu SS (2022). Axicabtagene ciloleucel as first-line therapy in high-risk large B-cell lymphoma: the phase 2 ZUMA-12 trial. Nat. Med..

[428] Siddiqi T (2022). Phase 1 TRANSCEND CLL 004 study of lisocabtagene maraleucel in patients with relapsed/refractory CLL or SLL. Blood.

[429] Kamdar M (2022). Lisocabtagene maraleucel versus standard of care with salvage chemotherapy followed by autologous stem cell transplantation as second-line treatment in patients with relapsed or refractory large B-cell lymphoma (TRANSFORM): results from an interim analysis of an open-label, randomised, phase 3 trial. Lancet.

[430] Sehgal A (2022). Lisocabtagene maraleucel as second-line therapy in adults with relapsed or refractory large B-cell lymphoma who were not intended for haematopoietic stem cell transplantation (PILOT): an open-label, phase 2 study. Lancet Oncol..

[431] Cheng Q (2022). CD20-specific chimeric antigen receptor-expressing T cells as salvage therapy in rituximab-refractory/relapsed B-cell non-Hodgkin lymphoma. Cytotherapy.

[432] Fry TJ (2018). CD22-targeted CAR T cells induce remission in B-ALL that is naive or resistant to CD19-targeted CAR immunotherapy. Nat. Med..

[433] Spiegel JY (2021). CAR T cells with dual targeting of CD19 and CD22 in adult patients with recurrent or refractory B cell malignancies: a phase 1 trial. Nat. Med..

[434] Shah NN (2020). Bispecific anti-CD20, anti-CD19 CAR T cells for relapsed B cell malignancies: a phase 1 dose escalation and expansion trial. Nat. Med..

[435] Ramos CA (2020). Anti-CD30 CAR-T cell therapy in relapsed and refractory Hodgkin lymphoma. J. Clin. Oncol..

[436] Ramos CA (2017). Clinical and immunological responses after CD30-specific chimeric antigen receptor-redirected lymphocytes. J. Clin. Investig..

[437] Wang CM (2017). Autologous T cells expressing CD30 chimeric antigen receptors for relapsed or refractory Hodgkin lymphoma: an open-label phase I trial. Clin. Cancer Res..

[438] Raje N (2019). Anti-BCMA CAR T-cell therapy bb2121 in relapsed or refractory multiple myeloma. N. Engl. J. Med..

[439] Munshi NC (2021). Idecabtagene vicleucel in relapsed and refractory multiple myeloma. N. Engl. J. Med..

[440] Rodriguez-Otero P (2023). Ide-cel or standard regimens in relapsed and refractory multiple myeloma. N. Engl. J. Med..

[441] Raje N (2022). Updated clinical and correlative results from the Phase I CRB-402 study of the BCMA-targeted CAR T cell therapy bb21217 in patients with relapsed and/or refractory multiple myeloma. Clin. Lymphoma Myeloma Leuk..

[442] Berdeja JG (2021). Ciltacabtagene autoleucel, a B-cell maturation antigen-directed chimeric antigen receptor T-cell therapy in patients with relapsed or refractory multiple myeloma (CARTITUDE-1): a phase 1b/2 open-label study. Lancet.

[443] Costello CL (2019). Phase 2 study of the response and safety of P-Bcma-101 CAR-T cells in patients with relapsed/refractory (r/r) multiple myeloma (MM) (PRIME). Blood.

[444] Mailankody S (2020). Orvacabtagene autoleucel (orva-cel), a B-cell maturation antigen (BCMA)-directed CAR T cell therapy for patients (pts) with relapsed/refractory multiple myeloma (RRMM): update of the phase 1/2 EVOLVE study (NCT03430011). J. Clin. Oncol..

[445] Kumar SK (2020). Results from Lummicar-2: a phase 1b/2 study of fully human B-cell maturation antigen-specific CAR T cells (CT053) in patients with relapsed and/or refractory multiple myeloma. Blood.

[446] Mailankody S (2023). Allogeneic BCMA-targeting CAR T cells in relapsed/refractory multiple myeloma: phase 1 UNIVERSAL trial interim results. Nat. Med..

[447] Zhao WH (2018). A phase 1, open-label study of LCAR-B38M, a chimeric antigen receptor T cell therapy directed against B cell maturation antigen, in patients with relapsed or refractory multiple myeloma. J. Hematol. Oncol..

[448] Bal S (2022). Clinical activity of BMS-986393 (CC-95266), a G protein-coupled receptor class C group 5 member D (GPRC5D)-targeted chimeric antigen receptor (CAR) T cell therapy, in patients with relapsed and/or refractory (R/R) multiple myeloma (MM): first results from a phase 1, multicenter, open-label study. Blood.

[449] Shi D (2022). USP14 promotes tryptophan metabolism and immune suppression by stabilizing IDO1 in colorectal cancer. Nat. Commun..

[450] Yan Z (2019). A combination of humanised anti-CD19 and anti-BCMA CAR T cells in patients with relapsed or refractory multiple myeloma: a single-arm, phase 2 trial. Lancet Haematol..

[451] Baumeister SH (2019). Phase I trial of autologous CAR T cells targeting NKG2D ligands in patients with AML/MDS and multiple myeloma. Cancer Immunol. Res..

[452] Jin X (2022). First-in-human phase I study of CLL-1 CAR-T cells in adults with relapsed/refractory acute myeloid leukemia. J. Hematol. Oncol..

[453] Sallman DA (2022). Ameli-01: a phase I trial of UCART123v1.2, an anti-CD123 allogeneic CAR-T cell product, in adult patients with relapsed or refractory (R/R) CD123+acute myeloid leukemia (AML). Blood.

[454] Lu P (2022). Naturally selected CD7 CAR-T therapy without genetic manipulations for T-ALL/LBL: first-in-human phase 1 clinical trial. Blood.

[455] Zhang Y (2023). Allogenic and autologous anti-CD7 CAR-T cell therapies in relapsed or refractory T-cell malignancies. Blood Cancer J..

[456] Lekakis LJ (2021). ALPHA2 Study: ALLO-501A allogeneic CAR T in LBCL, updated results continue to show encouraging safety and efficacy with consolidation dosing. Blood.

[457] Jain N (2021). Preliminary results from the Flu/Cy/alemtuzumab arm of the phase I BALLI-01 trial of UCART22, an antiCD22 allogeneic CAR-T cell product, in adult patients with relapsed or refractory (R/R) CD22+B-cell acute lymphoblastic leukemia (B-ALL). Blood.

[458] Ni JJ (2022). Immune-based combination therapy to convert immunologically cold tumors into hot tumors: an update and new insights. Acta Pharmacol. Sin..

[459] McGuirk JP (2022). CTX110 allogeneic CRISPR-Cas9-engineered CAR T cells in patients (pts) with relapsed or refractory (R/R) large B-cell lymphoma (LBCL): results from the phase 1 dose escalation carbon study. Blood.

[460] Shah BD (2021). Preliminary safety and efficacy of PBCAR0191, an allogeneic, off-the-shelf CD19-targeting CAR-T product, in relapsed/refractory (r/r) CD19+NHL. J. Clin. Oncol..

[461] Jain N (2021). Preliminary safety and efficacy of PBCAR0191, an allogeneic ‘off-the-shelf’ CD19-directed CAR-T for patients with relapsed/refractory (R/R) CD19+B-ALL. Blood.

[462] Scott DW, Gascoyne RD (2014). The tumour microenvironment in B cell lymphomas. Nat. Rev. Cancer.

[463] Sang W (2022). Anti-PD-1 therapy enhances the efficacy of CD30-directed chimeric antigen receptor T cell therapy in patients with relapsed/refractory CD30+ lymphoma. Front. Immunol..

[464] Chen X (2020). A Phase I clinical trial of chimeric antigen receptor-modified T cells in patients with relapsed and refractory lymphoma. Immunotherapy.

[465] Zhang J (2022). Non-viral, specifically targeted CAR-T cells achieve high safety and efficacy in B-NHL. Nature.

[466] Liu H (2021). CD19-specific CAR T Cells that express a PD-1/CD28 chimeric switch-receptor are effective in patients with PD-L1-positive B-cell lymphoma. Clin. Cancer Res..

[467] Cruz CR (2013). Infusion of donor-derived CD19-redirected virus-specific T cells for B-cell malignancies relapsed after allogeneic stem cell transplant: a phase 1 study. Blood.

[468] Wang X (2016). Phase 1 studies of central memory-derived CD19 CAR T-cell therapy following autologous HSCT in patients with B-cell NHL. Blood.

[469] Zhao H (2020). Pre-transplant MRD negativity predicts favorable outcomes of CAR-T therapy followed by haploidentical HSCT for relapsed/refractory acute lymphoblastic leukemia: a multi-center retrospective study. J. Hematol. Oncol..

[470] Jiang H (2019). Anti-CD19 chimeric antigen receptor-modified T-cell therapy bridging to allogeneic hematopoietic stem cell transplantation for relapsed/refractory B-cell acute lymphoblastic leukemia: An open-label pragmatic clinical trial. Am. J. Hematol..

[471] Liu E (2020). Use of CAR-transduced natural killer cells in CD19-positive lymphoid tumors. N. Engl. J. Med..

[472] Mickinso M (2021). A phase 1 study of NKX019, a CD19 chimeric antigen receptor natural killer (CAR NK) cell therapy, in subjects with B-cell malignancies. Blood.

[473] Bachier C (2020). A phase 1 study of NKX101, an allogeneic CAR natural killer (NK) cell therapy, in subjects with relapsed/refractory (R/R) acute myeloid leukemia (AML) or higher-risk myelodysplastic syndrome (MDS). Blood.

[474] Dhakal B (2022). Interim phase I clinical data of FT576 as monotherapy and in combination with daratumumab in subjects with relapsed/refractory multiple myeloma. Blood.

[475] Roex G (2022). Two for one: targeting BCMA and CD19 in B-cell malignancies with off-the-shelf dual-CAR NK-92 cells. J. Transl. Med..

[476] Christodoulou I (2021). Engineering CAR-NK cells to secrete IL-15 sustains their anti-AML functionality but is associated with systemic toxicities. J. Immunother. Cancer.

[477] Gong Y (2021). Chimeric antigen receptor natural killer (CAR-NK) cell design and engineering for cancer therapy. J. Hematol. Oncol..

[478] Kennedy LB, Salama AKS (2020). A review of cancer immunotherapy toxicity. CA Cancer J. Clin..

[479] Fontana L, Strasfeld L (2019). Respiratory virus infections of the stem cell transplant recipient and the hematologic malignancy patient. Infect. Dis. Clin. North Am..

[480] Misch EA, Andes DR (2019). Bacterial infections in the stem cell transplant recipient and hematologic malignancy patient. Infect. Dis. Clin. North Am..

[481] Nathan S, Ustun C (2019). Complications of stem cell transplantation that affect infections in stem cell transplant recipients, with analogies to patients with hematologic malignancies. Infect. Dis. Clin. North Am..

[482] Renaghan AD (2020). Acute kidney injury and CKD associated with hematopoietic stem cell transplantation. Clin. J. Am. Soc. Nephrol..

[483] Kemmner S, Verbeek M, Heemann U (2017). Renal dysfunction following bone marrow transplantation. J. Nephrol..

[484] Abramson MH (2021). Acute kidney injury in the modern era of allogeneic hematopoietic stem cell transplantation. Clin. J. Am. Soc. Nephrol..

[485] Mahadeo KM (2020). Diagnosis, grading, and treatment recommendations for children, adolescents, and young adults with sinusoidal obstructive syndrome: an international expert position statement. Lancet Haematol..

[486] Bonifazi F (2020). Diagnosis and treatment of VOD/SOS after allogeneic hematopoietic stem cell transplantation. Front. Immunol..

[487] Kammersgaard MB (2019). Assessment of the proposed EBMT pediatric criteria for diagnosis and severity grading of sinusoidal obstruction syndrome. Bone Marrow Transpl..

[488] Dignan FL (2013). BCSH/BSBMT guideline: diagnosis and management of veno-occlusive disease (sinusoidal obstruction syndrome) following haematopoietic stem cell transplantation. Br. J. Haematol..

[489] Markey KA, MacDonald KP, Hill GR (2014). The biology of graft-versus-host disease: experimental systems instructing clinical practice. Blood.

[490] Ferrara JL, Deeg HJ (1991). Graft-versus-host disease. N. Engl. J. Med..

[491] Sacirbegovic F (2023). Graft-versus-host disease is locally maintained in target tissues by resident progenitor-like T cells. Immunity.

[492] Khoury HJ (2017). Improved survival after acute graft-versus-host disease diagnosis in the modern era. Haematologica.

[493] Zhao XS, Huang XJ (2019). Seeking biomarkers for acute graft-versus-host disease: where we are and where we are heading?. Biomark. Res..

[494] Gooptu M, Koreth J (2017). Better acute graft-versus-host disease outcomes for allogeneic transplant recipients in the modern era: a tacrolimus effect?. Haematologica.

[495] Müller AMS (2022). Chronic GVHD on the move. Blood.

[496] Kong X (2022). Trafficking between clonally related peripheral T-helper cells and tissue-resident T-helper cells in chronic GVHD. Blood.

[497] Nelson AS (2015). Second cancers and late mortality in Australian children treated by allogeneic HSCT for haematological malignancy. Leukemia.

[498] Goyal A (2020). Increased risk of second primary hematologic and solid malignancies in patients with mycosis fungoides: A Surveillance, Epidemiology, And End Results analysis. J. Am. Acad. Dermatol..

[499] André M (1998). Treatment-related deaths and second cancer risk after autologous stem-cell transplantation for Hodgkin’s disease. Blood.

[500] Tichelli A (2019). Evaluation of second solid cancers after hematopoietic stem cell transplantation in European patients. JAMA Oncol..

[501] Sirohi B (2008). Long-term outcome of autologous stem-cell transplantation in relapsed or refractory Hodgkin’s lymphoma. Ann. Oncol..

[502] Duignan S (2022). Post-transplant lymphoproliferative disorder presenting as supraglottitis following pediatric heart transplantation treated with EBV-specific cytotoxic T-lymphocytes. J. Heart Lung Transpl..

[503] Zimmermann H (2022). Modified risk-stratified sequential treatment (subcutaneous rituximab with or without chemotherapy) in B-cell Post-transplant lymphoproliferative disorder (PTLD) after Solid organ transplantation (SOT): the prospective multicentre phase II PTLD-2 trial. Leukemia.

[504] Thieme CJ (2022). In vitro and in vivo evidence that the switch from calcineurin to mTOR inhibitors may be a strategy for immunosuppression in Epstein-Barr virus-associated post-transplant lymphoproliferative disorder. Kidney Int.

[505] Trappe R (2012). Sequential treatment with rituximab followed by CHOP chemotherapy in adult B-cell post-transplant lymphoproliferative disorder (PTLD): the prospective international multicentre phase 2 PTLD-1 trial. Lancet Oncol..

[506] Burns DM (2020). Real-world outcomes with rituximab-based therapy for posttransplant lymphoproliferative disease arising after solid organ transplant. Transplantation.

[507] Byrd JC (1999). Rituximab therapy in hematologic malignancy patients with circulating blood tumor cells: association with increased infusion-related side effects and rapid blood tumor clearance. J. Clin. Oncol..

[508] Hansel TT (2010). The safety and side effects of monoclonal antibodies. Nat. Rev. Drug Discov..

[509] Baldo BA (2013). Adverse events to monoclonal antibodies used for cancer therapy: Focus on hypersensitivity responses. Oncoimmunology.

[510] Duell J (2019). Functionally defective T cells after chemotherapy of B-cell malignancies can be activated by the tetravalent bispecific CD19/CD3 antibody AFM11. J. Immunother..

[511] Salvaris, R., Ong, J. & Gregory, G. P. Bispecific antibodies: a review of development, clinical efficacy and toxicity in B-cell lymphomas. *J. Pers. Med*. **11** (2021). 10.3390/jpm11050355 PMC8147062 33946635

[512] van Brummelen EM (2016). Antidrug antibody formation in oncology: clinical relevance and challenges. Oncologist.

[513] Zhu Y, Liu K, Wang K, Zhu H (2023). Treatment-related adverse events of antibody-drug conjugates in clinical trials: a systematic review and meta-analysis. Cancer.

[514] Nguyen TD, Bordeau BM, Balthasar JP (2023). Mechanisms of ADC toxicity and strategies to increase ADC tolerability. Cancers.

[515] Wolska-Washer A, Robak T (2019). Safety and tolerability of antibody-drug conjugates in cancer. Drug Saf..

[516] Thompson JA (2019). Management of immunotherapy-related toxicities, version 1.2019. J. Natl Compr. Canc. Netw..

[517] Haanen J (2017). Management of toxicities from immunotherapy: ESMO clinical practice guidelines for diagnosis, treatment and follow-up. Ann. Oncol..

[518] Thompson JA (2018). New NCCN guidelines: recognition and management of immunotherapy-related toxicity. J. Natl Compr. Cancer Netw..

[519] Haanen J (2018). Management of toxicities from immunotherapy: ESMO clinical practice guidelines for diagnosis, treatment and follow-up. Ann. Oncol..

[520] Puzanov I (2017). Managing toxicities associated with immune checkpoint inhibitors: consensus recommendations from the Society for Immunotherapy of Cancer (SITC) Toxicity Management Working Group. J. Immunother. Cancer.

[521] Schneider BJ, Lacchetti C, Bollin K (2022). Management of the top 10 most common immune-related adverse events in patients treated with immune checkpoint inhibitor therapy. JCO Oncol. Pract..

[522] Martins F (2019). New therapeutic perspectives to manage refractory immune checkpoint-related toxicities. Lancet Oncol..

[523] Belum VR (2016). Characterisation and management of dermatologic adverse events to agents targeting the PD-1 receptor. Eur. J. Cancer.

[524] Geisler AN (2020). Immune checkpoint inhibitor-related dermatologic adverse events. J. Am. Acad. Dermatol..

[525] Ellis SR (2020). Dermatologic toxicities to immune checkpoint inhibitor therapy: a review of histopathologic features. J. Am. Acad. Dermatol..

[526] Kumar V (2017). Current diagnosis and management of immune related adverse events (irAEs) induced by immune checkpoint inhibitor therapy. Front. Pharmacol..

[527] Villadolid J, Amin A (2015). Immune checkpoint inhibitors in clinical practice: update on management of immune-related toxicities. Transl. Lung Cancer Res..

[528] Phillips GS (2019). Treatment outcomes of immune-related cutaneous adverse events. J. Clin. Oncol..

[529] de Filette J (2019). A systematic review and meta-analysis of endocrine-related adverse events associated with immune checkpoint inhibitors. Horm. Metab. Res..

[530] Wright JJ, Powers AC, Johnson DB (2021). Endocrine toxicities of immune checkpoint inhibitors. Nat. Rev. Endocrinol..

[531] Grouthier V (2020). Immune checkpoint inhibitor-associated primary adrenal insufficiency: WHO vigiBase report analysis. Oncologist.

[532] Muir CA (2021). Thyroid immune-related adverse events following immune checkpoint inhibitor treatment. J. Clin. Endocrinol. Metab..

[533] de Filette JMK (2019). Immune checkpoint inhibitors and type 1 diabetes mellitus: a case report and systematic review. Eur. J. Endocrinol..

[534] Reynolds K, Thomas M, Dougan M (2018). Diagnosis and management of hepatitis in patients on checkpoint blockade. Oncologist.

[535] De Martin E (2018). Characterization of liver injury induced by cancer immunotherapy using immune checkpoint inhibitors. J. Hepatol..

[536] Zen Y, Yeh MM (2018). Hepatotoxicity of immune checkpoint inhibitors: a histology study of seven cases in comparison with autoimmune hepatitis and idiosyncratic drug-induced liver injury. Mod. Pathol..

[537] Abdel-Rahman O, ElHalawani H, Fouad M (2015). Risk of gastrointestinal complications in cancer patients treated with immune checkpoint inhibitors: a meta-analysis. Immunotherapy.

[538] Geukes Foppen MH (2018). Immune checkpoint inhibition-related colitis: symptoms, endoscopic features, histology and response to management. ESMO Open.

[539] Collins M (2017). Inflammatory gastrointestinal diseases associated with PD-1 blockade antibodies. Ann. Oncol..

[540] Samaan MA (2018). Gastrointestinal toxicity of immune checkpoint inhibitors: from mechanisms to management. Nat. Rev. Gastroenterol. Hepatol..

[541] Naidoo J (2017). Pneumonitis in patients treated with anti-programmed death-1/programmed death ligand 1 therapy. J. Clin. Oncol..

[542] Nishino M (2016). Incidence of programmed cell death 1 inhibitor-related pneumonitis in patients with advanced cancer: a systematic review and meta-analysis. JAMA Oncol..

[543] Naidoo J (2020). Chronic immune checkpoint inhibitor pneumonitis. J. Immunother. Cancer.

[544] Delaunay M (2019). Management of pulmonary toxicity associated with immune checkpoint inhibitors. Eur. Respir. Rev..

[545] Zhang XT, Ge N, Xiang ZJ, Liu T (2022). Immune checkpoint inhibitor-related adverse cardiac events in patients with lung cancer: a systematic review and meta-analysis. Cancer Cell Int..

[546] Solimando, A. G. et al. Immune checkpoint inhibitor-related myositis: from biology to bedside. *Int. J. Mol. Sci*. **21**, 3054 (2020). 10.3390/ijms21093054 PMC7246673 32357515

[547] Wu L (2021). Unravelling checkpoint inhibitor associated autoimmune diabetes: from bench to bedside. Front. Endocrinol..

[548] Kramer R (2021). Hematological immune related adverse events after treatment with immune checkpoint inhibitors. Eur. J. Cancer.

[549] Herrmann SM, Perazella MA (2020). Immune checkpoint inhibitors and immune-related adverse renal events. Kidney Int. Rep..

[550] Sprangers B (2022). Diagnosis and management of immune checkpoint inhibitor-associated acute kidney injury. Nat. Rev. Nephrol..

[551] Meraz-Muñoz A (2020). Acute kidney injury associated with immune checkpoint inhibitor therapy: incidence, risk factors and outcomes. J. Immunother. Cancer.

[552] Awadalla M (2020). Global longitudinal strain and cardiac events in patients with immune checkpoint inhibitor-related myocarditis. J. Am. Coll. Cardiol..

[553] Leaf RK (2019). Clinical and laboratory features of autoimmune hemolytic anemia associated with immune checkpoint inhibitors. Am. J. Hematol..

[554] Haanen J (2020). Rechallenge patients with immune checkpoint inhibitors following severe immune-related adverse events: review of the literature and suggested prophylactic strategy. J. Immunother. Cancer.

[555] Schneider BJ (2021). Management of immune-related adverse events in patients treated with immune checkpoint inhibitor therapy: ASCO guideline update. J. Clin. Oncol..

[556] Norelli M (2018). Monocyte-derived IL-1 and IL-6 are differentially required for cytokine-release syndrome and neurotoxicity due to CAR T cells. Nat. Med..

[557] Sandler RD (2020). Diagnosis and management of secondary HLH/MAS folowing HSCT and CAR-T cell therapy in adults; a review of the literature and a survey of practice within EBMT centres on behalf of the Autoimmune Diseases Working Party (ADWP) and Transplant Complications Working Party (TCWP). Front. Immunol..

[558] Lichtenstein DA (2021). Characterization of HLH-like manifestations as a CRS variant in patients receiving CD22 CAR T cells. Blood.

[559] Schubert ML (2021). Side-effect management of chimeric antigen receptor (CAR) T-cell therapy. Ann. Oncol..

[560] Si X (2022). Hematologic cytopenia post CAR T cell therapy: etiology, potential mechanisms and perspective. Cancer Lett..

[561] Morgan RA (2010). Case report of a serious adverse event following the administration of T cells transduced with a chimeric antigen receptor recognizing ERBB2. Mol. Ther..

[562] Gust J, Taraseviciute A, Turtle CJ (2018). Neurotoxicity associated with CD19-targeted CAR-T cell therapies. CNS Drugs.

[563] Santomasso BD (2018). Clinical and biological correlates of neurotoxicity associated with CAR T-cell therapy in patients with B-cell acute lymphoblastic leukemia. Cancer Discov..

[564] Brudno JN, Kochenderfer JN (2019). Recent advances in CAR T-cell toxicity: Mechanisms, manifestations and management. Blood Rev..

[565] Thompson JA (2022). Management of immunotherapy-related toxicities, version 1.2022, NCCN clinical practice guidelines in oncology. J. Natl Compr. Canc. Netw..

[566] Morris EC, Neelapu SS, Giavridis T, Sadelain M (2022). Cytokine release syndrome and associated neurotoxicity in cancer immunotherapy. Nat. Rev. Immunol..

[567] Freyer CW, Porter DL (2020). Cytokine release syndrome and neurotoxicity following CAR T-cell therapy for hematologic malignancies. J. Allergy Clin. Immunol..

[568] Schmidts A, Wehrli M, Maus MV (2021). Toward better understanding and management of CAR-T cell-associated toxicity. Annu. Rev. Med..

[569] Santomasso BD (2021). Management of immune-related adverse events in patients treated with chimeric antigen receptor T-cell therapy: ASCO guideline. J. Clin. Oncol..

[570] Jain, M. D., Smith, M., Shah, N. N. How I treat refractory CRS and ICANS following CAR T-cell therapy. *Blood*. **141**, 2430–2442 (2023). 10.1182/blood.2022017414 PMC10329191 36989488

[571] Frey N, Porter D (2019). Cytokine release syndrome with chimeric antigen receptor T cell therapy. Biol. Blood Marrow Transplant..

[572] Giavridis T (2018). CAR T cell-induced cytokine release syndrome is mediated by macrophages and abated by IL-1 blockade. Nat. Med..

[573] Staedtke V (2018). Disruption of a self-amplifying catecholamine loop reduces cytokine release syndrome. Nature.

[574] Teachey DT (2016). Identification of predictive biomarkers for cytokine release syndrome after chimeric sntigen receptor T-cell therapy for acute lymphoblastic leukemia. Cancer Discov..

[575] Lee DW (2014). Current concepts in the diagnosis and management of cytokine release syndrome. Blood.

[576] Boyiadzis MM (2018). Chimeric antigen receptor (CAR) T therapies for the treatment of hematologic malignancies: clinical perspective and significance. J. Immunother. Cancer.

[577] Neelapu SS (2017). Axicabtagene ciloleucel CAR T-cell therapy in refractory large B-cell lymphoma. N. Engl. J. Med..

[578] Mueller KT (2018). Clinical pharmacology of tisagenlecleucel in B-cell acute lymphoblastic leukemia. Clin. Cancer Res.

[579] Wei J (2020). The model of cytokine release syndrome in CAR T-cell treatment for B-cell non-Hodgkin lymphoma. Signal Transduct. Target Ther..

[580] Grupp SA (2013). Chimeric antigen receptor-modified T cells for acute lymphoid leukemia. N. Engl. J. Med..

[581] Maude SL (2014). Chimeric antigen receptor T cells for sustained remissions in leukemia. N. Engl. J. Med..

[582] Le RQ (2018). FDA approval summary: tocilizumab for treatment of chimeric antigen receptor T cell-induced severe or life-threatening cytokine release syndrome. Oncologist.

[583] Chen H (2019). Management of cytokine release syndrome related to CAR-T cell therapy. Front. Med..

[584] Neelapu SS (2018). Chimeric antigen receptor T-cell therapy - assessment and management of toxicities. Nat. Rev. Clin. Oncol..

[585] Gust J (2017). Endothelial activation and blood-brain barrier disruption in neurotoxicity after adoptive immunotherapy with CD19 CAR-T cells. Cancer Discov..

[586] Taraseviciute A (2018). Chimeric antigen receptor T cell-mediated neurotoxicity in nonhuman primates. Cancer Discov..

[587] Lee DW (2019). ASTCT consensus grading for cytokine release syndrome and nurologic toxicity associated with immune efector clls. Biol. Blood Marrow Transpl..

[588] Santomasso BD, Gust J, Perna F (2023). How I treat unique and difficult to manage cases of CAR T-cell therapy associated neurotoxicity. Blood.

[589] Major A (2021). Management of hemophagocytic lymphohistiocytosis (HLH) associated with chimeric antigen receptor T-cell (CAR-T) therapy using anti-cytokine therapy: an illustrative case and review of the literature. Leuk. Lymphoma.

[590] Schram AM, Berliner N (2015). How I treat hemophagocytic lymphohistiocytosis in the adult patient. Blood.

[591] Sandler RD (2020). Diagnosis and management of secondary HLH/MAS following HSCT and CAR-T cell therapy in adults; a review of the literature and a survey of practice within EBMT centres on behalf of the Autoimmune Diseases Working Party (ADWP) and Transplant Complications Working Party (TCWP). Front. Immunol..

[592] Doan, A. & Pulsipher, M. A. Hypogammaglobulinemia due to CAR T-cell therapy. *Pediatr. Blood Cancer*10.1002/pbc.26914 (2018). 10.1002/pbc.26914 PMC7477537 29230962

[593] Wat J, Barmettler S (2022). Hypogammaglobulinemia after chimeric antigen receptor (CAR) T-cell therapy: characteristics, management, and future directions. J. Allergy Clin. Immunol. Pract..

[594] Garcia-Lloret M, McGhee S, Chatila TA (2008). Immunoglobulin replacement therapy in children. Immunol. Allergy Clin. North Am..

[595] Radinsky S, Bonagura VR (2003). Subcutaneous immunoglobulin infusion as an alternative to intravenous immunoglobulin. J. Allergy Clin. Immunol..

[596] Rejeski K (2021). CAR-HEMATOTOX: a model for CAR T-cell-related hematologic toxicity in relapsed/refractory large B-cell lymphoma. Blood.

[597] Jain, T., Olson, T. S., Locke, F. L. How I treat cytopenias after CAR T-cell therapy. *Blood* **141**, 2460–2469 (2023). 10.1182/blood.2022017415 PMC10646792 36800563

[598] Du M (2022). Case report: ITP treatment after CAR-T cell therapy in patients with multiple myeloma. Front. Immunol..

[599] Fried S (2019). Early and late hematologic toxicity following CD19 CAR-T cells. Bone Marrow Transpl..

[600] Nahas GR (2020). Incidence and risk factors associated with a syndrome of persistent cytopenias after CAR-T cell therapy (PCTT). Leuk. Lymphoma.

[601] Jain T (2020). Hematopoietic recovery in patients receiving chimeric antigen receptor T-cell therapy for hematologic malignancies. Blood Adv..

[602] Baur R (2021). Thrombopoietin receptor agonists for acquired thrombocytopenia following anti-CD19 CAR-T-cell therapy: a case report. J. Immunother. Cancer.

[603] Buechner J (2021). Practical guidelines for monitoring and management of coagulopathy following tisagenlecleucel CAR T-cell therapy. Blood Adv..

[604] Gudiol C, Lewis RE, Strati P, Kontoyiannis DP (2021). Chimeric antigen receptor T-cell therapy for the treatment of lymphoid malignancies: is there an excess risk for infection?. Lancet Haematol..

[605] Wang Y (2023). Brexucabtagene autoleucel for relapsed or refractory mantle cell lymphoma in standard-of-care practice: results from the US lymphoma CAR T consortium. J. Clin. Oncol..

[606] Hill JA (2018). Infectious complications of CD19-targeted chimeric antigen receptor-modified T-cell immunotherapy. Blood.

[607] Gill S, Brudno JN (2021). CAR T-cell therapy in hematologic malignancies: clinical role, toxicity, and unanswered questions. Am. Soc. Clin. Oncol. Educ. Book.

[608] Shi M (2022). Safety and efficacy of a humanized CD19 chimeric antigen receptor T cells for relapsed/refractory acute lymphoblastic leukemia. Am. J. Hematol..

[609] Holland EM (2022). Efficacy of second CAR-T (CART2) infusion limited by poor CART expansion and antigen modulation. J. Immunother. Cancer.

[610] Lamers CH (2011). Immune responses to transgene and retroviral vector in patients treated with ex vivo-engineered T cells. Blood.

[611] Xu J (2019). Exploratory trial of a biepitopic CAR T-targeting B cell maturation antigen in relapsed/refractory multiple myeloma. Proc. Natl Acad. Sci. USA.

[612] Wagner DL (2021). Immunogenicity of CAR T cells in cancer therapy. Nat. Rev. Clin. Oncol..

[613] Herrmann M (2018). Bifunctional PD-1 × αCD3 × αCD33 fusion protein reverses adaptive immune escape in acute myeloid leukemia. Blood.

[614] Correnti CE (2018). Simultaneous multiple interaction T-cell engaging (SMITE) bispecific antibodies overcome bispecific T-cell engager (BiTE) resistance via CD28 co-stimulation. Leukemia.

[615] Li X (2023). Decitabine priming increases anti-PD-1 antitumor efficacy by promoting CD8+ progenitor exhausted T cell expansion in tumor models. J. Clin. Invest..

[616] Wang Y (2021). Low-dose decitabine priming endows CAR T cells with enhanced and persistent antitumour potential via epigenetic reprogramming. Nat. Commun..

[617] Xu Y (2022). Epi-immunotherapy for cancers: rationales of epi-drugs in combination with immunotherapy and advances in clinical trials. Cancer Commun..

[618] Short NJ, Kantarjian H (2023). Using immunotherapy and novel trial designs to optimise front-line therapy in adult acute lymphoblastic leukaemia: breaking with the traditions of the past. Lancet Haematol..

[619] Yang J (2023). Advancing CAR T cell therapy through the use of multidimensional omics data. Nat. Rev. Clin. Oncol..

[620] Svensson V, Vento-Tormo R, Teichmann SA (2018). Exponential scaling of single-cell RNA-seq in the past decade. Nat. Protoc..

[621] Stubbington MJT, Rozenblatt-Rosen O, Regev A, Teichmann SA (2017). Single-cell transcriptomics to explore the immune system in health and disease. Science.

[622] Horns F, Dekker CL, Quake SR (2020). Memory B cell activation, broad anti-influenza antibodies, and bystander activation revealed by single-cell transcriptomics. Cell Rep..

[623] Bode D, Cull AH, Rubio-Lara JA, Kent DG (2021). Exploiting single-cell tools in gene and cell therapy. Front. Immunol..

[624] Castellanos-Rueda R, Di Roberto RB, Schlatter FS, Reddy ST (2021). Leveraging single-cell sequencing for chimeric antigen receptor T cell therapies. Trends Biotechnol..

[625] Tracy SI (2022). Combining nilotinib and PD-L1 blockade reverses CD4+ T-cell dysfunction and prevents relapse in acute B-cell leukemia. Blood.

[626] Jiang P (2022). Single-cell ATAC-seq maps the comprehensive and dynamic chromatin accessibility landscape of CAR-T cell dysfunction. Leukemia.

[627] Charitidis FT (2022). Monitoring CAR T cell generation with a CD8-targeted lentiviral vector by single-cell transcriptomics. Mol. Ther. Methods Clin. Dev..

[628] Bai Z (2021). Single-cell multiomics dissection of basal and antigen-specific activation states of CD19-targeted CAR T cells. J. Immunother. Cancer.

[629] Yan C (2021). Single-cell imaging of T cell immunotherapy responses in vivo. J. Exp. Med..

[630] Cazaux M (2019). Single-cell imaging of CAR T cell activity in vivo reveals extensive functional and anatomical heterogeneity. J. Exp. Med..

[631] Tian M (2022). An optimized bicistronic chimeric antigen receptor against GPC2 or CD276 overcomes heterogeneous expression in neuroblastoma. J. Clin. Invest..

[632] Pfeifer R (2022). A multimodal imaging workflow for monitoring CAR T cell therapy against solid tumor from whole-body to single-cell level. Theranostics.

